# Adaptations of *Pseudoxylaria* towards a comb-associated lifestyle in fungus-farming termite colonies

**DOI:** 10.1038/s41396-023-01374-4

**Published:** 2023-02-25

**Authors:** Janis Fricke, Felix Schalk, Nina B. Kreuzenbeck, Elena Seibel, Judith Hoffmann, Georg Dittmann, Benjamin H. Conlon, Huijuan Guo, Z. Wilhelm de Beer, Daniel Giddings Vassão, Gerd Gleixner, Michael Poulsen, Christine Beemelmanns

**Affiliations:** 1 Group Chemical Biology of Microbe-Host Interactions, Leibniz Institute for Natural Product Research and Infection Biology—Hans Knöll Institute (HKI), Beutenbergstraße 11a, 07745 Jena, Germany; 2 Department of Biogeochemical Processes, Max Planck Institute for Biogeochemistry, Hans-Knöll-Straße 10, 07745 Jena, Germany; 3 Department of Biology, Section for Ecology and Evolution, University of Copenhagen, Universitetsparken 15, 2100 Copenhagen, Denmark; 4 Department of Biochemistry, Genetics and Microbiology, Forestry and Agricultural Biotechnology Institute (FABI), University of Pretoria, Hatfield, 0028 Pretoria, South Africa; 5 Department of Biochemistry, Max Planck Institute for Chemical Ecology, Hans-Knöll-Straße 8, 07745 Jena, Germany; 6 Department Anti-infectives from Microbiota, Helmholtz Institute for Pharmaceutical Research Saarland (HIPS), Helmholtz Centre for Infection Research, Saarland University Campus, 66123 Saarbrücken, Germany; 7 Saarland University, 66123 Saarbrücken, Germany

**Keywords:** Symbiosis, Functional genomics, Microbial ecology, Metabolomics

## Abstract

Characterizing ancient clades of fungal symbionts is necessary for understanding the evolutionary process underlying symbiosis development. In this study, we investigated a distinct subgeneric taxon of *Xylaria* (*Xylariaceae*), named *Pseudoxylaria*, whose members have solely been isolated from the fungus garden of farming termites. *Pseudoxylaria* are inconspicuously present in active fungus gardens of termite colonies and only emerge in the form of vegetative stromata, when the fungus comb is no longer attended (“sit and wait” strategy). Insights into the genomic and metabolic consequences of their association, however, have remained sparse. Capitalizing on viable *Pseudoxylaria* cultures from different termite colonies, we obtained genomes of seven and transcriptomes of two *Pseudoxylaria* isolates. Using a whole-genome-based comparison with free-living members of the genus *Xylaria,* we document that the association has been accompanied by significant reductions in genome size, protein-coding gene content, and reduced functional capacities related to oxidative lignin degradation, oxidative stress responses and secondary metabolite production. Functional studies based on growth assays and fungus-fungus co-cultivations, coupled with isotope fractionation analysis, showed that *Pseudoxylaria* only moderately antagonizes growth of the termite food fungus *Termitomyces*, and instead extracts nutrients from the food fungus biomass for its own growth. We also uncovered that *Pseudoxylaria* is still capable of producing structurally unique metabolites, which was exemplified by the isolation of two novel metabolites, and that the natural product repertoire correlated with antimicrobial and insect antifeedant activity.

## Introduction

**Fig. 1:**
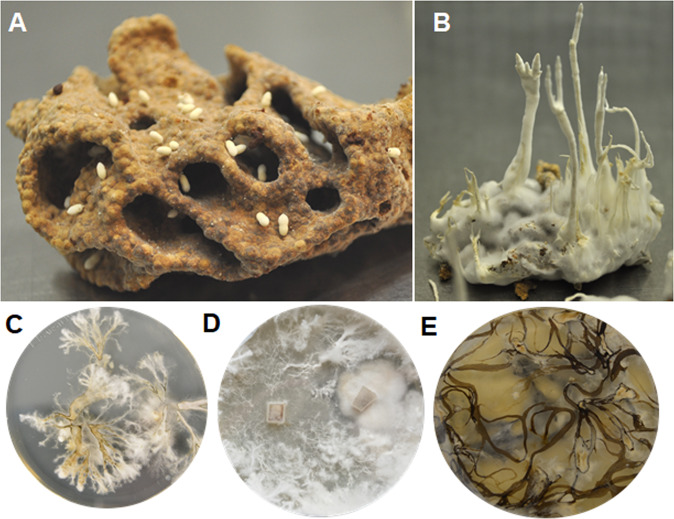
Natural growth and occurrence of fungal mutualist *Termitomyces* and termite-associated *Pseudoxylaria* strains. **A** Mature fungus comb from a *Macrotermes natalensis* colony with spore-containing fungal nodules of a *Termitomyces* strain, **B** *Pseudoxylaria* stromata emerging from fungus comb after incubation for eight days in the absence of termites, and **C**-**E** axenic fungal cultures of *Pseudoxylaria* strains isolated from different termite mounds grown on agar plates with, **C** *Pseudoxylaria* sp. Mn132, **D** *Pseudoxylaria* sp. X3-2, and **E** *Pseudoxylaria* sp. Mn153.

The Macrotermitinae are the only termite lineage to have acquired fungal symbionts from the genus *Termitomyces* (family Lyophyllaceae) as their food source [1–4]. *Termitomyces* is cultivated by workers in cork-like structures termed “fungus combs”, which are maintained in chambers located within the subterranean colony and are also known as “fungal gardens” [3]. To propagate the food fungus, younger workers ingest plant material alongside with *Termitomyces* biomass and use excreted lignocellulose and spore-enriched feces to craft new fungus comb on which *Termitomyces* is able to thrive (Fig. 1A). Termites have several levels of defense measures to protect this obligate nutritional symbiosis, starting with lower individual levels of hygiene measures to a higher collective level, also called social immunity [5–8]. Despite these preventive measures, fungal gardens inconspicuously host members of a distinct fungal subgenus of *Xylaria* (Ascomycota: Xylariaceae), commonly referred to as termite-associated *Pseudoxylaria* [9–15], which only emerge as vegetative stromata from comb material of deteriorating or inactive termite nests (Fig. 1B) [16]. While a number of studies have provided insights into their co-evolutionary relation with the fungus-farming termite symbiosis, the ecological role of *Pseudoxylaria* remains debated [1, 7]. Although few reports suggested a commensal role supporting biomass degradation within the comb environment [10, 17], other studies analyzing *Termitomyces*-*Xylaria* co-cultures hinted towards an antagonistic relation. As free-living *Xylaria* strains inhibited growth of *Termitomyces* more intensly than their termite-associated relatives [7, 18, 19], it was postulated that reduced antagonistic behavior might enable *Pseudo*x*ylaria* to evade the defense mechanisms of a healthy termite colony, and once conditions are favourable to outcompete the fungal mutualist [10–16].

Driven by the rather anecdotal evidence for the “reduced antagonism hypothesis” and the additional postulation that co-evolved *Pseudoxylaria* strains might have become a fungus garden substrate specialist over evolutionary time [11–13], we sequenced the genomes of seven and transcriptomes of two *Pseudoxylaria* isolates to investigate the genomic, transcriptomic and metabolomic basis for symbiotic associations. Whole genome-based comparison with free-living members of this genus uncovered a substantial reduction in genome size and numbers of protein-coding genes, as well as reduced functional capacities, all of which indicated that *Pseudoxylaria* might have become a dependent symbiont and comb-substrate specialist. By analyzing the secondary metabolite repertoire as well as co-cultivation studies along with isotope experiments, we were further able to solidify the “reduced antagonism hypothesis”.

## Results and discussion

### Genome reduction is associated with a termite comb-associated lifestyle

For our studies, we collected fungus comb samples originating from mounds of *Macrotermes natalensis*, *Odontotermes* spp., and *Microtermes* spp. termites and were able to obtain seven viable *Pseudoxylaria* cultures (X802 [*Microtermes* sp.], Mn132, Mn153, X187, X3-2 [*Macrotermes natalensis*], and X167, X170LB [*Odontotermes* spp.], Table S1-S3).

**Fig. 2:**
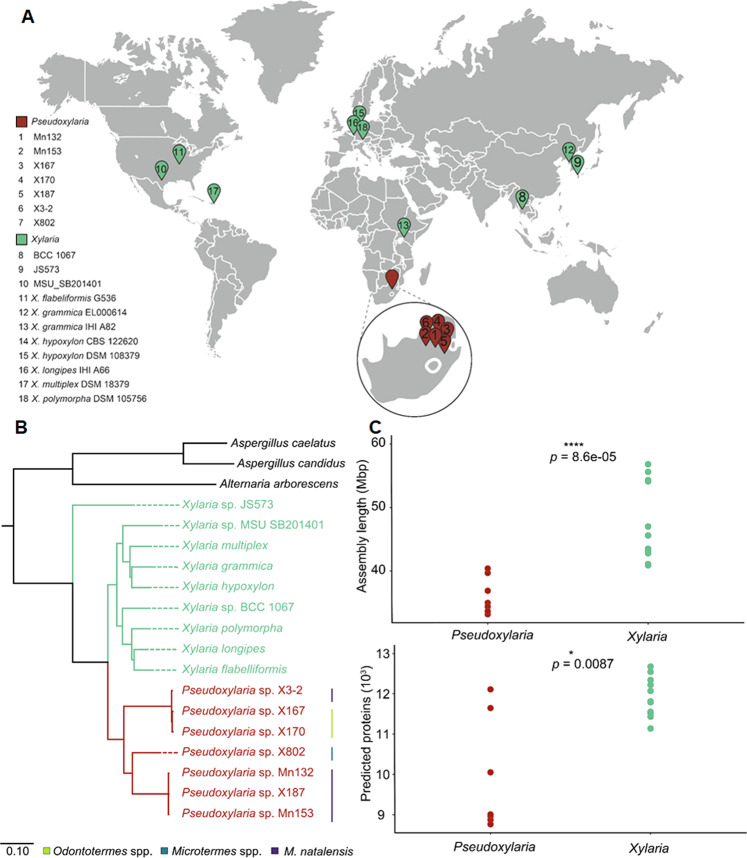
Geographic and comparative phylogenomic analysis of termite-associated *Pseudoxylaria* isolates (strains 1-7) and free-living *Xylaria* (strains 8–18). **A** Geographic origins of genome-sequenced free-living *Xylaria* and termite-associated *Pseudoxylaria* isolates, **B** phylogenomic placement based on single-copy ortholog protein sequences, and **C** comparison of genome assembly length, and numbers of predicted proteins per genome.

To test if a fungus comb-associated lifestyle of *Pseudoxylaria* was reflected in differences at the genome level, we sequenced the genomes of all seven isolates using a combination of paired-end shotgun sequencing (BGISEQ-500, BGI) and long-read sequencing (PacBio sequel, BGI or Oxford Nanopore Technologies, Oxford, UK). In addition, we sequenced the transcriptomes (BGISEQ, BGI) of two isolates (X802, X170LB). Eleven publicly available genomes of free-living *Xylaria* (Fig. 2A, B) were used as reference genomes (Table S4). Hybrid draft genomes were comprised on average of 33–742 scaffolds with total haploid assembly lengths of 33.2–40.4 Mb, and a high BUSCO completeness of genomes (> 95 %) with a total number of predicted proteins ranging from 8.8 to 12.1 × 10^3^. The GC content was comparable to reference genomes with 49.7–51.6%. To verify the phylogenetic placement of the isolates, different genetic loci encoding conserved protein sequences (α-actin (ACT), second largest subunit of RNA polymerase (RPB2), β-tubulin (TUB) and the internal transcribed spacer (ITS) were used as genetic markers [7, 13].

Phylogenies were reconstructed from ITS sequences and three aligned sequence datasets (RPB2, TUB, ACT) using reference sequences of twelve different taxa (Table S4–S7). Consistent with previous findings, all isolates grouped within the monophyletic termite-associated *Pseudoxylaria* group [9–13], which diverged from the free-living members of the genus *Xylaria* (Fig. 2B, Figure S1–S4).

As our seven isolates covered a larger portion of the previously reported phylogenetic diversity of the termite-associated subgenus, we elaborated on genomic characteristics of our isolates to uncover features of the termite-associated ecology of *Pseudoxylaria*. Indeed, comparative genome analysis of the South African *Pseudoxylaria* isolates with publicly available genomes of free-living *Xylaria* species of similar genome quality revealed significantly reduced genome assembly lengths in *Pseudoxylaria* with reduced numbers of predicted genes per genome (Table S4). Comparison of the annotated mitochondrial (mt) genomes (Figure S5, Table S8) also indicated that all seven mt genomes were shorter in length (assembly lengths: 18.5–63.8 kbp) compared to the, albeit few, publicly available mitochondrial genomes of free-living species (48.9–258.9 kbp). The reduction in mitochondrial genome size also corresponded to a significantly reduced mean number of annotated genes (7.6) and tRNAs (14.3) in *Pseudoxylaria* spp. compared to on average 30.0 (annotated genes) and 25.8 (tRNAs) found in free-living species.

Analysis of the abundance and composition of transposable elements (TEs), which account for up to 30–35% of the genomes of (endo)parasitic fungi due to the expansion of certain gene families [20, 21], showed that the mean total numbers of TEs across *Pseudoxylaria* spp. genomes were comparable (1530), but the numbers were reduced compared to free-living *Xylaria* species (3690) (Table S9). We also identified high variation in the TE composition across genomes (1.5–9.9 %), comparable to what was observed in free-living *Xylaria* spp. (1.3–8.1 %), with reductions in long terminal repeat retrotransposons (LTRs: Copia and unknown LTRs) in two inverted tandem repeat DNA transposons (TIRs; CACTA, Mutator and hAT). As *Pseudoxylaria* spp. contained increased numbers of non-ITR transposons of the helitron class and LTRs of the Gypsy class compared to *Xylaria* strains, we concluded that *Pseudoxylaria* exhibits no typical features of an (endo)parasitic lifestyle, but that the overall composition and the reduced numbers of TEs could serve as a fingerprint to distinguish the genetically divergent *Pseudoxylaria* taxa.

### Repertoire of carbohydrate-active enzymes indicates specialized substrate use

As the fungus comb is mostly composed of partially-digested plant material interspersed with fungal mycelium of the termite mutualist [3], we anticipated that *Pseudoxylaria* should exhibit features of a substrate specialist similar to the fungal mutualist *Termitomyces*, which should be reflected in a Carbohydrate-Active enzyme (CAZyme) repertoire distinguishable from  free-living saprophytic *Xylaria* species [22–24]. In particular, numbers and composition of redox-active enzymes (e.g., benzoquinone reductase (EC 1.6.5.6/EC 1.6.5.7), catalase (EC 1.11.1.6), glutathione reductase (EC 1.11.1.9), hydroxy acid oxidase (EC 1.1.3.15), laccase (EC 1.10.3.2), manganese peroxidase (EC 1.11.1.13), peroxiredoxin (EC 1.11.1.15), superoxide dismutase (EC 1.15.1.1), dye-decolorization or unspecific peroxygenase (EC 1.11.2.1), Table S10), which catalyze the degradation of lignin-rich biomass, were expected to differ between free-living strains and substrate specialists [22].

**Fig. 3:**
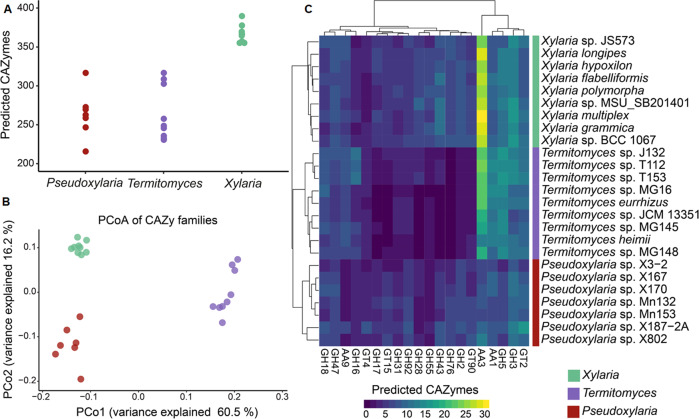
Comparison of carbohydrate-active enzymes (CAZymes) in *Xylaria*, *Pseudoxylaria* and the fungal mutualist *Termitomyces*. **A** Predicted CAZymes, **B** Principal Coordinates Analysis (PCoA) of predicted CAZyme families, and **C** heatmap of representatives CAZyme families in the predicted proteomes of free-living *Xylaria*, *Termitomyces* and *Pseudoxylaria* species.

Identification of CAZymes using Peptide Pattern Recognition (PPR) revealed that *Pseudoxylaria* genomes encoded on average a reduced number of CAZymes (mean 264) compared to the free-living taxa in the family *Xylaria* (mean 367 CAZymes, pANOVA; *F* = 41.4, *p* = 3.5 × 10^–8^_,_ pairwise *p* = 1.69 × 10^–7^) (Fig. 3A, B, Figure S6), but similar numbers to those identified in *Termitomyces* (mean 265, pairwise *p* = 0.949).

Overall, significant differences in the composition of CAZymes were observed [8], most notably in the reduction of auxiliary activity enzymes (AA), carbohydrate esterases (CE), glycosyl hydrolases (GH), and polysaccharide lyases (PL). The most significant reduction was observed in the AA3 family (Fig. 3C), which typically displays a high multigenicity in wood-degrading fungi as many  enzymes of this family catalyze the oxidation of alcohols or carbohydrates with the concomitant formation of hydrogen peroxide or hydroquinones thereby supporting lignocellulose degradation by other AA-enzymes, such as peroxidases (AA2). Similarly, although to a lesser extent, reduced numbers within the related AA1 family were detected, which included oxidizing enzymes like laccases, ferroxidases, and laccase-like multicopper oxidases. Along these lines, glycosyl hydrolases of the GH3 and GH5 family, including enzymes responsible for degradation of cellulose-containing biomass and xylose, were less abundant. We also noted that all *Pseudoxylaria* lacked homologs of the unspecific peroxygenases (UPO; EC 1.11.2.1), while almost all free-living *Xylaria* spp. and the fungal symbiont *Termitomyces* harbored at least one or two copies of similar gene sequences.

### *Pseudoxylaria* shows reduced biosynthetic capacity for secondary metabolite production

A healthy termite colony is engulfed in several layers of social immunity [5, 6], which pose a constant selection pressure on associated and potentially antagonistic microbes. As *Pseudoxylaria* evolved measures to remain inconspicuously present within the comb environment, we hypothesized that one of the possible adaptations to evade hygiene measures of termites could be reflected in a reduced biosynthetic capability to produce antibiotic or volatile natural products, which often serve as infochemicals triggering defense mechanisms [25–27], or as alarm pheromones [4, 28].

**Fig. 4:**
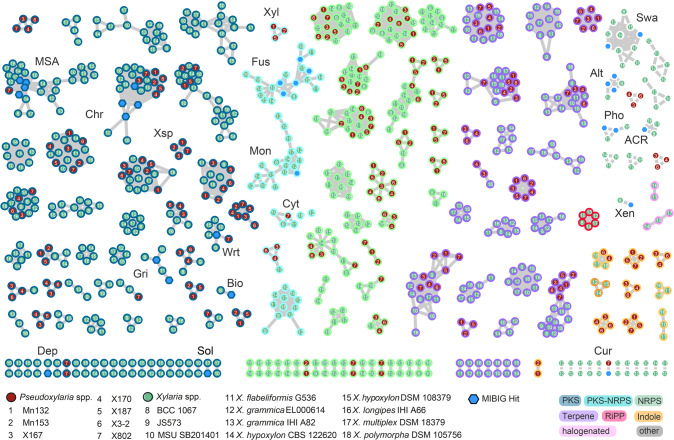
Similarity network analysis of biosynthetic gene clusters. Comparative analysis of termite associated-associated *Pseudoxylaria* isolates (strains 1–7, red circles) and free-living *Xylaria* (strains 8–18, green circles) with BiG-SCAPE 1.0 annotations (blue hexagon) ACR ACR toxin, Alt alternariol, Bio biotin, Chr chromene, Cyt cytochalasins, Cur curvupalide, Dep depiudecin, Fus fusarin, Gri griseofulvin, Mon monascorubin, MSA 6-methylsalicylic acid, Pho phomasetin, Sol solanapyrone, Swa swasionine, Xen xenolozoyenone, Xsp xylasporins, Xyl xylacremolide. Singletons are not shown.

The biosynthesis of secondary metabolites is encoded in so called Biosynthetic Gene Cluster (BGC) regions. We explored the abundance and diversity of encoded BGCs using FungiSMASH 6.0.0 and manually cross-checked the obtained data set by BLAST to account for possible biases due to varying genome qualities across strains of both groups [29]. Overall, the herein investigated *Xylaria* genomes harbored on average 90 BGCs per genome, while *Pseudoxylaria* encoded on average 45 BGCs (Fig. 4, Figure S7).

The nature and relatedness of the BGCs were analyzed by creating a curated similarity network analysis using BiG-SCAPE 1.0 [30]. Overall, 28 orthologous BGCs were shared across all genomes, including the biosynthesis of polyketides like 6-methylsalicylic acid (MSA), chromenes (Chr) and polyketide-non-ribosomal peptide (PKS-NRPS) hybrids like the cytochalasins (Cyt) [31]. Furthermore, five BGC networks, which were shared by *Pseudoxylaria* and *Xylaria*, contained genes encoding natural product modifying dimethylallyltryptophan synthases (DMATS). In contrast, and despite the significant reduction in the biosynthetic capacity within *Pseudoxylaria* genomes [29], about 29 BGC networks were unique to *Pseudoxylaria* and thus could possibly relate to the comb-associated lifestyle (Figure S8 and S9). Notably, *Pseudoxylaria* genomes lacked genes encoding ribosomally synthesized and posttranslationally modified peptides (RiPPs) or halogenases. In comparision, free-living *Xylaria* spp. harbored at least one sequence encoding a RiPP, and up to two orthologous sequences encoding putative halogenases. In contrast, a reduced average number of terpene synthases (TPS) in *Pseudoxylaria* (9 TPS) compared to free-living *Xylaria* (18 TPS) was detected, which included three BGCs encoding TPSs that were unique to *Pseudoxylaria*.  In comparison, genomes of the fungal mutualist *Termitomyces* were reported to encode for about 20-25 terpene cyclases, but haboured only about two loci containing genes for a PKS and NRPS each [24].

Manual BLAST searches were conducted to identify BGCs that could be putatively assigned to previously isolated metabolites from *Pseudoxylaria* (*vide infra* Fig. 7, Figure S8) [32, 33]. Using e.g., the known NRPS-PKS-hybrid cluster sequence *ccs* (*Aspergillus clavatus*) of cytochalasins as query, an orthologous BGC, here named *cytA*, was identified in the cytochalasin-producing strain X802 [34]. Although the putative PKS-NRPS hybrid and CcsA shared 60 % identical amino acids (aa), the sequences of the accessory enzymes were less related to CcsC-G (45–47% identical aa) and the BGC in X802 lacked a gene of a homologue to *ccsB*. Similarly, five free-living *Xylaria* species carried orthologous gene loci (*Xylaria* sp. BCC 1067, *Xylaria* sp. MSU_SB201401, *X. flabelliformis* G536, *X. grammica* EL000614, and *X. multiplex* DSM 110363) supporting previous isolation reports of cytochalasins with varying structural features. Furthermore, three *Pseudoxylaria* strains (X187, and closely related Mn153, and Mn132) were found to share a highly similar PKS-NRPS hybrid BGC (99–100 % identical aa, named *xya*), which likely encodes for the enzymatic production of previously identified xylacremolides [32]. Four *Pseudoxylaria* strains (X802, Mn132, Mn153, and X187) also shared a BGC (50–98 % amino acid identity) resembling the *fog* BGC (*Aspergillus ruber*) [35, 36], which putatively encodes the biosynthetic machinery to produce xylasporin/cytosporin-like metabolites. In this homology search, we also uncovered that *fog*-like BGC arrangements are likely more common than previously anticipated, as clusters with similar arrangements and identity were also found in genomes of *Rosellinia necatrix*, *Pseudomasariella vexata*, *Stachybotrys chartarum*, and *Hyaloscypha bicolor* (Fig. 4, Figure S8).

A detailed analysis of the *fog*-like cluster arrangements within *Pseudoxylaria* genomes revealed - similar to homologs of the *ccs* cluster – variation in the abundance and arrangement of several accessory genes coding for a cupin protein (*pxF*), a short chain oxidoreductase (*pxB*; SDR), and an additional SnoaL-like polyketide cyclase (*pxP*), which could account for the production of strain-specific structural congeners (*vide infra*, Fig. 7).

### Change of nutrient sources causes dedicated transcriptomic changes in *Pseudoxylaria*

To further solidify our in silico indications of substrate specialization with comb material as preferred substrate and fungus garden as environment, we analyzed *Pseudoxylaria* growth on different media (PDA, and reduced medium 1/3-PDA) including comb-like agar matrices (wood-rice medium (WRM), agar-agar or 1/3-PDA medium containing lyophilized (dead) *Termitomyces* sp. T112 biomass (T112, respectively T112-PDA), PDB covering glass-based surface-structuring elements (GB), Table S11–S14).

Cultivation of *Pseudoxylaria* on agar-agar containing lyophilized biomass of *Termitomyces* (T112) as the sole nutrient source allowed *Pseudoxylaria* to sustain growth, although to a reduced extent compared to growth on nutrient-rich PDA medium (Table S3). Wood-rice medium (WRM) induced comparable growth rates as observed on PDA and also the appearance of phenotypic stromata.

**Fig. 5:**
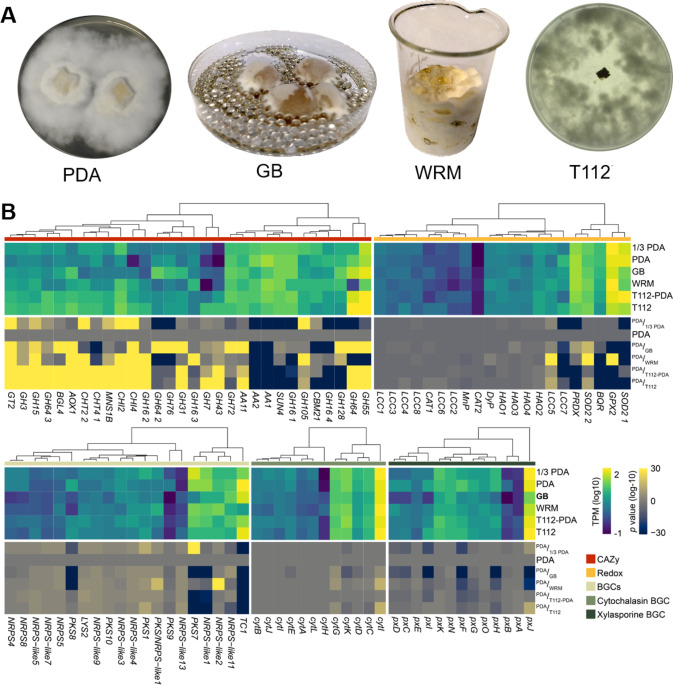
Transcriptomic analysis of *Pseudoxylaria* sp. X802 in dependence of growth conditions. **A** Representative pictures of *Pseudoxylaria* sp. X802 growing on PDA, PDB on glass beads (GB), wood-rice medium (WRM), and agar-agar medium containing lyophilized *Termitomyces* sp. T112 biomass (T112). **B** Heatmap of the most variable transcripts coding for CAZymes (red), redox enzymes (orange), secondary metabolite-related core genes (green), and more specifically on key genes within the boundaries of cytochalasin (turquoise) and xylasporin/cytosporin BGCs (blue). RNA was obtained from vegetative mycelium after growth on PDA, reduced medium (1/3-PDA), PDB on glass beads (GB), wood-rice medium (WRM), 1/3-PDA-medium enriched with *Termitomyces* sp. T112 biomass (T112-PDA) and agar-agar medium containing lyophilized *Termitomyces* biomass (T112). Transcript counts are shown as log_10_ transformed transcripts per million (top; TPM). Significance of the changes in transcript counts are compared to control (X802 grown on PDA) and depicted in log_-10_ transformed *p* values.

To investigate the influence of these growth conditions on the transcriptomic level, we harvested RNA from vegetative mycelium after growth on comb-like media (WRM, T112, T112-PDA, and GB), PDA, and reduced medium 1/3-PDA (Fig. 5A). The most significant transcript changes (normalized to data obtained from growth on PDA) were observed for genes coding for specific CAZymes including several redox active enzymes (Fig. 5B). The 30 most variable transcripts coded for specific glycoside hydrolases (GH), lytic polysaccharide monooxygenases (AA), ligninolytic enzymes, and a glycoside transferase (GT). Similarly, chitinases (CHT2; CHT4; CHI2; CHI4) were upregulated (up to 243-fold on T112) under almost all conditions compared to PDA, but some of these specific transcript changes were exclusive to growth on *Termitomyces* biomass or artificial comb material (WRM) suggesting the ability to regulate and increase chitin metabolism if necessary [37].

When X802 was grown on T112 (agar matrix containing lyophilized *Termitomyces* sp. T112 biomass), we observed a >400-fold increase in the expression of transcripts encoding glycoside hydrolases in the GH43 family, GH7 (~140-fold), GH3, and GH64 (5–12-fold). Similarly, transcripts for a putative mannosyl-oligosaccharide-α-1,2-mannosidase (MNS1B; 8.2-fold), chitinase CHT4 (2.9-fold), β-glucosidase BGL4 (5.7-fold), and copper-dependent lytic polysaccharide monooxygenase AA11 (1.6-fold) were significantly upregulated. Growth on WRM (wood-rice medium) or T112 (*Termitomyces* sp. T112 biomass) also caused a significant upregulation of genes coding for glycoside transferase GT2, glycoside hydrolases GH15, GH3, and aldehyde oxidase AOX1, which indicated the ability to expand the degradation portfolio if necessary. Along these lines, specific transcript levels were reduced when X802 was grown on T112, in particular class II lignin-modifying peroxidases (AA2), carbohydrate-binding module family 21 (CBM21), multicopper oxidases (AA1), secreted β-glucosidases (SUN4), and glycoside hydrolases GH16, and GH128.

When the fungus was challenged with lignocellulose-rich WRM medium, higher transcript levels putatively assigned to glutathione peroxidase (GXP2), superoxide dismutase (SOD2), and laccases (LCC5) were observed, which indicated that despite the reduced wood-degrading capacity, *Pseudoxylaria* activates available enzymatic mechanisms to degrade the provided material and respond to the resulting oxidative stress. Cultivation on GB (glass-based surfaces covered in liquid PD broth) influenced the expression of certain genes coding for glycoside hydrolases (GH64, GH76, GH72, GH128, BGL4) and lytic polysaccharide monooxygenases (AA1, AA2, AA11), presumably enabling the fungus to utilize soluble carbohydrates.

To test the hypothesis that the presence of *Termitomyces* biomass stimulates secondary metabolite production in *Pseudoxylaria* to eventually displace the mutualist, we also analyzed changes in the transcript levels of core BGC genes that encode the production of bioactive secondary metabolites. Overall, only slight transcript variations were detectable within the  most variable expressed genes. (Fig. 5B). Cultivation on GB, WRM, and T112 media caused lower transcript levels of genes coding for terpene synthase TC1, polyketide synthases (PKS7, PKS8), and the NRPS-like1, while an upregulation of NRPS-like2 on WRM (2.5-fold), and of PKS7 (1.7-fold) on reduced 1/3-PDA medium was observed.

Transcript levels of core genes within BGCs assigned to cytochalasines (*cyt*) or xylasporins/cytosporins (*px*), e.g., remained nearly constant, while minor transcript level variations of neighboring genes and reduced transcript levels for *pxI* (flavin-dependent monooxygenase), *pxH* (ABBA-type prenyltransferase), *pxF* (cupin fold oxidoreductase), and *pxJ* (short-chain dehydrogenase) were detectable. Hence, it was concluded that the presence of *Termitomyces* biomass only weakly triggers secondary metabolite production in general, but varying transcript levels coding for decorating enzymes could cause substantial structural alterations within the produced natural product composition. It was also notable that transcript levels of the terpene synthase TC1 were downregulated, which could cause a reduced production level of specific volatiles.

### *Pseudoxylaria* antagonizes *Termitomyces* growth and metabolizes fungal biomass

**Fig. 6:**
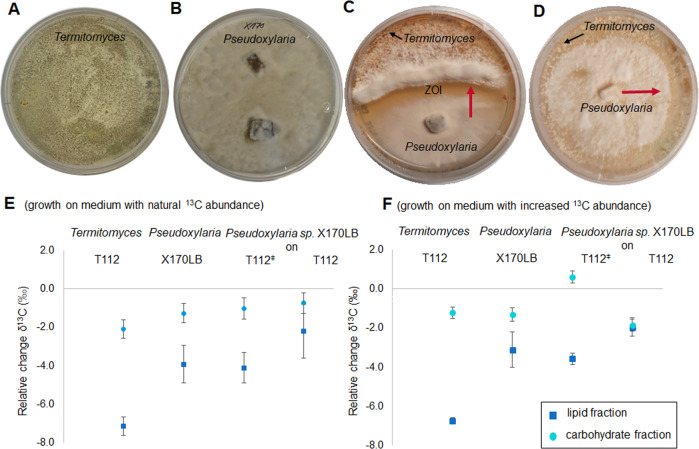
Co-cultivation of *Pseudoxylaria* sp. X170LB and *Termitomyces* sp. T112 and results of isotope fractionation experiments. Representative pictures of fungal growth and co-cultivation of **A** *Termitomyces* sp. T112, **B** *Pseudoxylaria* sp. X170LB, **C** co-culture of *Pseudoxylaria* sp. X802 and *Termitomyces* sp. T153 exhibiting a ZOI, in which X802 overgrowths T153 in proximity to the interaction zone (red arrow), and **D** *Pseudoxylaria* sp. X802 growing on the surface of a living *Termitomyces* sp. T153 culture. **E**, **F** Shown is the relative change in the carbon isotope pattern (δ^13^C values, ± standard deviation, with *n* = 3) of lipid and carbohydrate fractions isolated from fungal biomass of *Termitomyces* sp. T112, *Pseudoxylaria* sp. X170LB, and *Pseudoxylaria* sp. X170LB cultivated on vegetative *Termitomyces* sp. T112 biomass (T112^ǂ^), or on lyophilized *Termitomyces* sp. T112 biomass (T112). Fungal strains were grown on **E** medium with natural ^13^C abundance and **F** medium artificially enriched in ^13^C content.

The growth behavior of *Pseudoxylaria* isolates was also analyzed in co-culture assays with *Termitomyces*. As expected from prior studies, both fungi showed reduced growth when co-cultured on agar plates, often causing the formation of zones of inhibition (ZOI) between the fungal colonies (Fig. 6A–D, Table S11–S14) [7]. When fungus-fungus co-cultures were maintained for longer than two weeks on agar plates, *Pseudoxylaria* started to overcome the ZOI and overgrew *Termitomyces* via the extension of aerial mycelium. The observation was even more pronounced when co-cultures were performed on wood-rice medium (WRM), where *Pseudoxylaria* remained the only visible fungus after two weeks.

To verify whether *Pseudoxylaria* consumes *Termitomyces* or even partially degrades specific metabolites present within the fungal biomass, we pursued stable isotope fingerprinting commonly used to analyse trophic relations [38, 39]. This diagnostic method relies on measurable changes in the bulk stable isotope composition, because biosynthetic enzymes preferentially convert lighter metabolites enriched in ^12^C compared to their heavier ^13^C-enriched congeners. This intrinsic *kinetic isotope effect* results in an overall change in the ^13^C/^12^C ratio of the respective educts and products, in particular in biomarkers such as phospholipid fatty acids, carbohydrates, and amino acids. Using this isotope enrichment effect, we determined the natural trophic isotope fractionation of ^13^C in lipids and carbohydrates produced by *Termitomyces* sp. T112 and *Pseudoxylaria* sp. X170LB. For clearer differentiation, both fungi were cultivated on PDA medium containing naturally abundant ^13^C/^12^C, Fig. 6E) and on PDA medium enriched with ^13^C-glucose (Fig. 6F). Lipids and carbohydrates were isolated from mycelium harvested after 21 days (Fig. 6E, Table S15).

Analysis of fungal carbohydrate and lipid-rich metabolite fractions by Elemental Analysis-Isotope Ratio Mass Spectrometry (EA-IRMS) [40, 41] uncovered that under normal growth conditions (full medium), *Termitomyces* sp. T112 and *Pseudoxylaria* sp. X170LB showed only a slight negative trophic fractionation of stable carbon isotopes (δ^13^C/^12^C ratio (expressed as δ^13^C values [‰]), Fig. 6F) within the carbohydrate fractions (T112: −1.2 ‰; for X170LB: −1.3 ‰), and expectedly a stronger depletion in the lipid fraction (T112: −6.7 ‰, and less pronounced for X170LB: −3.1 ‰). To determine if *Pseudoxylaria* metabolizes *Termitomyces* biomass, the isotope pattern of metabolites derived from *Pseudoxylaria* thriving on living biomass of *Termitomyces* (T112^**ǂ**^) was analysed next. Here, an overall positive carbon isotope (^13^C/^12^C) fractionation by approximately +0.6 ‰ relative to the control medium was detectable, while the δ^13^C values of lipids remained largely unchanged (Fig. 6F, Table S15). These results suggested that *Pseudoxylaria* might pursue a preferential uptake of *Termitomyces*-derived carbohydrates.

In a last experiment, *Pseudoxylaria* was grown on lyophilized (dead) *Termitomyces* biomass (T112) as sole food source. In this experiment, the isotope fingerprint showed converging δ^13^C values of −1.9 ‰ (relative to the media) for both carbohydrate and lipid fractions, which indicated that *Pseudoxylaria* is able to simultaneously metabolize and cycle carbohydrates as well as lipids resulting in the equilibration of isotopic levels between carbohydrates and lipids. Thus, it was concluded that in nature, *Pseudoxylaria* likely harvests nutrients firstly from vegetative *Termitomyces*, and then—if possible—subsequently degrades dying or dead mycelium.

### *Pseudoxylaria* produces antimicrobial secondary metabolites

Based on the observation that *Pseudoxylaria* antagonizes growth of *Termitomyces*, we questioned if the formation of a ZOI might be caused by the secretion of *Pseudoxylaria*-derived antimicrobial metabolites [26, 42]. Thus, we performed an ESI(+)-HRMS/MS based metabolic survey using the web-based platform “Global Natural Product Social Molecular Networking” (GNPS) [43] to correlate the encoded biosynthetic repertoire of *Pseudoxylaria* with secreted metabolites.

**Fig. 7:**
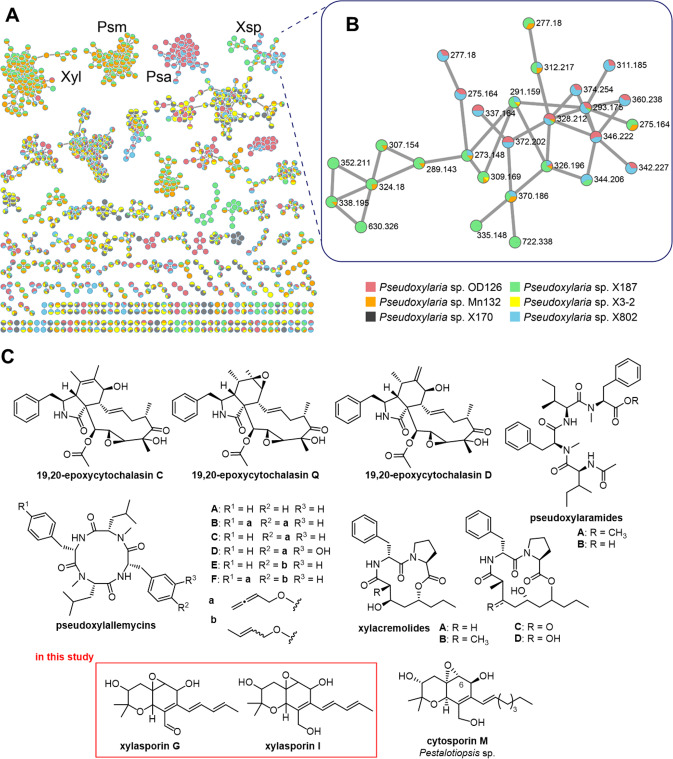
Comparative metabolomic analysis of six *Pseudoxylaria* strains (OD126 (red), Mn132 (orange), X170 (black), X187 (green), X3.2 (yellow) and X802 (blue)). **A** Overview of the GNPS network. Identified metabolite clusters xylacremolides (Xyl; X187/Mn132) [32, 33], pseudoxylaramides [32] (Psa; X187/Mn132), pseudoxylallemycins (Psm; X802/OD126) [18], xylasporin/cytosporins (Xsp; X802/OD126/X187/Mn132) and cytochalasins (X802/OD126) [18]. **B** xylasporin/cytosporin-related cluster formed by nodes from X802 (blue), OD126 (red), X187 (green) and Mn132 (orange). **C** Chemical structures of natural products isolated from *Pseudoxylaria* species and related compounds. Red box highlights proposed structures of isolated xylasporin G and I in this study.

A partial similar metabolic repertoire across the six analyzed strains was detectable and allowed us to match some of the detectable chemical features and previously isolated metabolites to the predicted shared BGCs, such as antifungal and histone deacetylase inhibitory xylacremolides (Xyl; X187/Mn132) [32, 33], pseudoxylaramides (Psa; X187/Mn132) [32], antibacterial pseudoxylallemycins (Psm; X802/OD126) [18], xylasporin/cytosporins (Xsp; X802/OD126/X187/Mn132) [36], and cytotoxic cytochalasins (X802/OD126) (Fig. 7A and B) [18].

A cluster that contained MS^2^ signals of molecular ions assigned to the cytosporin/xylasporin family, which was shared by at least four strains, caught our attention as a certain degree of structural diversity of xylasporin/cytosporin family was predicted from the comparison of their respective BGCs. The assigned nodes of this GNPS cluster split into two subclusters with only very little overlap between both regions. Analysis of the mass fragment shifts suggested that both subclusters belong to two different families of xylasporin/cytosporin congeners (Figure S9). To verify these deductions, we pursued an MS-guided purification of xylasporin/cytosporins from chemical extracts of *Pseudoxylaria* sp. X187, which yielded xylasporin G (3.23 mg, pale-yellow solid) and xylasporin I (1.75 mg, pale-yellow solid). The sum formulas of xylasporin G and xylasporin I were determined to be C_17_H_22_O_5_ (calcd. for [M + H]^+^ C_17_H_23_O_5_^+^ = 307.1540, found 307.15347, −1.726 ppm) and C_17_H_24_O_5_ (calcd. for [M + H]^+^ C_17_H_25_O_5_^+^ = 309.1697, found 309.1691, −1.68 ppm) by ESI-(+)-HRMS and were predicted to have six degrees of unsaturation (Fig. 7B, Figure S10, Table S16-S17). Planar structures were deduced by comparative 1D and 2D NMR analyses, which revealed the presence of an unsaturated polyketide chain that matched the unsaturation degree and the anticipated structural variation from cytosporins (Fig. 7C, Figure S11-S25).

To evaluate if *Pseudoxylaria*-derived culture extracts and produced natural products (e.g., cytochalasins) are responsible for the observed antimicrobial activity, standardized antimicrobial activity assays were performed (Table S17, S18 and Figure S26). As neither culture extracts nor single compounds exhibited significant antimicrobial activity, they could not be held fully accountable for the antagonistic behavior in co-cultures. Thus, we hypothesized that the observed ZOI might be caused by yet unknown effects like nutrient depletion or bioactive enzymes.

### *Pseudoxylaria* has a negative impact on the fitness of insect larvae

**Fig. 8:**
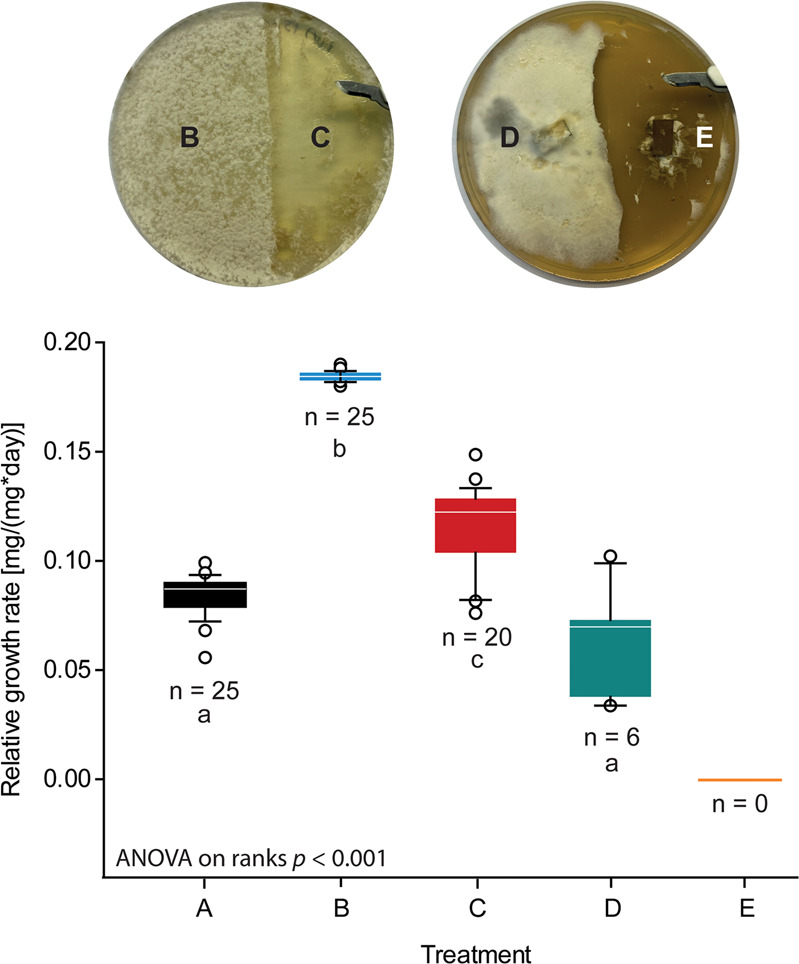
Effect of *Termitomyces* sp. T153 and *Pseudoxylaria* sp. X802 mycelia on the relative growth rate and survival of *S. littoralis* larvae. Insects were fed with either **A** PDA, **B** PDA agar plug covered with vegetative *Termitomyces* sp. T153, **C** PDA agar plug from which vegetative *Termitomyces* sp. T153 was removed prior to feeding, **D** PDA agar plug covered with vegetative *Pseudoxylaria* sp. X802 mycelium, and **E** PDA agar plug from which vegetative *Pseudoxylaria* sp. X802 mycelium was removed prior to feeding. All experiments were performed with 25 replicates per treatment, a duration of 10 days, and larval weights and survival rates were recorded every day. Statistical significances were determined using ANOVA on ranks (*p* < 0.001, *n* = 25) followed by Dunn’s *post-hoc* test (indicated by different letters at the alpha = 0.05 level).

Due to the production of structurally diverse and weakly antimicrobial secondary metabolites, we questioned if mycelium of *Pseudoxylaria* exhibits intrinsic insecticidal or other insect-detrimental activities, which could discourage or ward off grooming behavior of termite workers. Due to the technical challenges associated with behavioral studies of termites, we evaluated instead the effect of *Pseudoxylaria* biomass on *Spodoptera littoralis*, a well-established insect model system and a destructive agricultural lepidopterous pest [44, 45]. When *S. littoralis* larvae were fed with mycelium-covered agar plugs of *Pseudoxylaria* sp. X802, a clear decrease of the relative growth rate (RGR) and decline in survival was observed (Fig. 8: treatment D (green), Table S19, S20) compared to feeding with untreated agar plugs (treatment A (black)). In comparison, when larvae were fed with agar plugs covered with the fungal mutualist *Termitomyces* sp. T153 (treatment B (blue)) an increased growth rate of larvae was observed.

Additionally, *S. littoralis* larvae were also fed agar plugs that had been cleaned from fungal mycelium prior to feeding to test if secreted metabolites and/or depletion of nutrients within the agar matrix might have an impact on RGR and survival. Here, it was surprising to note that agar-plugs derived from *Pseudoxylaria* sp. X802 cultures resulted in the death of all treated caterpillars within six days (treatment E (yellow)). In contrast, feeding with agar plugs previously covered with *Termitomyces* mycelium (treatment C (red)) caused the survival of almost all caterpillars until the end of the experiment, although a slight decline on RGR was observed compared to treatment B (Fig. 8). Thus, an overall beneficial nutritional effect of *Termitomyces* was clearly visible, although a minor negative effect of nutrient depletion within the agar environment during fungal growth could not be fully excluded. Overall, we corroborated from these results that *Pseudoxylaria* exhibits a pronounced negative effect on insect growth and survival, likely due to the combined effect of harmful metabolite secretion, indigestible fungal mycelium and/or nutrient depletion of the growth environment.

## Conclusion

Symbioses of fungi and social insects have independently evolved multiple times in ants, termites, beetles [3], and bees [46]. While genome reduction, and concomitant gene loss are commonly observed alongside with increased specialization and interdependencies in intracellular symbiotic bacteria during their transition to obligate symbiosis, examples of features that define fungal symbiotic interdependencies are sparse [47]. Characterizing features accompanying the evolution of symbiotic fungi is critical to understand symbiotic adaptations and the diversity of life across kingdoms.

Capitalizing on the availability of viable cultures from South African termite colonies, we tested if *Pseudoxylaria* shows features of a termite and comb-associated lifestyle on genomic, transcriptomic and metabolomic levels. In this study, we uncovered genomic evidence for a certain degree of substrate specialization in *Pseudoxylaria* isolates compared to free-living isolates. Similar to termite-associated clades of the fungal genus *Podaxis* [48] and fungal symbiont of attine ants [49], comparative genome analysis revealed reduced sizes and coding capacities, with a reduced enzymatic capacity to oxidatively degrade recalcitrant plant polymers in all *Pseudoxylaria* genomes. Although stochastical losses of biosynthetic traits during evolution cannot be excluded, the depletion in specific traits related to saprophytic life styles has likely been driven by a relaxed selection due to the more benign and constant growth conditions (fungus comb) and the availability of fungus-derived carbohydrate and protein-rich biomass. Based on these findings, and analogous to reports from other obligate fungal symbionts of insects [3, 48], we conclude that *Pseudoxylaria* is likely  an obligate symbiont adapted to the fungus comb environment of farming termites.

While the association of *Pseudoxylaria* with termites might have provided several fitness benefits to the fungus (presence of a carbon/nitrogen-rich comb substrate, protection from UV radiation by the termite mound, presence of ambient temperatures and humidity), termite-associated strains also face biotic stressors within the comb environment, such as termite weeding, co-occurring bacterial communities, and competition from and natural products produced by the fungal cultivar *Termitomyces*.

We hypothesized that *Pseudoxylaria* adapted to such comb-specific stressors by having a reduced but specialized secondary metabolome to reduce triggers that could stimulate alarm responses of the fungal mutualist and termites [50], and the need for specific defense and communication mechanisms to survive. Comparative genome analysis supported the former of these hypotheses as a unique but reduced repertoire of BGCs was present in *Pseudoxylaria* genomes with a notable reduction in TPSs. In contrast, the co-occurring fungal mutualist *Termitomyces* has been found to encode above average numbers of TPSs in previous studies, which correlated with the emission of a bouquet of volatile terpenoid products proposed to play roles in the fungal life cycle by exhibiting insect attractant as well as repellant features [51]. These findings aligned with previous reports on behavioral studies showing that worker castes of *O. obesus* were able to differentiate between their mutualistic crop fungus *Termitomyces* and vegetative mycelium of *Pseudoxylaria* by their volatilome [50].

Our metabolic analysis demonstrated that *Pseudoxylaria* also secretes diffusible bioactive and structurally unique natural products as exemplified by the isolation of two novel metabolites [43]. While neither these  or previously identified metabolites had strong antifungal activity, the production of antimicrobial mixtures could still represent a potential benefit in the competition against the co-occurring microbiota and the fungal mutualists to obtain nutrients.

In nature, *Pseudoxylaria* only emerges from weakened or abandoned comb, where the fungus overgrowths the deteriorating fungus garden. We investigated this phenotypic appearance and documented that *Pseudoxylaria* exhibited not only moderate antagonistic behavior against the termite mutualist *Termitoymces* without instantaneously killing the fungus (reduced antagonism), but showed signs of fungal biomass conversion. The hypothesis that *Pseudoxylaria* might harvest nutrients from vegetative *Termitomyces* mycelium was supported by comparative genome and RNAseq analyses as well as isotope fractionation results.

Overall, this study also provides a good starting point to address several unanswered questions as it still remains puzzling how and when *Pseudoxylaria* enters and remains within the fungus comb, why stromata emerge in the absence of termites (“sit and wait strategy”), and what triggers are required to stimulate germination and growth. This study should also encourage scientists to intensify sampling and sequencing studies on these and other fungal genera to enable broader phylogenomic studies that can address factors driving the evolution of insect-associated fungi in general and termite-associated strains specifically.

## Material and methods

### Cultivation procedures

#### Isolation

Fungal samples were collected from different termite mounds of *Macrotermes natalensis*, *Odontotermes* spp., and *Microtermes* spp. termite species within the years 2015–2018 (Table S1). Comb material was incubated in boxes at room temperature (rt) in the absence of termites, which resulted in the appearance of *Xylaria*-like stromata from fungus comb material. These were aseptically transferred to potato-dextrose agar (PDA) plates and cultivated for several cycles until seven axenic, morphologically distinct and viable cultures were obtained.

### Whole genome sequencing and phylogenetic placement of *Pseudoxylaria*

#### DNA extraction

Mycelium was harvested from agar plates, frozen in liquid nitrogen and grounded to a fine powder. DNA was extracted using CTAB and purified by chloroform-isoamylalcohol (24:1) and subsequent alcohol precipitation for PCR amplification. For direct sequencing the DNeasy Plant kit (Quiagen GmbH, Hilden, Germany) was used. Isolated DNA was kept frozen at −78 °C until sequencing.

#### RNA extraction

*Pseudoxylaria* strain X802 was grown on different media and mycelium of X802 was harvested after 21 days: PD-medium contained only 1/3 PDA per liter (1/3-PDA), PD broth on glass beads (GB), wood-rice medium (WRM), 1/3-PDA containing lyophylized (dead) *Termitomyces* sp. T112 biomass (T112-PDA), and agar-agar (2%) medium containing lyophylized (dead) *Termitomyces* sp. T112 biomass (T112). Mycelium of X802 was kept frozen at −78 °C until RNA extraction for sequencing.

#### Whole genome sequencing

Whole-genome sequencing was performed using a 150 bp paired-end shotgun (BGIseq) and long-read (PacBio sequel) sequencing at BGI. Additionally, Oxford Nanopore technology (Oxford Nanopore Technologies, Oxford, UK) was employed for long-read sequencing. The MinION sequencing library was prepared using the Rapid DNA sequencing kit (SQK-RAD4) according to the manufacturer. DNA sequencing was performed on a MinION Mk1B sequencing device equipped with a R9.4.1 flow cell, which was prepared and run according to the manufacturer.

#### Whole genome assembly

Sequencing results were checked for quality using FastQC version 0.11.8 [52] and MultiQC version 1.7 [53]. Kmer depth was calculated using Jellyfish version 2.2.10 [54] and Kmer-based estimates of genome size, heterozygosity, and repeat content generated using GenomeScope [55]. A hybrid *de novo* genome assembly, combining BGISeq and PacBio data, was performed using SPAdes version 3.13.0 [56]. Nanopore sequencing raw data was generated using MinKNOW software version 4.0.20 (Oxford Nanopore Technologies), and was base-called and trimmed using Guppy version 4.2.2 (Oxford Nanopore Technologies). The resulting fastq files were filtered using Nanofilt [57]. A hybrid *de novo* genome assembly, combining BGISeq, PacBio and Oxford Nanopore data, was performed using MaSuRCA version 3.4.1 [58]. The resulting draft assembly was then polished with the accurate Illumina reads using the POLCA genome polisher (Table S4) [59].

#### RNA-Seq analysis

Sequencing of RNA from was performed by BGISeq (BGI, Hong Kong). Data was mapped against the annotated genome of X802 using Geneious Prime v2021.2.2 (Biomatters Ltd). Normalized transcript counts per million (TPM) and significance of the changes compared to normal growth on PDA were calculated using the built-in function in Geneious. Briefly, the TPM value was calculated using the following formula: TPM = (CDS read count * mean read length * 10^6)/(CDS length * total transcript count). The *p* value was calculated by multiplication of the normalized mean probabilities that a randomly selected transcript would come from a gene (number of transcripts mapped to that gene/total number of transcripts from that sample) of each sample. The resulting data was sorted by variance and imported into R v4.1 (R Foundation) and the TPM and *p* values of most variable genes were log_10_ and log_−10_ transformed, respectively. A heatmap for the identified genes was generated using the pheatmap package v1.0.12 in R with color schemes generated by viridis v0.5.1.

#### Genome annotation

Genomes were annotated using the RNA sequencing reads in BRAKER [60] v2.1.6 creating an Augustus species model for each *Pseudoxylaria* strain. Annotation quality was estimated using BUSCO v5.2.2 with the *sordariomycetales_odb10* dataset [61]. Comparison of predicted gene numbers between clade types was performed in R v4.1.0 (R Core Team, 2020) using either a phylogenetic ANOVA in the package *phytools* v0.7-47 [62] with the ortholog-based phylogenomic tree read in using version 5.4-1 of the *ape* package [63]. Transposable elements were annotated using EDTA v1.9.6 [21].

#### Functional gene annotation

Functional gene annotation was performed using InterProScan version 5.40-77.0 [64] with annotation of Gene Ontology (GO) terms, Panther families and KEGG pathways turned on using the “*goterms*,” “*iprlookup*” and “*pathways*” options respectively. The InterProScan results were then incorporated into the Orthofinder results using Kinfin version 1.0 [65]. Per-orthogroup Gene Ontology (GO) enrichment analyses for each combination of clade types were performed using Pfam domains from InterPro [66] and Kinfin [65] in dcGO [67].

#### Phylogenetic analysis

Genetic loci of interest (internal transcribed spacer (ITS)), second largest subunit of RNA polymerase (RPB2), β-tubulin (TUB), α-actin (ACT) were identified by BLAST [68] search from groups of reference sequences against the fungal genomes. Reference sequences were chosen from the NCBI RefSeq Targeted Loci database [Accession: PRJNA177353] (Table S4). To proof, that correct sequences were obtained by BLAST search, some sequences were doublechecked by PCR amplification and sequencing. If necessary, results from BLAST search and sequencing were combined. Amplification of the target partial gene sequences (fRPB2, ITS, ACT) [13] was done by PCR with S7 Phusion Polymerase (Biozym, Germany): 98 °C 30 s, 35 cycles of 98 °C 30 s, 55–66 °C for 30 s per kb, and 72 °C for 30 s per kb followed by a final denaturation at 98 °C 30 s and extension at 72 °C for 5 min (Table S6). PCR products were cleaned by gel purification (Zymoclean Gel DNA Recovery Kit, Zymo Research, USA) and sequenced at Eurofins Genomics (Ebersberg, Germany). For phylogenetic analysis, sequences from each target loci were aligned with ClustalW, implemented in MEGAX [69]. Phylogenetic trees were prepared with IQ-TREE [70] using ModelFinder [71] and UFBoot [72] with 1000 bootstrap replicates. For phylogenetic analysis, the Galaxy Eu platform was used [73].

#### Mitochondrial genome assembly and annotation

An initial mitochondrial genome assembly was performed using Norgal version 1.0.0 [74] with the best candidate being selected based on a BLAST search. The specimen with the longest circular contig from Norgal was set as a reference and the longest sequence from each strain as a seed in NOVOplasty v4.0 [75]. Mitochondrial genomes were annotated using the MITOS web server [76]. *Pseudoxylaria* mitochondrial genomes were aligned for the visualization of co-linear blocks using Mauve [77] implemented in Geneious Prime (v2021.2.2; Biomatters Ltd.) (Figure S5)

#### Orthology and phylogenomic analyses

Predicted protein sequences from the Augustus annotation were used as inputs to Orthofinder version 2.3.12 [78] using BLAST [79], Mafft [80] version 7.455, FastTree [81] version 2.1.10 and combined into a single species tree using STRIDE [82] and STAG [83]. All phylogenetically-weighted analyses used the phylogeny produced by Orthofinder. The trees were visualized in the *ape* and *dendextend* packages, versions 5.3 [63] and 1.14.0 [84] respectively, in R version 3.6.3 (R Core Team, 2020). Calibration of the phylogenomic tree was performed using the *chronos* function in *ape* and visualized using *phytools* version 0.7–47 [62] in R (R Core Team, 2020) (Figure S2-S4).

#### CAZyme analysis

[85] Functional annotation of carbohydrate-active enzymes was performed using HotPep version 1 with default parameters [86, 87]. CAZymes for which specific EC numbers could be identified were manually annotated with their substrate using the ExPASy database [88] and classified by the substrates presence in plant, bacterial or fungal cell walls. Comparisons of CAZymes and target substrates between clade types were performed using a phylogenetic ANOVA in the package *phytools* version 0.7–47. For the comparison of substrate types, adjusted *p* values were calculated using the false-discovery rate method in the *p.adjust* function (Figure S6).

#### In silico analysis of the biosynthetic gene clusters

To identify the responsible biosynthetic gene clusters (BGCs) the *Pseudoxylaria* and *Xylaria* genomes were annotated using the fungal version of antiSMASH 6.0.0 [29]. In case of incomplete or fragmented gene cluster hits, sequences were manually reanalyzed using BLAST. Anticipating that in genomes of ascomycetes fungi core genes of a biosynthetic pathway are co-localized within a certain gene region (< 150 kbp), we counted those hits, which showed sequence homologies higher than 50% to other known sequences and co-localized with sequences relating to the same metabolic pathway. Gen models for *Xylaria* genomes were predicted with Augustus using the created gene model of *Pseudoxylaria* sp. X802 [89]. BiG-SCAPE 1.0 was used to create the similarity networks [30]. Two cutoff values were used: 0.3 (default) and 0.5. Other parameters used: *--include_singletons* --*mix --mibig*. Networks were visualized with Cytoscape (v3.8.1; https://cytoscape.org/) and manually curated. Homology searches were performed with cblaster v1.3.11 [90] and homologous gene clusters were visualized in Geneious Prime (v2021.2.3; Biomatters Ltd.) (Figure S8).

### Growth studies

#### PDA medium

*Pseudoxylaria* strains were cultivated on PDA plates for a maximum of four weeks at 25 °C and sub-cultured by plating mycelium-containing agar pieces (0.5 × 0.5 cm) onto PDA. For inoculation of *Termitomyces* strains, vegetative biomass was scraped from agar plates and suspended in Dulbecco’s phosphate buffered saline (PBS, 10 mL/plate).

#### Wood medium

Wood medium was prepared by soaking and swelling sawdust for 20 min in warm water, which were then autoclaved twice (24 h apart) in 50 mL glass beaker covered with aluminum containing sawdust (25 g).

#### Wood-rice medium

A 1:1 mixture of boiled rice and swelled sawdust were kept moist, filled into 100 mL glass beakers (50 mL each), sealed with aluminum foil, and sterilized twice 24 h apart (121 °C, 20 min). For *Termitomyces* cultures, medium was inoculated with a mycelial suspension (1.5 mL). For *Pseudoxylaria* cultures, agar pieces containing vegetative mycelium were placed on the wood-rice surface. Cultures incubated for up to four weeks at rt and monitored daily. Sterile wood-rice medium was kept as control.

#### Fungus comb medium

Fungus comb material (5.0 g per beaker including sterile wet tissue paper) was autoclaved twice, 24 h apart. Beakers were individually inoculated with *Termitomyces* sp. T153 and T112, as well as *Pseudoxylaria* strain X802 and X170LB. Sterile fungus-comb medium was kept as control.

#### Fungus-fungus co-culture

Fungal cultures (*Pseudoxylaria* strains OD126, X802, X187, Mn132, X3-2, X170LB, *Termitomyces* sp.) were cultivated on standard PDA plates (92 mm × 16 mm) for two weeks at 25 °C and used for co-culture set-ups. **Method A**: *Termitomyces* was inoculated as a mycelium suspension on a PDA plate and cultivated for eight days; then *Pseudoxylaria* was inoculated next to (or on top) of the *Termitomyces* culture. **Method B:** Agar plugs covered with *Pseudoxylaria* mycelium were added directly onto agar plates freshly inoculated with *Termitomyces*. **Method C:** *Termitomyces* was pre-grown on wood-rice medium (17 days) and then inoculated next to agar plugs of a *Pseudoxylaria* culture. All cocultures were incubated at rt for up to 4 weeks and monitored on a daily basis.

### Metabolic δ^13^C/^12^C isotope fractionation analysis

#### Cultivation Method A

Fungal strains (*Termitomyces* sp. T112, *Pseudoxylaria* sp. X170LB) were cultured as single strains and fungal co-cultures on both, regular and ^13^C glucose-enriched PDA medium. The δ^13^C of glucose in the enriched medium was artificially adjusted to +40‰.

#### Cultivation Method B

*Pseudoxylaria* sp. X170LB was cultivated on an agar-agar matrix containing natural or isotope-enriched lyophilized *Termitomyces* sp. T112 biomass (~70 mg biomass, 2% agar-agar, 10 mL), which was obtained after cultivation on either regular or ^13^C-enriched PDA (14 d, 25 °C). For inoculation, a single agar plug (0.3 cm × 0.3 cm) of *Pseudoxylaria* sp. X170LB was inoculated on top of the biomass containing plate, and incubated for 21 days at rt. Inoculated plates containing only 2% agar-agar (20 mL) served as control. Samples were derived by homogenization starting with six biological replicates (*N* = 6). Two plates each were then combined during sample collection resulting in triplicates for extraction and analysis (*N* = 3).

#### Sample analysis

*Pseudoxylaria* sp. X170LB mycelium was carefully separated from the cultivation matrix (either agar surface or from the top of the *Termitomyces* mycelium) and transferred into sterile, cauterized (500 °C, 5 h) glass vials. Samples were dried by lyophilization and weighed. *Termitomyces* sp. T112 mycelium for comparative controls was collected in a similar manner. Dry fungal biomass was homogenized in a mortar and transferred into glass centrifuge tubes. The resulting powder was subsequently mixed with 20 mL MeOH/H_2_O/DCM (25%/25%/50%), sonicated (30 °C, 10 min) and centrifuged (5 min, 2000 rpm). The unpolar, lipid containing lower layer was removed with a glass pipette and collected in a separate glass tube. The extraction was repeated twice via addition of 5 mL fresh DCM to the remaining polar MeOH fraction, and combined extracts were dried over Na_2_SO_4_. The final sugar containing MeOH fraction was collected separately. Samples were concentrated to 0.5 mL in a N_2_ stream and *in vacuo* (30–40 °C) and stored at −20 °C.

#### Solid phase extraction and analysis

The unpolar lipid fraction was separated by silica column chromatography (SiO_2_). Neutral lipids were eluted with two column volumes DCM, followed by glycolipids with two column volumes acetone and phospholipids with four column volumes MeOH. Only the phospholipid containing fraction was further analyzed in this study. Samples (0.01 mg–2.45 mg) were transferred to tin capsules, dried (40 °C, 1 h) and submitted for elemental analysis and δ^13^C/δ^12^C measurements. Elemental analysis and carbon isotope ratio analysis was performed using an elemental analysis isotope ratio mass spectrometer (EA-IRMS) fitted with an elemental analysator A (NA 1110, CE Instruments, Mailand) coupled with a ConFlo III and a Delta XL-IRMS (Thermo-Finnigan, Bremen). Acetylanilid (Ali-j3) and caffeine (caf-j3) were used as analytical standards (Table S15).

### Metabolomic analysis

#### Cultivation and analysis

Fungal isolates were cultivated on standard PDA plates (92 mm × 16 mm) for two weeks at 25 °C. Mycelium covered agar was cut into small pieces (0.5 cm × 0.5 cm) and extracted with EtOAc overnight (50 mL/plate). Solvent was removed to dryness under reduced pressure. Raw extracts were dissolved in MeOH (50 µg/mL) and submitted for ESI(+)-HRMS/MS analysis on a Dionex Ultimate3000 system (Thermo Scientific) combined with a Q-Exactive Plus mass spectrometer (Thermo Scientific) and an electrospray ion (ESI) source. Metabolite separation was carried out by reverse phase liquid chromatography at 40 °C using a Luna Omega C18 column (100 × 2.1 mm, particle size 1.6 μm, 100 Å, Phenomenex) preceded by a SecurityGuard ULTRA guard cartridge (2 × 2.1 mm, Phenomenex). Mobile phases were acidified with 0.1% formic acid and consisted of H_2_O (A) and acetonitrile (B). In total, 5 μl of each sample were injected and metabolite separation was achieved using the following gradient: 0–0.8 min, 5% B; 10 min, 97% B; 10–12 min, 97% B; 13 min, 5% B; 13–15 min, 5% B at a constant flow rate of 0.3 mL/min. Metabolites were detected in positive (centroid) ionization mode within a range of *m*/*z* 180–1800 with a resolving power of 70,000 at *m*/*z* 200. MS^2^ measurements were performed by combination of data-dependent MS^2^ analysis and Top10 experiments.

#### Molecular network by GNPS

MS/MS-data was filtered by removing all MS/MS peaks within +/− 17 Da of the precursor *m/z*. MS/MS spectra were window filtered by choosing only the top 6 peaks in the +/− 50 Da window throughout the spectrum. The data was then clustered with MS-Cluster with a parent mass tolerance of 0.02 Da and a MS/MS fragment ion tolerance of 0.1 Da to create consensus spectra. Further, consensus spectra that contained less than 2 spectra were discarded. A network was then created where edges were filtered to have a cosine score above 0.9 and more than 6 matched peaks. Further edges between two nodes were kept in the network if and only if each of the nodes appeared in each other’s respective top 10 most similar nodes. The spectra in the network were then searched against GNPS’ spectral libraries. All matches kept between network spectra and library spectra were required to have a score above 0.7 and at least 6 matched peaks.

#### Purification and structure elucidation of xylasporin G and I

Raw extracts (54 mg) obtained from EtOAc extraction of *Pseudoxylaria* sp. X187 (20 plates PDA, static cultivation, 14 d, room temperature) were purified by semi-preparative HPLC on a Phenomenex Luna C18 100 Å LC Column 250 × 10 mm (particle size 5 μm, pore diameter 100 Å) using a gradient of (**A**) acetonitrile and (**B**) H_2_O + 0.1% formic acid. Xylasporin G (3.23 mg) was obtained as a yellow solid, and xylasporin I (1.75 mg) was obtained as a pale-yellow solid, which decomposed within hours when exposed to air and traces of acid. NMR measurements were performed on a Bruker AVANCE III 600 MHz spectrometer, equipped with a Bruker Cryoplatform. The chemical shifts are reported in parts per million (ppm) relative to the solvent residual peak deuterated solvent.

### Bioactivity studies

#### Testing of extracts obtained from *Pseudoxylaria* on fungal growth of *Termitomyces*

*Pseudoxylaria* strains (X187, Mn132, X802, OD126, X3.2, X170LB) were cultivated on PDA plates at 25 °C for up to 14 days. Two mycelium covered agar plates were cut into pieces and extracted with EtOAc or MeOH (100 mL per plate), respectively. Concentrated EtOAc extracts were re-dissolved in MeOH (5 mg/mL) and used in disc-diffusion tests (30 µL = 150 µg extract, Whatman antibiotic assay discs, 6 mm (GE Healthcare)) against *Termitomyces* sp. T153. Methanolic extracts were concentrated under reduced pressure and crude as well as solid-phase extracts eluted from SPE column using MeOH/H_2_O mixtures.

#### Standard disc-diffusion antimicrobial activity assays

Culture extracts obtained from agar plate cultivations and commercial cytochalasin D and B (Sigma Aldrich) were tested in disc-diffusion tests. Culture extracts (1 mg/mL) in MeOH were tested for their ability to inhibit growth of the following indicator strains: *Bacillus subtilis* 6633; *Staphylococcus aureus* SG511, *Escherichia coli* SG458, *Pseudomonas aeruginosa* K799/61, *Mycobacterium vaccae* 10670, *Sporobolomyces salmonicolor* 549, *Candida albicans* C.A., *Penicillium notatum* JP36. Antimicrobial activity was determined by measuring the inhibition zone in mm (Table S17 and S18). Cytochalasins (75 µg cytochalasin in MeOH) and culture extracts (1 mg/mL in MeOH) were also tested for their ability to inhibit growth of *Termitomyces* for up to 3 weeks at 25 °C. Antimicrobial activity was determined by measuring the inhibition zone in mm (Table S17 and S18).

#### Broth-dilution assay of *Pseudoxylaria* sp. extracts against *Saccharomyces cerevisiae*

Yeast strain BY4741 (uracil deficient) was grown in SD medium (yeast synthetic defined medium) amended with uracil (20 mg/L). Assay was performed in 96 well plates with n = 6. First, 4 µL extract (MeOH, 5 mg/mL) were added to each well and dried, then 200 µL fresh yeast culture (OD = 0.02) were added per well (inoculated at 2%) and optical density measured every hour for 24 h at 30 °C under shaking.

### Insect antifeedant assay

#### Cultivation

A 14-day old pre-culture of *Pseudoxylaria* sp. X802 (200 mL PDB, 14 days at 30 °C and 150 rpm) was used to inoculate 20 PDA plates (92 × 16 mm, 2 mL inoculum each). For *Termitomyces* sp. T153, 20 PDA petri dishes (92 × 16 mm) were inoculated with 0.5 mL of a mycelium suspension (10 mL PBS, mycelium of 14-day old *Termitomyces* sp. T153 culture). Fungal strains were incubated for 14 days at 30 °C. Untreated PDA plates (10) served as medium control.

#### Organisms and artificial diet

Larvae of *S. littoralis* Boisduval (Egyptian cotton leafworm) were hatched and reared on a modified artificial bean diet [45]. Larvae were kept at 19 °C and under a light cycle of 12:12 h. Larvae in the 3rd larval stage (L3) were used for all experiments.

#### Antifeedant insect performance assays

In all feeding assays, freshly molted L3 larvae (5–10 mg) were placed into polystyrene cups (Solo 3.5 oz) containing an agar piece from a 2-week-old fungus culture on PDA, punched out with a sterile 5 mL pipette tip (~ 1 cm^2^), and allowed to feed *ad libitum*. Larvae were assigned randomly to a feeding treatment and during the experiment, kept under controlled conditions and fresh diet was provided daily. All experiments were performed with 25 replicates per treatment, a duration of 10 days, and larval weights and survival rates were recorded every day. The relative growth rate (RGR) was calculated for each caterpillar that survived until the end of the experiment [45]. Feeding experiments were repeated independently two times for each fungus.

#### Treatments

The insects were fed with PDA (A), *Termitomyces* sp. T153 (B) or *Pseudoxylaria* sp. X802 (D) growing on PDA and PDA after removing the fungal mycelium of the latter fungi, respectively (C, E). The RGR was calculated for each caterpillar that survived until the end of the experiment and is expressed in mg/(mg * days) [45]. Listed are the average RGR values ± standard errors. Calculations were not performed for those treatments where no insects were left in the end of the experiment (N/A). Statistical significances were determined using ANOVA, followed by a suitable *post-hoc* test depending on the pre-requisites of the data set. Two additional replicates each were performed for *Termitomyces* sp. T153 and *Pseudoxylaria* sp. X802 (see Table S19), where insects were fed with PDA (A), fungus growing on PDA (B) or PDA after removing the fungal mycelium (C) in a separate experimental setup for each fungus.

#### Statistical analysis

Statistical analysis was performed using SigmaPlot 12.0 (Systat Software, Inc., San Jose, USA). Data was checked for statistical pre-requisites such as homogeneity of variances and normality. Depending on the data type and its match of pre-requisites a respective statistical test was chosen. The chosen statistical test and its results are mentioned in the subtitles of each table or figure.

## Supplementary information

Supporting Information

## Data Availability

Supporting Information of this article is free of charge and contains list of accession numbers of sequences used for analysis, phylogenetic trees, cultivation studies including co-cultivation, analyses of genomic and metabolomic data, NMR and MS-data of isolated metabolites and data of insect feeding studies including statistical analyses.
