# Supplementary material for: Adaptations of Pseudoxylaria towards a comb-associated lifestyle in fungus-farming termite colonies
Source: ISME J. 2023 Feb 25;17(5):733–47. doi: 10.1038/s41396-023-01374-4 (PMC10119272; doi:10.1038/s41396-023-01374-4)
Supplement: Supplementary file 1 — Supporting Information [file 41396_2023_1374_MOESM1_ESM.pdf]

Supporting Information

Adaptations of *Pseudoxylaria* towards a comb-associated lifestyle in fungus-farming termite colonies

Janis Fricke,[#1] Felix Schalk,[#1] Nina B. Kreuzenbeck,[1] Elena Seibel,[1] Judith Hoffmann,[1] Georg Dittmann,[2] Benjamin H. Conlon,[3] Huijuan Guo,[1] Z. Wilhelm de Beer,[4] Daniel Giddings Vassão,[5] Gerd Gleixner,[2] Michael Poulsen,[3] Christine Beemelmanns[1,6,7]*

1. Group Chemical Biology of Microbe-Host Interactions, Leibniz Institute for Natural Product Research and Infection Biology – Hans Knöll Institute (HKI), Beutenbergstraße 11a, 07745 Jena, Germany,
2. Department of Biogeochemical Processes, Max Planck Institute for Biogeochemistry, Hans-Knöll-Straße 10, 07745 Jena
3. Department of Biology, Section for Ecology and Evolution, University of Copenhagen, Universitetsparken 15, 2100 Copenhagen, Denmark
4. Department of Biochemistry, Genetics and Microbiology, Forestry and Agricultural Biotechnology Institute (FABI), University of Pretoria, Hatfield 0028, Pretoria, South Africa
5. Department of Biochemistry, Max Planck Institute for Chemical Ecology, Hans-Knöll-Straße 8, 07745 Jena
6. Department Anti-infectives from Microbiota, Helmholtz Institute for Pharmaceutical Resea rch Saarland (HIPS), Helmholtz Centre for Infection Research, Saarland University Camp us, 66123 Saarbrücken, Germany
7. Saarland University, 66123 Saarbrücken, Germany

[#] contributed equally; alphabetical order

*Corresponding author: Christine Beemelmanns,

E-mail: Christine.Beemelmanns@helmholtz-hips.de

List of Figures

Figure S1. Maximum-likelihood ITS tree of *Xylariales* fungi calculated with 1000 bootstrap replicates. For tree generation, a symmetric model with unequal rates but equal base frequencies were used. The model of rate heterogeneity is invariant using discrete gamma model with 4 categories. The tree is unrooted. 13

Figure S2. Maximum likelihood tree of *Xylaria* sp. using ACT as phylogenetic marker, calculated with 1000 bootstrap replicates. Clades are assigned according to Hsieh et al 2005.Phylogeny was predicted using TIM2 model as base substitution rates with empirical base frequencies. The model of rate heterogeneity is invariant using discrete gamma model with 4 categories. The tree is unrooted. 14

Figure S3. Maximum likelihood tree of *Xylaria* sp. using RPB2 as phylogenetic marker, calculated with 1000 bootstrap replicates. Clades are assigned according to Hsieh et al 2005. RPB2 gene tree was calculated using general time reversible model with unequal rates and empirical unequal base frequencies. For rate heterogeneity, a free rate model with 5 categories was used. 15

Figure S4. Maximum likelihood tree of *Xylaria* sp. using TUB as phylogenetic marker, calculated with 1000 bootstrap replicates. Clades are assigned according to Hsieh et al 2005. Phylogeny was predicted using TIM2 model as base substitution rates with empirical, unequal base frequency. For rate heterogeneity, a free rate model with 16 categories was used. 16

Figure S5. Mauve alignment of the *Pseudoxylaria* mitochondrial genome assemblies. For the assembly the software Mauve was used. Locally collinear blocks are highlighted in the same color. 17

Figure S6. Comparative CAZY analysis of free-living *Xylaria*, termite-associated *Pseudoxylaria*-clade and *Termitomyces*. 19

Figure S7. A) Numbers of BGCs classes identified in the genomes of *Pseudoxylaria* and free-living *Xylaria* species. B) Biosynthetic gene cluster analysis of *Pseudoxylaria* isolates. A) Heatmap depicting the numbers of identified BGCs classes annotated as polyketide synthases (PKS), non-ribosomal peptide synthases (NRPS), polyketide non-ribosomal peptide hybrid synthases (PKS-NRPS), NRPS-like synthases (with a domain architecture of A-T-TE and A-T-R), terpene synthases, ribosomally-synthesized and post-translationally modified peptides (RiPP), and halogenases. Shown on the bottom is the ratio of mean BGC numbers per group from not-significant (green) to significant (***red). 21

Figure S8. BGCs encoding for the productions of cytochalasan, xylasporin/cytosporin, and xylacremolide (polyketide synthases (PKS), non-ribosomal peptide synthetases (NRPS), PKS-NRPS hybrids, short-chain reductases (SDR), cupin-fold oxidoreduxtases (cupin), monooxygenases (MO), Diels-Alderases, SnoaL-like cyclases (cyclase), aromatic ABBA-type prenyltransferases (PT), α-β-hydrolases (hydrolase), acyl transferases (transferase), transcription factors (TF), and major facilitator type transporters (MFS). Red circled numbers indicate the different strains: (1) *Pseudoxylaria* spp. Mn132, (2) Mn153, (5) X187, (7) X802, (8) *Xylaria* spp. BCC 1067, (10) MSU_SB201401, (11) *X. flabeliformis* G536, (12) *X. grammica* EL000614, and (15) *X. hypoxylon* DSM 108379.Identified homologous biosynthetic genetic loci of the cytosporin/xylasporin (*px*) gene cluster. B) Heatmap showing the abundance and identity in % (white to dark blue) of co-localized homologous *px* genes in other ascomycete genomes deposited in the NCBI database was generated using cblaster v1.3.11. 22

Figure S9. GNPS network of EtOAc extracts from six *Pseudoxylaria* spp. grown on PDA for two weeks. Colored nodes represent molecular ions from extracts of: Pseudoxylaria sp. OD126 (red), Pseudoxylaria sp. X802 (blue), *Pseudoxylaria* sp. X187 (green), *Pseudoxylaria* sp. Mn132 (orange), *Pseudoxylaria* sp. X3.2 (yellow) and *Pseudoxylaria* sp. X170LB (black). Identified metabolite clusters are represent as A) xylacremolides (X187/Mn132), B) pseudoxylariamides (X187/Mn132), C) pseudoxylallemcycins (X802/OD126), D) cytosporin/xylasporins (X802/OD126 and X187/Mn132) and E) cytochalasins (X802/OD126). 32

Figure S10. LC-HRMS chromatogram of *Pseudoxylaria* sp. X187 raw extract (EtOAc) with chromatogram traces A) TIC and XICs corresponding to novel compounds: B) xylasporin G, *m/z* 309.1691; C) xylasporin I, *m/z* 307.1535 and D) xylasporin H, *m/z* 335.1484. 33

Figure S11. Semipreparative HPLC chromatogram of culture extracts showing fractions of xylasporin I (Fraction 0) and xylasporin G (Fraction 4). Gradient of ACN and H_2O+ 0.1% FA is displayed in light blue. 33

Figure S12. Integrated ^{1}H-NMR spectrum of xylasporin G in $CDCl_3$, 500 Mhz. 36

Figure S13. Peak-picked ^{13}C-NMR spectrum of xylasporin G in $CDCl_3$, 500 Mhz. 37

Figure S14. ^{13}C-NMR spectrum of xylasporin G in MeOH-*d3*, 500 Mhz. 38

Figure S15. Peak-picked DEPT135 spectrum of xylasporin G in $CDCl_3$, 500 Mhz. 39

Figure S16. HSQC spectrum of xylasporin G in $CDCl_3$, 500 Mhz. 40

Figure S17. HMBC spectrum of xylasporin G in $CDCl_3$, 500 Mhz. 41

Figure S18. COSY spectrum of xylasporin G in $CDCl_3$, 500 Mhz. 42

Figure S19. ^{1}H-NMR of xylasporin I in $CDCl_3$, 500 Mhz. 43
Figure S20. Comparison of ^{1}H NMR spectra ($CDCl_3$, 500 Mhz) for xylasporin G (top) and xylasporin I (inverted, bottom). Spectra are inverted based on the ppm axis and are displayed from 0 to 4 ppm. 44
Figure S21. Comparison of ^{1}H NMR spectra ($CDCl_3$, 500 Mhz) for xylasporin G (top) and xylasporin I (inverted, bottom). Spectra are inverted based on the ppm axis and are displayed from 3.8 to 5.1 ppm. 45
Figure S22. Comparison of ^{1}H NMR spectra for xylasporin G (top side) and xylasporin I (inverted, bottom). Spectra are inverted based on the ppm axis and are displayed from 5.5 to 10.5 ppm. $CDCl_3$, 500 Mhz. 46
Figure S23. ESI(+)-HRMS spectrum of xylasporin G. A strong double water loss and adduct formation, *m/z* $[M+H]^+$ = 307.15347, $C_{17}H_{23}O_5^+$ calcd. -1.726 ppm. RT = 6.48 min was observed. 47
Figure S24. ESI(+)-HRMS spectrum of xylasporin I. A strong double water loss and adduct formation, *m/z* $[M+H]^+$ = 309.16910, $C_{17}H_{25}O_5^+$ calcd. -1.683 ppm. RT = 5.53 min was observed. 48
Figure S25. ESI(+)-HRMS spectrum of hypothesized xylasporin H carbonate (*m/z* $[M+H]^+$ = 335.1484, $C_{18}H_{23}O_6^+$ calcd. 1.417 ppm. RT = 6.70 min). 49
Figure S26. Broth dilution assay of *Pseudoxylaria* sp. extracts against *Saccharomyces cerevisiae* BY4741..... 52

List of Tables

Table S1. Geographic locations of termite colonies used for isolation of *Pseudoxylaria* strains..7
Table S2. Media Composition. 7
Table S3. Growth of *Pseudoxylaria* strains on PDA medium at RT and at different time points. Top-down view and bottom up view...8
Table S4. Sequencing, assembly and annotation statistics of genomes from for *Pseudoxylaria* spp. and *Xylaria* spp. included in this study (I: BGIseq, IT: Illumina, PB: PacBio, ONT: Oxford Nanopore Technologies; *** g**enome was excluded from the study due to low quality 10
Table S5. *Xylaria* ITS reference sequences from NCBI fungal ITS RefSeq targeted loci database (marked in grey) and isolated *Pseudoxylaria* strains (upper part). Gene accession numbers of ITS sequences used for phylogenetic analysis as reference data was selected from Hsieh et al 2005 to provide a framework for phylogenetic classification (lower part)....................11
Table S6. Gene accession numbers of partial protein-coding genes ACT, RPB2 and TUB used for phylogenetic analysis and identified sequences from isolated *Xylaria* strains (lower part). 12
Table S7. Primers and PCR conditions for amplification of fungal phylogenetic marker sequences...13
Table S8. Mitochondrial assemblies and annotation statistics of species from the Xylariales used in this study. 17
Table S9. Identified transposable elements (TEs) in *Pseudoxylaria* and *Xylaria* species using EDTA. 18
Table S10. Additional identified redox active enzymes in *Pseudoxylaria* and *Xylaria* species. ..20
Table S11. Schematic representation of co-cultivation set-ups to determine inhibition of growth: Method **A)** Simultaneous co-cultivation of *Termitomyces* sp. T153 and *Pseudoxylaria* sp. (black square) after 2 or 3 weeks (W), and Method B) *Pseudoxylaria* sp. (black circle); was first incubated on a PDA plate and then challenged with *Termitomyces* sp. T153. 23
Table S12. Pictures of co-culture studies and axenic controls of *Termitomyces* sp. T153 and *Pseudoxylaria* sp. X170LB, X802 on PDA media after 6, 12 and 35 days. Inoculation of *Pseudoxylaria* (black square) was performed A) on top of a vegetative *Termitomyces* culture (grey color), B) next to a vegetative *Termitomyces* culture or C) both fungi were inoculated simultaneously. 25
Table S13. Pictures of *Pseudoxylaria* sp. X170LB and X802 cultures on water-agarose medium and water-agarose medium supplemented with lyophilized *Termitomyces* sp. T112 biomass or 1/3 PDA...27

Table S14. Pictures of fungal co-cultures of different *Termitomyces* (T112, T153, P5HKI) and *Pseudoxylaria* strains (X802, X170LB) on wood-rice medium (WRM). 28
Table S15. Results for stable carbon isotopic fractionation experiments for sugars and lipids performed with *Termitomyces* sp. T112, *Pseudoxylaria* sp. X170LB in axenic cultures and cocultures. 31
Table S16. NMR assignment of xylasporin G and I based on HMBC and key ^1H-^1H COSY correlations ($CDCl_3$), 500 MHz. 34
Table S17. Antimicrobial activity of *Pseudoxylaria* sp. extracts was determined by measuring the inhibition zone (ZOI) in mm ((p) colonies inside inhibition zone, (P) many colonies inside inhibition zone, (A) visible hint indicating potential inhibition) 50
Table S18. Disc diffusion assay against *Termitoymces* sp. 153 using cytochalasines and *Pseudoxylaria* sp. extracts. 51
Table S19. Feeding studies investigating the effect of fungus consumption on the relative growth rate (RGR) of *S. littoralis* caterpillars. Insects were fed with PDA (A), *Termitomyces* sp. T153 (B) or *Pseudoxylaria* sp. X802 (D) growing on PDA and PDA after removing fungal mycelium of the latter fungi, respectively (C, E). 53
Table S20. Results of additional insect feeding studies. 53

Table S1. Geographic locations of termite colonies used for isolation of *Pseudoxylaria* strains.

Strain	Termite species	Excavation site	Isolation date
Mn132	*Macrotermes natalensis*	S24 40.484 E28 48.271	2016
Mn153	*Macrotermes natalensis*	S25 44.492 E28 15.663	2016
X167	*Odontotermes* sp.	S25 43.777 E28 14.423	2016
X170	*Odontotermes* sp.	S25 56.636 E30 35.833	2016
X3-2	*Macrotermes natalensis*	S26 50.163 E30 30.490	2016
X187	*Macrotermes natalensis*	S24 40.434 E28 48.275	2018
X802	*Microtermes* sp.	S25 43 55.7 E28 14 08.2	2008
OD126	*Microtermes* sp.	S25 43 55.7 E28 14 08.2	2008

Table S2. Media Composition.

Medium	Compound	Concentration	Vendor
**PDA (Potato extract dextrose agar)**	Potato extract	6,5 g/l	Carl Roth
	Dextrose	20 g/l	Carl Roth
	Agarose	20 g/l	
**1/3 PDA (diluted PDA)**	Potato extract	2,2 g/l	Carl Roth
	Dextrose	6,7 g/l	Carl Roth
	Agarose	20 g/l	
**Agar-agar**	dd H_2O		
	Agarose	20 g/l	
**Rice wood**	Sawdust	50 Vol.-%	Perfecto Nager
	Rice	50 Vol.-%	Alnatura
**Wood**	Sawdust	100 Vol.-%	Perfecto Nager
**Fungus comb**	Fungus comb	100 Vol.-%	
**Wood-Fungus comb**	Sawdust	50 Vol.-%	Perfecto Nager
	Fungus comb	50 Vol.-%	
**PDA-*Termitomyces* (1/3 PDA-T112)**	Potato extract	2,2 g/l	Carl Roth
	Dextrose	6,7 g/l	Carl Roth
	Termitomyces (dry)	~ 5 g/l	
***Termitomyces* (T112)**	dd H_2O		
	Termitomyces (dry)	~ 5 g/l	

Table S3. Growth of *Pseudoxylaria* strains on PDA medium at RT and at different time points. Top-down view and bottom up view.

**Strain**				
**X802**	5 d - Top	5 d - bottom	21 d - Top	21 d- bottom
	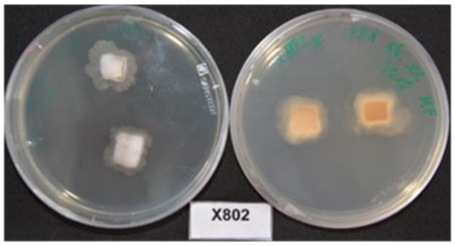		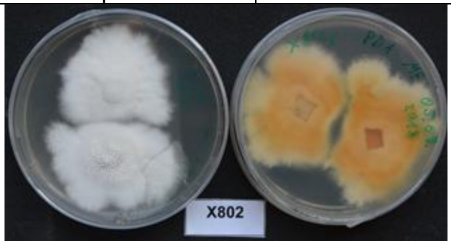	
**OD126**	3 d - top	3 d - bottom	21 d - top	21 d – bottom
	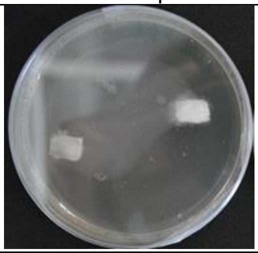	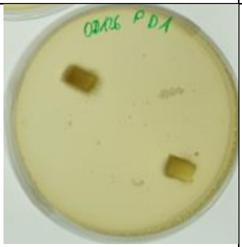	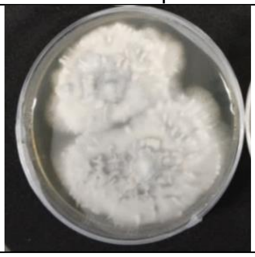	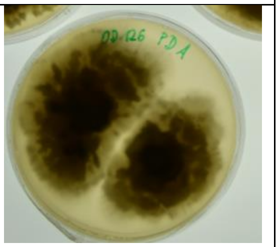
**X187-2**	3 d – top	3 d - bottom	10 d - top	30 d - bottom
	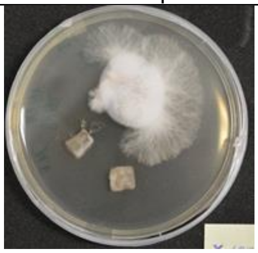	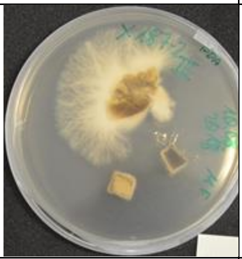	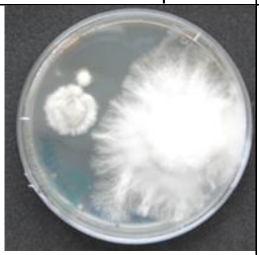	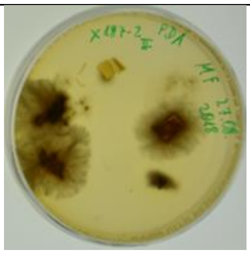
**X3-2**	5 d - top	5 d - bottom	17 d – top	17 d – bottom
	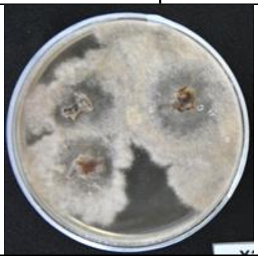	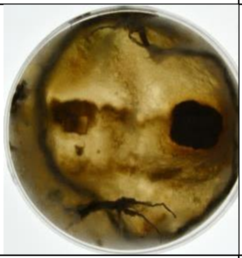	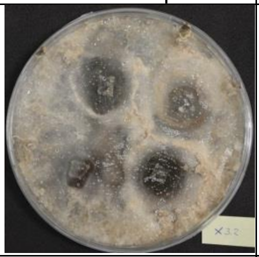	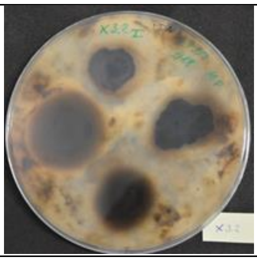
**X167**	7 d - top	3 d - bottom	21 d – top	21 d - bottom
	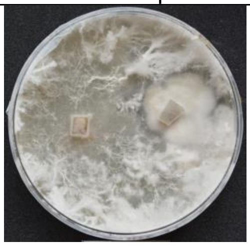	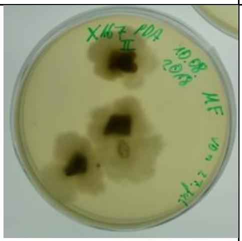	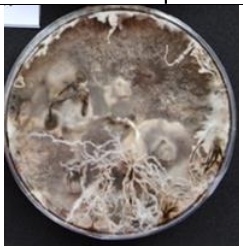	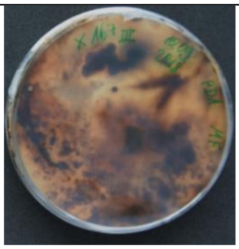

**X170 LB**	4 d – top	4 d – bottom	17 d – top	21 d - bottom
	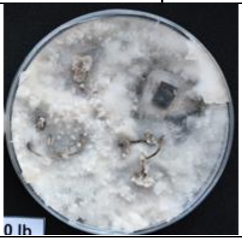	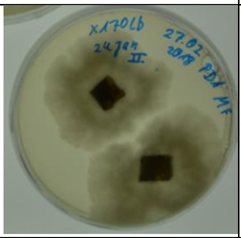	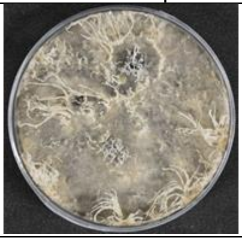	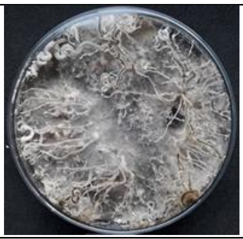
**MN132H12**	3 d - top	6 d – bottom	12-d - top	14 d - bottom
	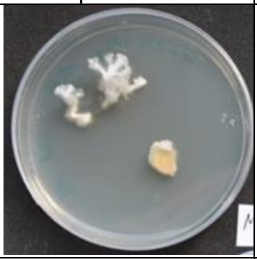	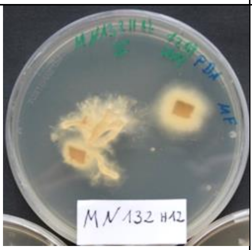	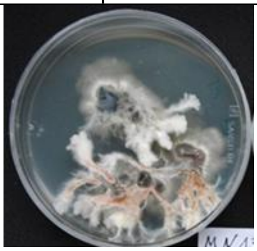	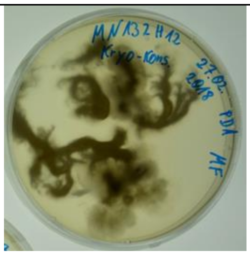
**MN153 H3**	10 d – top	6 d – bottom	15 d – top	15 d - bottom
	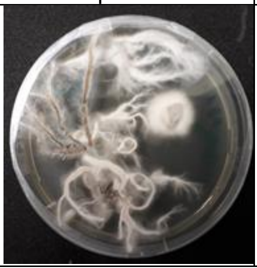	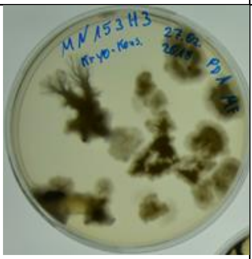	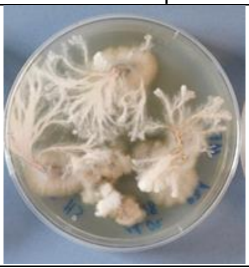	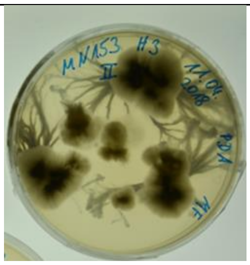

Table S4. Sequencing, assembly and annotation statistics of genomes from for *Pseudoxylaria* spp. and *Xylaria* spp. included in this study (I: BGIseq, IT: Illumina, PB: PacBio, ONT: Oxford Nanopore Technologies; *** g**enome was excluded from the study due to low quality

Organism	Acc. No.	Raw Data	Assembly length (Mbp)	# Seq.	N50 (Mbp)	L50	BUSCO (C %)	Location	Lifestyle	Reference
***Pseudoxylaria* sp. Mn132**	JAJFDL000000000	I	35.0	342	0.26	46	96.6	South Africa	Termite-associated stowaway (*Macrotermes natalensis*)	This study
***Pseudoxylaria* sp. Mn153**	JAJFDM000000000	I	34.5	355	0.23	49	93.3	South Africa	Termite-associated stowaway (*Macrotermes natalensis*)	This study
***Pseudoxylaria* sp. X167**	JAJFDK000000000	I	33.2	454	0.22	48	95.2	South Africa	Termite-associated stowaway (*Odontotermes* sp.)	This study
***Pseudoxylaria* sp. X170**	JAJFDJ000000000	I	33.7	339	0.22	49	96.6	South Africa	Termite-associated stowaway (*Odontotermes* sp.)	This study
***Pseudoxylaria* sp. X187**	JAJIZN000000000	PB, ONT	39.7	34	2.94	6	96.4	South Africa	Termite-associated stowaway (*Macrotermes natalensis*)	This study
***Pseudoxylaria* sp. X3-2**	JAJFDI000000000	I, ONT	36.9	742	0.267	41	96.2	South Africa	Termite-associated stowaway (*Macrotermes natalensis*)	This study
***Pseudoxylaria* sp. X802**	JAJFDH000000000	I, ONT	40.4	106	0.63	20	96.4	South Africa	Termite-associated stowaway (*Microtermes* sp.)	This study
***Xylaria* sp. BCC 1067**	GCA_005188305.1	PB	54.1	43	5.57	5	-	Thailand: Phetchabun	Saprotroph; Isolated from petiole of *Nenga pumila*	Sutheeworapong et al. 2019[1]
***Xylaria* sp. JS573**	GCA_000966885.1	I	40.0	100	0.94	14	-	South Korea: Muju	Parasite; Isolated from *Phragmites australis*	-
***Xylaria* sp. MSU_SB201401**	GCA_002288965.1	IT	56.8	5,995	0.06	245	94.7	USA: Louisiana	Parasite: Isolated on soy bean roots	Sharma et al. 2018[2]
***Xylaria flabelliformis* G536**	GCA_007182795.1	I	41.2	155	0.49	28	93.6	USA: North Carolina	Saprotroph and Endophyte; Isolated on *Asimina triloba*	Mead et al. 2019[3]
***Xylaria grammica* EL000614**	GCA_004353285.1	PB, I	55.6	44	3.88	6	99.8	South Korea	Endolichenic	Park et al. 2021[4]
***Xylaria grammica* IHI A82**	GCA_004014815.1	IT	47.0	1,053	0.08	172	-	Kenya: Kakamega Forest	Endophyte; Isolated on deadwood	-
***Xylaria hypoxylon* CBS 122620**	GCA_902806585.1	I, ONT	54.3	88	3.89	6	-	-	Saprotroph	Wibberg et al. 2020[5]
***Xylaria hypoxylon* DSM 108379**	GCA_004768795.1	IT	42.8	635	0.12	102	96.0	Germany: Bad Lobenstein	Saprotroph; Isolated on deadwood	Büttner et al. 2019b[6]
***Xylaria longipes* IHI A66**	GCA_003426265.1	IT	43.2	1,006	0.07	185	90.6	Germany: Bavarian Forrest	Saprotroph; Isolated from *Acer pseudoplatanus*	Büttner et al. 2019a[7]
***Xylaria multiplex* DSM 110363**	GCA_011057905.1	IT	45.6	389	0.24	57	96.4	USA: Puerto Rico	Saprotroph; Isolated from deadwood	Büttner et al. 2020[8]
***Xylaria polymorpha* DSM 105756**	GCA_003426235.1	IT	43.5	947	0.07	173	-	Germany: Zittau, Westpark	Saprotroph; isolated from beech	-
**Xylaria striata RK1-1***	GCA_002749545.1	IT	59,8	79,106	0.001	15,261	-	China: Yunnan	Parasite: Isolated on rice roots	-

Table S5. *Xylaria* ITS reference sequences from NCBI fungal ITS RefSeq targeted loci database (marked in grey) and isolated *Pseudoxylaria* strains (upper part). Gene accession numbers of ITS sequences used for phylogenetic analysis as reference data was selected from Hsieh et al 2005 to provide a framework for phylogenetic classification (lower part).

Family	Genus	Species	Strain	Accession ID
	Amphirosellinia	*fushanensis*		NR_153514
	Amphirosellinia	*nigrospora*		NR_153513
	Biscogniauxia	*arima*		NR_167683
	Podosordaria	*muli*		NR_158883
	Poronia	*pileiformis*		NR_158882
	Xylaria	*acuminatilongissima*		NR_147516
	Xylaria	*bambusicola*		NR_153200
	Xylaria	*brunneovinosa*		NR_153201
	Xylaria	*ellisii*		NR_172972
	Xylaria	*eucalypti*		NR_166326
	Xylaria	*fabacearum*		NR_171104
	Xylaria	*fabaceicola*		NR_171103
	Xylaria	*hongkongensis*		NR_154905
	Xylaria	*insolita*		NR_171861
	Xylaria	*longissima*		NR_147567
	Xylaria	*ripicola*		NR_153251
	Xylaria	*subescharoidea*		NR_171862
	Pseudoxylaria	*sp.*	X802	KX097055.1
	Pseudoxylaria	*sp.*	Mn132	OM443072
	Pseudoxylaria	*sp.*	Mn153	MT012094.1
	Pseudoxylaria	*sp.*	X167	MT012092.1
	Pseudoxylaria	*sp.*	X170lb	MT012093.1
	Pseudoxylaria	*sp.*	X187	MN961667.1
	Pseudoxylaria	*sp.*	X3.2	MT012091
Amphispheraceae	*Iodosphaeria*	*foliicola*		NR_173980
	Iodosphaeria	*phyllophila*		NR_173981
	Neopestalotiopsis	*macadamiae*		NR_161002
Apiosporaceae	*Arthrinium*	*neosubglobosum*		NR_154737
Diatrypaceae	*Diatrype*	*lijangensis*		NR_165229
Hypoalaceae	*Hypoxylon*	*bellicolor*		NR_169971
	Hypoxylon	*eurasiaticum*		NR_172358
	Hypoxylon	*fuscum*		NR_172215
	Hypoxylon	*pseudofuscum*		NR_172359
	Hypoxylon	*sporistriatatunicum*		NR_169972
Leptosilaceae	*Furfurella*	*nigrescens*		NR_164061
	Furfurella	*stromatica*		NR_164062
	Leptosilia	*acerina*		NR_164063
	Leptosilia	*macrospora*		NR_164064
	Leptosilia	*muelleri*		NR_164065
	Leptosilia	*slaptonensis*		NR_164066
	Leptosilia	*wienkampii*		NR_164067
Pseudomassariaceae	*Pseudomassariella*	*vexata*		NR_164217
	Tristratiperidium	*microsporum*		NR_164238
Xylariaceae	*Calceomyces*	*lacunosus*		NR_167686

Table S6. Gene accession numbers of partial protein-coding genes ACT, RPB2 and TUB used for phylogenetic analysis and identified sequences from isolated *Xylaria* strains (lower part).

Clade	Genus	Species	Strain	TUB	ACT	RPB2
outgroup	*Annulohypoxylon*	*cohaerens*		AY951655.1	AY951766	GQ844766
	Biscogniauxia	*arima*		AY951672.1	AY951784	GQ304736.1
	Biscogniauxia	*mediterranea*		AY951684.1	AY951796.1	GQ844765
	Podosordaria	*mexicana*		AY951684.1	GQ455451.1	GQ853039
	Podosordaria	*muli*		GQ844839.1	GQ455450	GQ853038
	Poronia	*pileiformis*		GQ502720.1	GQ455449	GQ853037
HY	*Kretzschmaria*	*clavus*		EF025611.1	EF025596.1	GQ844789
	Kretzschmaria	*megalospora*		EF025609.1	EF025594.1	GQ844791
	Kretzschmaria	*sandvicensis*		GQ478211.1	GQ398234.1	GQ844786
	Xylaria	*adscendens*		GQ487709.1	GQ438746.1	GQ844818
	Xylaria	*arbuscula*		GQ478226.1	GQ421286.1	GQ844805.1
	Xylaria	*bambusicola*		GQ478223.1	GQ408910.1	GQ844801
	Xylaria	*coccophora*		GQ487701.1	GQ421289.1	GQ844809.1
	Xylaria	*hypoxylon*		GQ260187.1	GQ427196.1	GQ844812
	Xylaria	*liquidambaris*		GQ487702.1	GQ421290.1	GQ844810
	Xylaria	*multiplex*		GQ487705.1	GQ427198.1	GQ844814
	Xylaria	*oligotoma*		GQ487700.1	GQ421288.1	GQ844808
	Xylaria	*striata*		GQ478224.1	GQ421284.1	GQ844803
	Xylaria	*venustula*		GQ487699.1	GQ421287.1	GQ844807
NR	*Nemania*	*beaumontii*		GQ470222.1	GQ389694.1	GQ844772
	Nemania	*bipapillata*		GQ470221.1	GQ389693.1	GQ844771
	Nemania	*maritima*		GQ470225.1	GQ389697.1	GQ844775.1
	Rosellinia	*buxi*		GQ470228.1	GQ398228.1	GQ844780.1
	Rosellinia	*lamprostoma*		EF025604.1	EF025589.1	GQ844778
	Rosellinia	*merrillii*		GQ470229.1	GQ398229.1	GQ844781
PO	*Xylaria*	*frustulosa*		GQ495943.1	GQ449237.1	GQ844837
	Xylaria	sp.8		GQ495931.1	GQ438752.1	GQ844824
	Discoxylaria	*myrmecophila*		GQ487710.1	GQ438747.1	GQ844819.1
	Stilbohypoxylon	*elaeicola*		GQ495933.1	GQ438754.1	GQ844827
	Xylaria	*berteri*		GQ502698.1	GQ455442.1	GQ848363
	Xylaria	*oxyacanthae*		GQ495927.1	GQ438748.1	GQ844820
	Xylaria	*cubensis*		GQ502700.1	GQ455444.1	GQ848365
	Xylaria	*curta*		GQ495936.1	GQ438757.1	GQ844830
	Xylaria	*feejeensis*		GQ495945.1	GQ449241	GQ848334
	Xylaria	*laevis*		GQ502695.1	GQ455439.1	GQ848359.1
	Xylaria	*polymorpha*		GQ495954.1	GQ452364.1	GQ848343
	Xylaria	sp.7		GQ495928.1	GQ438749.1	GQ844821
	Xylaria	*apoda*		GQ495930.1	GQ438751.1	GQ844823.1
	Xylaria	*globosa*		GQ502684.1	GQ452369.1	GQ848348.1
	Xylaria	*scruposa*		GQ495952.1	GQ452362.1	GQ848341.1
TE	*Xylaria*	*acuminatilongissima*		GQ502711	GQ853046	GQ853028.1
	Xylaria	*atrodivaricata*		GQ502713	GQ853048	GQ853030
	Xylaria	*brunneovinosa*		GQ502706.1	GQ853041	GQ853023.1
	Xylaria	*cirrata*		GQ502707	GQ853042.1	GQ853024
	Xylaria	*escharoidea*		GQ502709	GQ853044	GQ853026.1
	Xylaria	*fimbriata*		GQ502705	GQ853040.1	GQ853022.1
	Xylaria	*griseosepiacea*		GQ502714	GQ853049.1	GQ853031
	Xylaria	*intraflava*		GQ502718	GQ853053	GQ853035.1
	Xylaria	*nigripes*		GQ502710	GQ853045	GQ853027
	Xylaria	*ochraceostroma*		GQ502717	GQ853052	GQ853034.1
	Xylaria	sp. 1		GQ502719.1	GQ853054	GQ853036.1
	Xylaria	sp. 2		GQ502708	GQ853043	GQ853025
	Xylaria	sp. 3		GQ502712	GQ853047	GQ853029.1
	Xylaria	sp. 4		GQ502715	GQ853050	GQ853032.1
	Xylaria	sp. 5		GQ502716	GQ853051	GQ853033.1
	Pseudoxylaria	*sp.*	X802	OM562439	OM562432	OM562446
	Pseudoxylaria	*sp.*	Mn132	OM562440	OM562433	OM562447
	Pseudoxylaria	*sp.*	Mn153	OM562441	OM562434	OM562448
	Pseudoxylaria	*sp.*	X167	OM562442	OM562435	OM562449
	Pseudoxylaria	*sp.*	X170lb	OM562443	OM562436	OM562450
	Pseudoxylaria	*sp.*	X187	OM562444	OM562437	OM562451
	Pseudoxylaria	*sp.*	X3.2	OM562445	OM562438	OM562452

Table S7. Primers and PCR conditions for amplification of fungal phylogenetic marker sequences

Gene	Sequence	t_R	Product length (bp)
ACT-512F	ATGTGCAAGGCCGGTTTCGC	66 °C	300
ACT-783R	TACGAGTCCTTCTGGCCCAT		
fRPB2-5F	GACGACAGAGATCATTTTGG	60 °C	1300
fRPB2-7cR	CCCATAGCTTGTTTACCCAT		
ITS 1	TCCGTAGGTGAACCTGCGG	55 °C	700
ITS 4	TCCTCCGCTTATTGATATGC		

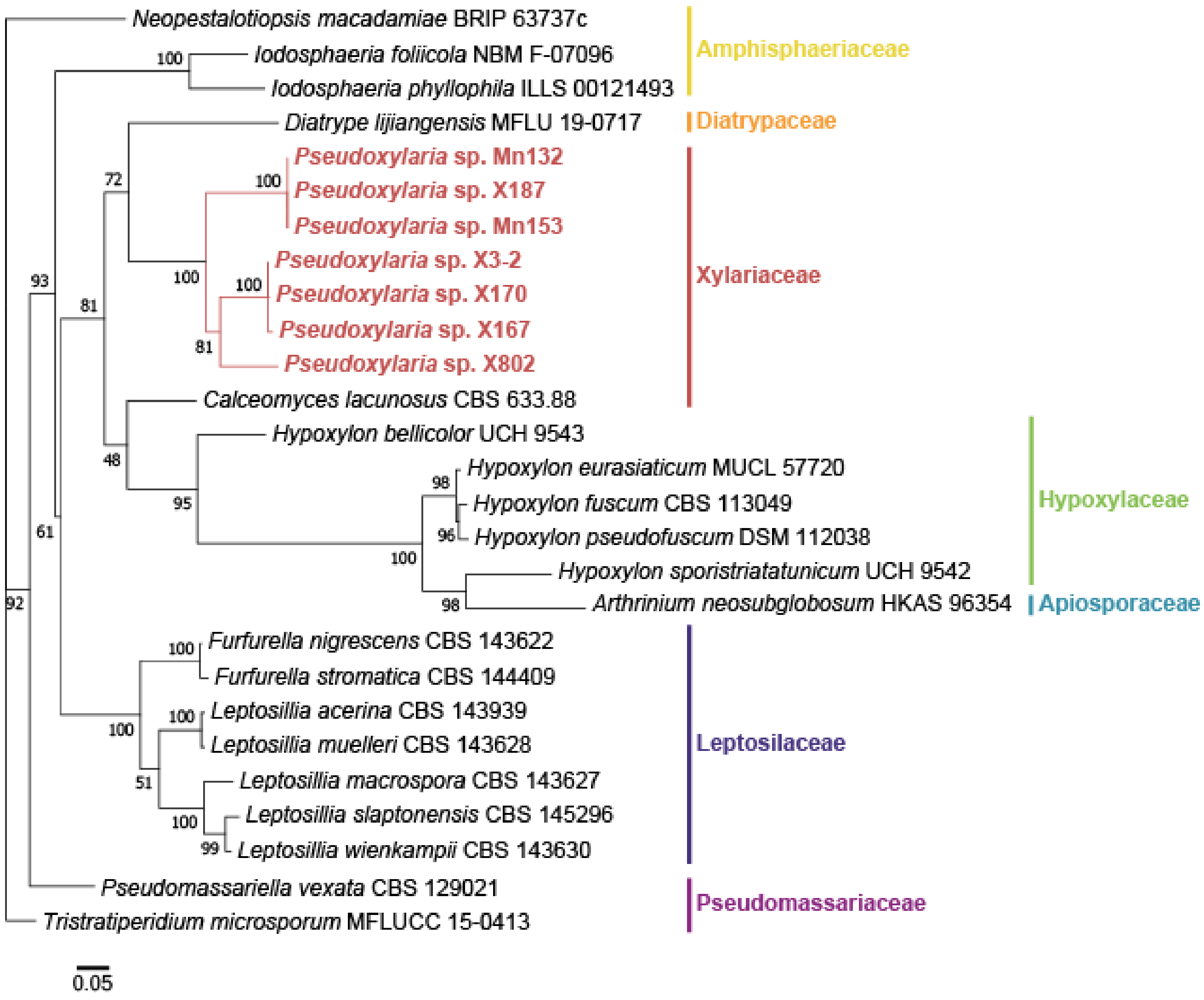

Figure S1. Maximum-likelihood ITS tree of *Xylariales* fungi calculated with 1000 bootstrap replicates. For tree generation, a symmetric model with unequal rates but equal base frequencies were used.[9] The model of rate heterogeneity is invariant using discrete gamma model with 4 categories.[10] The tree is unrooted.

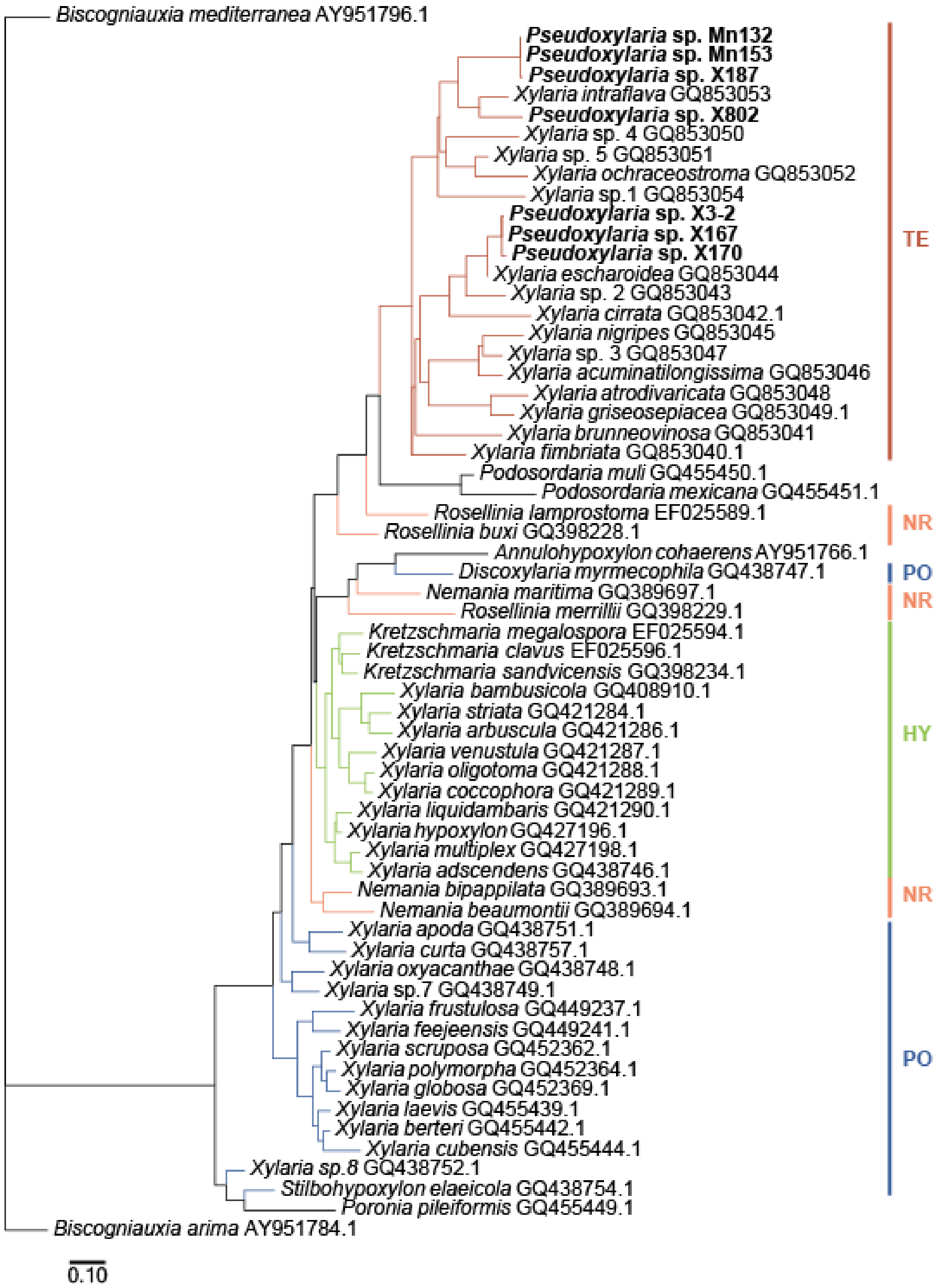

Figure S2. Maximum likelihood tree of *Xylaria* sp. using ACT as phylogenetic marker, calculated with 1000 bootstrap replicates. Clades are assigned according to Hsieh et al 2005.Phylogeny was predicted using TIM2 model as base substitution rates with empirical base frequencies. The model of rate heterogeneity is invariant using discrete gamma model with 4 categories. The tree is unrooted.

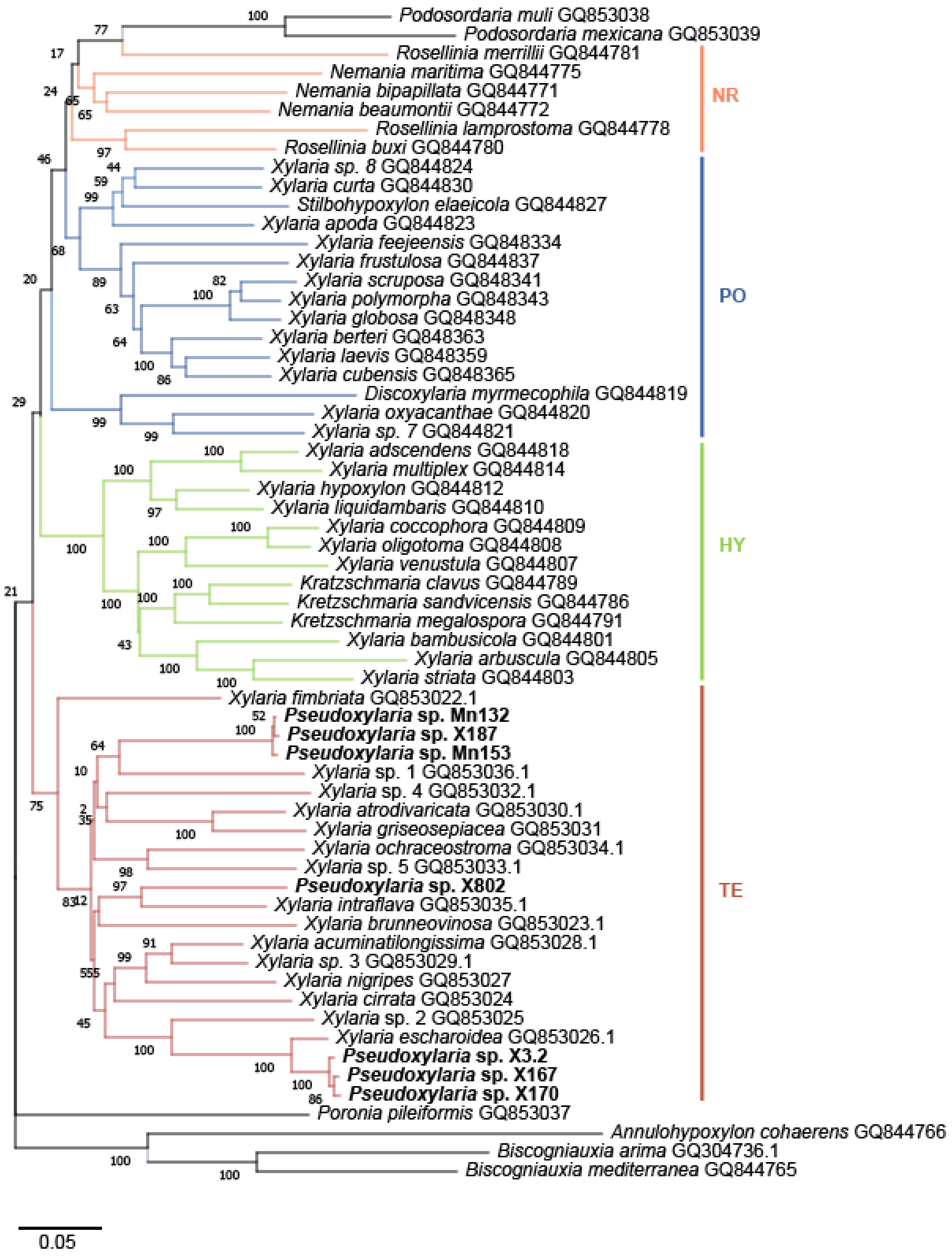

Figure S3. Maximum likelihood tree of *Xylaria* sp. using RPB2 as phylogenetic marker, calculated with 1000 bootstrap replicates. Clades are assigned according to Hsieh et al 2005. RPB2 gene tree was calculated using general time reversible model with unequal rates and empirical unequal base frequencies. For rate heterogeneity, a free rate model with 5 categories was used.

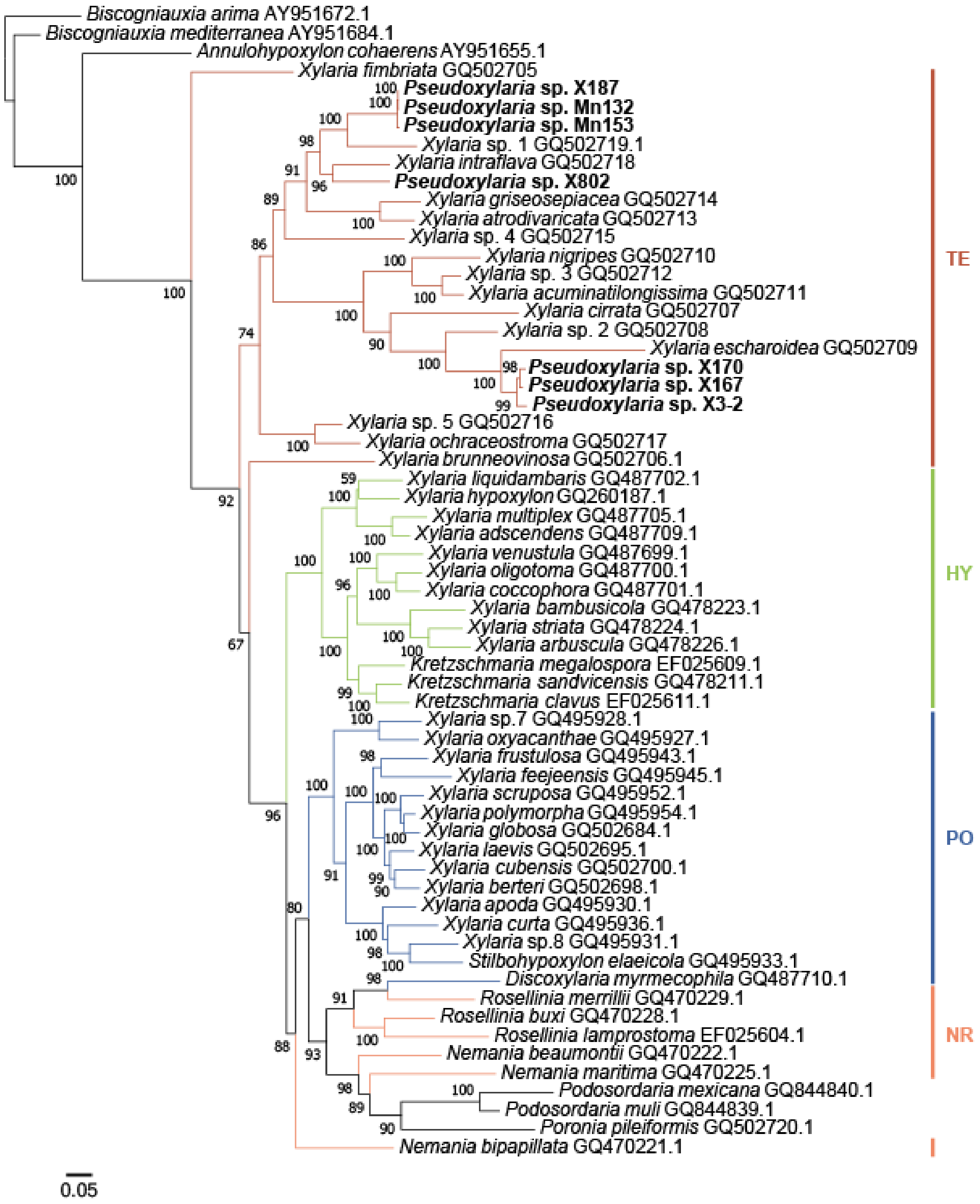

Figure S4. Maximum likelihood tree of *Xylaria* sp. using TUB as phylogenetic marker, calculated with 1000 bootstrap replicates. Clades are assigned according to Hsieh et al 2005. Phylogeny was predicted using TIM2 model as base substitution rates with empirical, unequal base frequency. For rate heterogeneity, a free rate model with 16 categories was used.

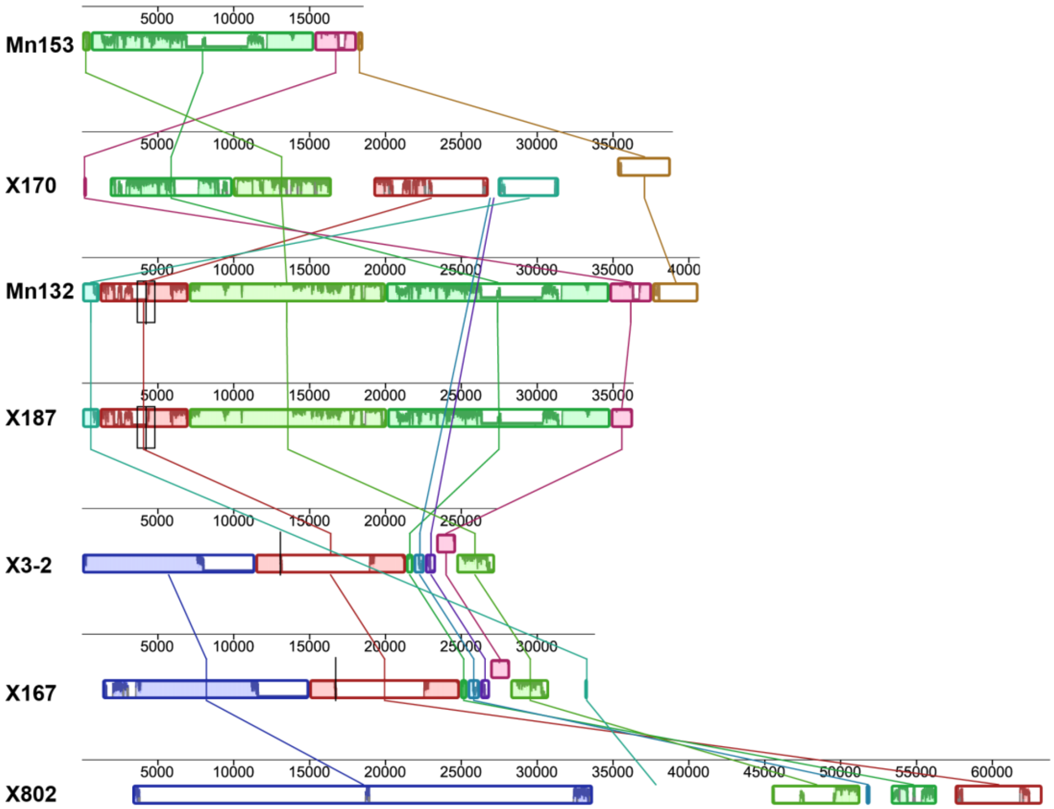

Figure S5. Mauve alignment of the *Pseudoxylaria* mitochondrial genome assemblies. For the assembly the software Mauve was used.[11] Locally collinear blocks are highlighted in the same color.

Table S8. Mitochondrial assemblies and annotation statistics of species from the Xylariales used in this study.

Organism	Acc. No.	Genome length (kbp)	Circular assembly	Annotated genes	Annotated tRNAs	Reference
Pseudoxylaria						
Mn132	OL598078	40.7	Yes	4	13	This study
Mn153	OL598079	18.6	Yes	4	13	This study
X167	OL598081	33.8	Yes	7	14	This study
X170	OL598082	38.9	Yes	12	13	This study
X187	OL598083	36.4	Yes	5	12	This study
X3-2	OL598080	27.4	Yes	8	13	This study
X802	OL598084	63.8	Yes	13	22	This study
**Xylariales**						
Annulohypoxylon stygium	MH620794.1	143.3	Yes	60	26	Deng et al. 2018[12]
Annulohypoxylon stygium	KF545917.1	133.8	Yes	58	26	n/a (direct submission)
Arthrinium arundinis	KY775582.1	49.0	Yes	45	23	n/a
Nemania diffusa	MN780510.1	258.9	Yes	41	25	Tang et al. 2020[13]
Pestalotiopsis fici	KX870077.1	69.5	Yes	78	32	Zang et al. 2017[14]
Xylaria hypoxylon	NC_046734.1	129.3	Yes	48	27	Zhou et al. 2019[15]

Table S9. Identified transposable elements (TEs) in *Pseudoxylaria* and *Xylaria* species using EDTA.

Organism	LTR			TIR					nonTIR		% of
	Copia	Gypsy	unknown	CACTA	Mutator	PIF_Harbinger	Tc1_Mariner	hAT	Helitron	total	genome
***Pseudoxylaria* spp.**											
Mn132	0	58	45	163	204	59	52	149	382	1112	2.30
Mn153	81	28	27	221	310	26	8	27	239	967	2.16
X167	104	52	79	34	119	55	4	56	86	589	2.17
X170	101	31	2	26	33	6	1	10	19	230	1.53
X187	90	281	344	255	616	50	18	99	451	2204	5.16
X3-2	254	956	106	43	227	32	7	23	53	1703	5.53
X802	357	842	667	451	800	132	8	253	395	3905	9.93
mean	141	321	181	170	330	51	14	88	232	1530	4.11
***Xylaria* spp.**											
X. flabelliformis G536	0	10	0	31	35	0	10	3	33	122	1.30
X. grammica EL372	0	112	52	206	326	363	219	4	904	2186	2.45
X. grammica IHI A82	0	8	72	562	79	105	9	68	1163	2066	2.41
X. hypoxylon CBS 122620	1087	0	1711	283	306	75	35	28	612	4137	7.48
X. hypoxylon DSM 108379											
X. longipes IHI A66	16	10	50	754	791	8	72	513	1459	3673	2.94
X. multiplex DSM 110363	70	61	937	705	347	131	7	173	1667	4098	3.07
X. polymorpha DSM 105756	0	7	298	974	182	76	5	155	1523	3230	2.65
Xylaria sp. BCC 1067	0	0	506	180	610	12	15	452	1836	3611	2.79
Xylaria sp. JS573	0	111	59	76	133	3	147	46	323	898	1.66
Xylaria sp. MSU_SB201401	2853	0	722	511	1507	212	19	250	555	6629	8.10
mean	490	32	429	533	595	74	44	265	1227	3690	3.54

(**LTR**) long terminal repeats retrotransposons; (**TIR**) Two inverted tandem repeats DNA transposons

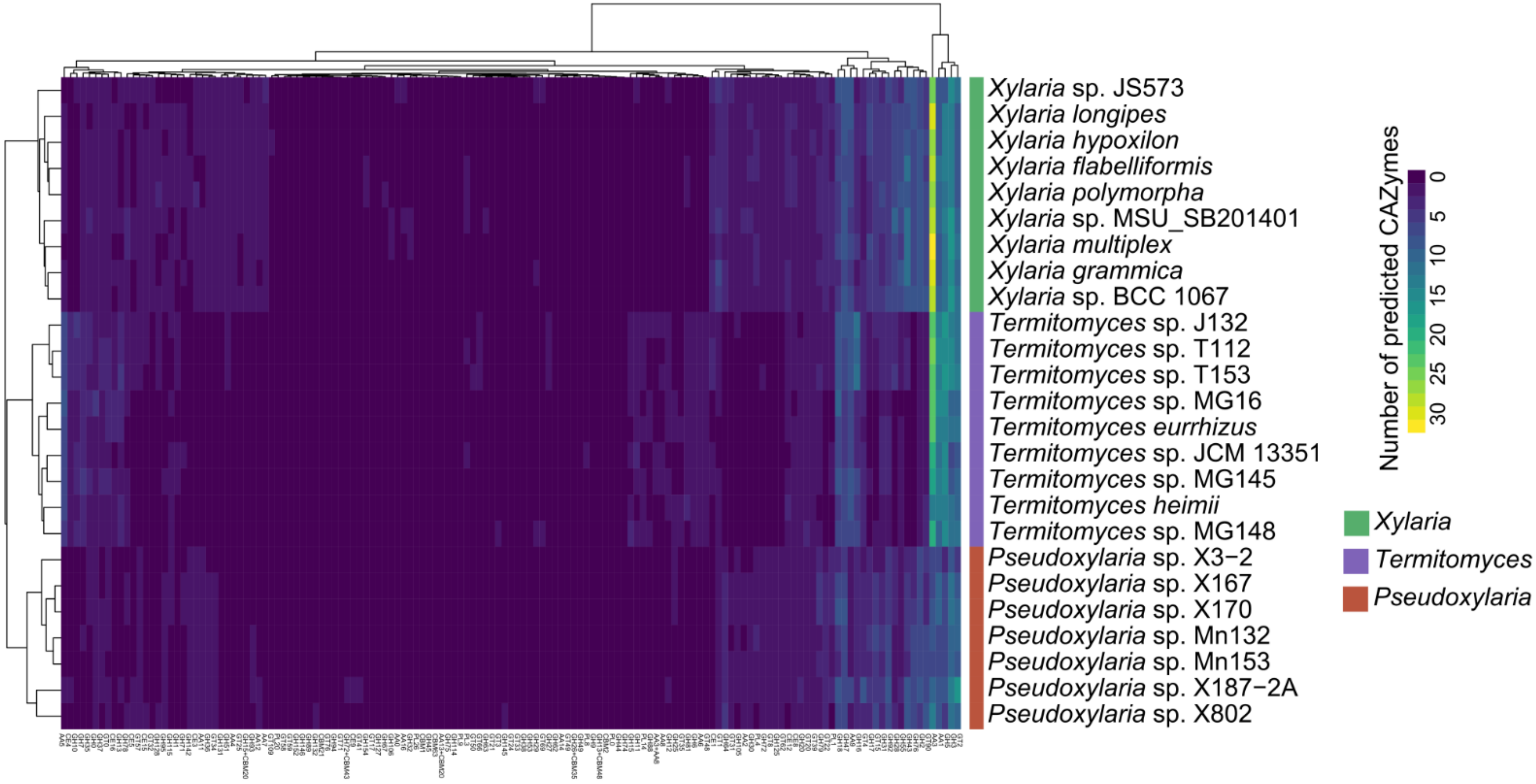

Figure S6. Comparative CAZY analysis of free-living *Xylaria*, termite-associated *Pseudoxylaria*-clade and *Termitomyces*.

Table S10. Additional identified redox active enzymes in *Pseudoxylaria* and *Xylaria* species.

Organism	Benzoquinone Reductase	Catalase	Gluthathione Peroxidase	HAO	Laccase	MnP	Peroxiredoxin	Superoxide Dismutase	DyP	UPO	total
***Pseudoxylaria* spp.**											
Mn132	1	5	1	4	7	1	1	2	1	0	23
Mn153	1	4	1	4	6	1	1	2	1	0	21
X167	1	4	1	4	11	1	1	2	1	0	26
X170	1	5	1	4	10	1	1	2	1	0	26
X187	2	5	2	4	7	1	1	3	1	0	26
X3-2	1	5	1	4	12	1	1	4	1	0	30
X802	1	2	1	4	8	1	1	2	1	0	21
mean	1.1	4.3	1.1	4	8.7	1	1	2.4	1	0	24.7
***Xylaria* spp.**											
X. flabelliformis G536	1	3	1	4	12	2	1	2	1	2	29
X. grammica EL372	1	4	1	4	10	2	1	2	1	2	28
X. grammica IHI A82	1	5	1	6	10	2	1	2	1	2	31
X. hypoxylon CBS 122620	1	4	1	4	9	2	1	2	1	2	27
X. hypoxylon DSM 108379	1	6	1	3	8	2	1	2	1	2	27
X. longipes IHI A66	1	5	1	6	7	2	1	2	2	2	29
X. multiplex DSM 110363	1	4	1	5	8	2	1	2	1	2	27
X. polymorpha DSM 105756	1	5	1	4	9	2	1	2	1	1	27
Xylaria sp. BCC 1067	1	4	1	4	10	2	1	2	1	1	27
Xylaria sp. JS573	1	3	1	3	6	2	2	2	0	0	20
Xylaria sp. MSU_SB201401	1	1	1	6	13	2	1	2	1	2	30
mean	1	4	1	4.5	9.3	2	1.1	2	1	1.6	27.5

(**MnP**) manganese-dependent peroxidase; (**DyP**) dye-decolorization peroxidase; (**HAO**) hydroxyl acid oxidase; (**UPO**) unspecific peroxidase

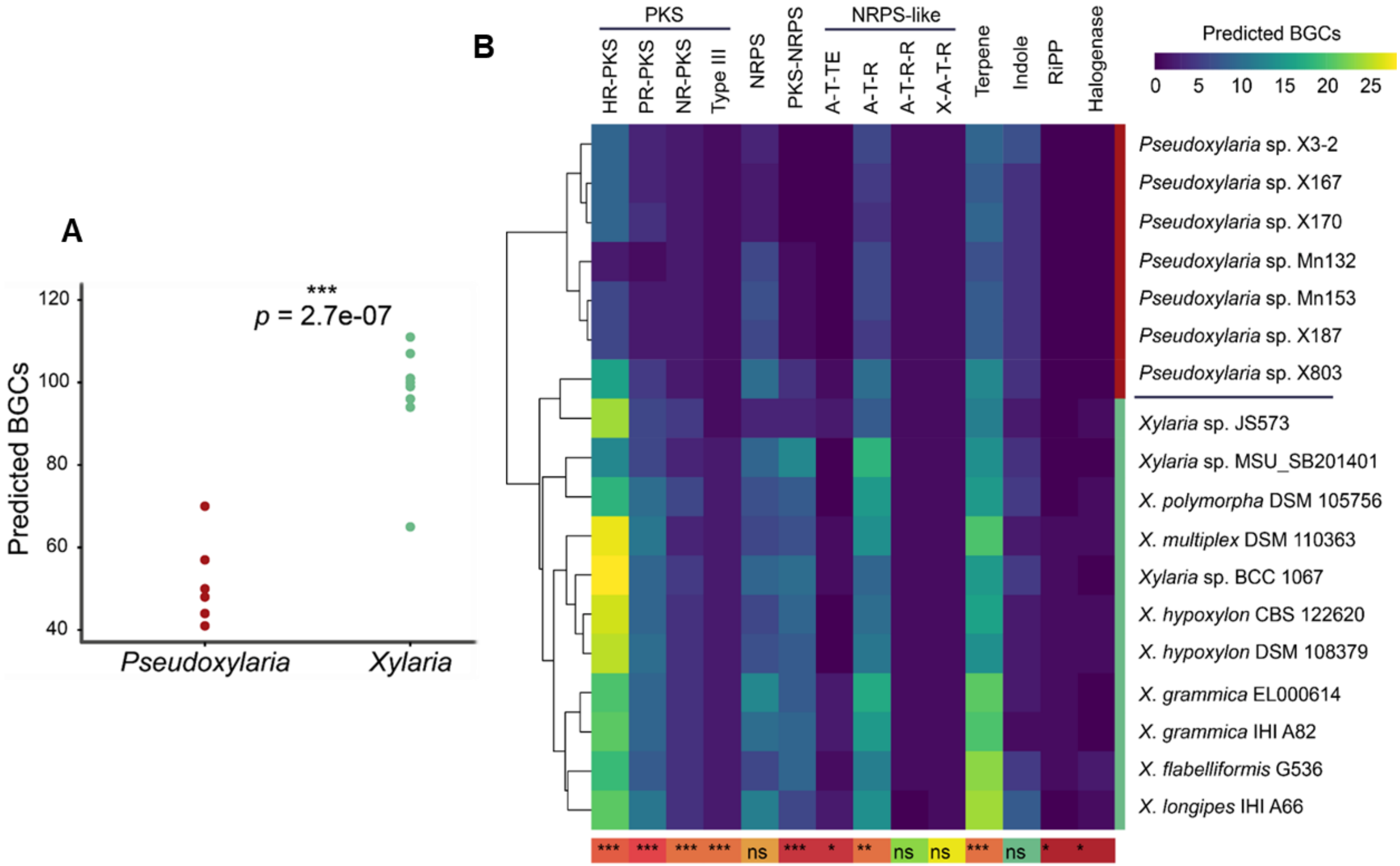

Figure S7. A) Numbers of BGCs classes identified in the genomes of *Pseudoxylaria* and free-living *Xylaria* species. **B**) Biosynthetic gene cluster analysis of *Pseudoxylaria* isolates. **A**) Heatmap depicting the numbers of identified BGCs classes annotated as polyketide synthases (PKS), non-ribosomal peptide synthases (NRPS), polyketide non-ribosomal peptide hybrid synthases (PKS-NRPS), NRPS-like synthases (with a domain architecture of A-T-TE and A-T-R), terpene synthases, ribosomally-synthesized and post-translationally modified peptides (RiPP), and halogenases. Shown on the bottom is the ratio of mean BGC numbers per group from not-significant (green) to significant (***red).

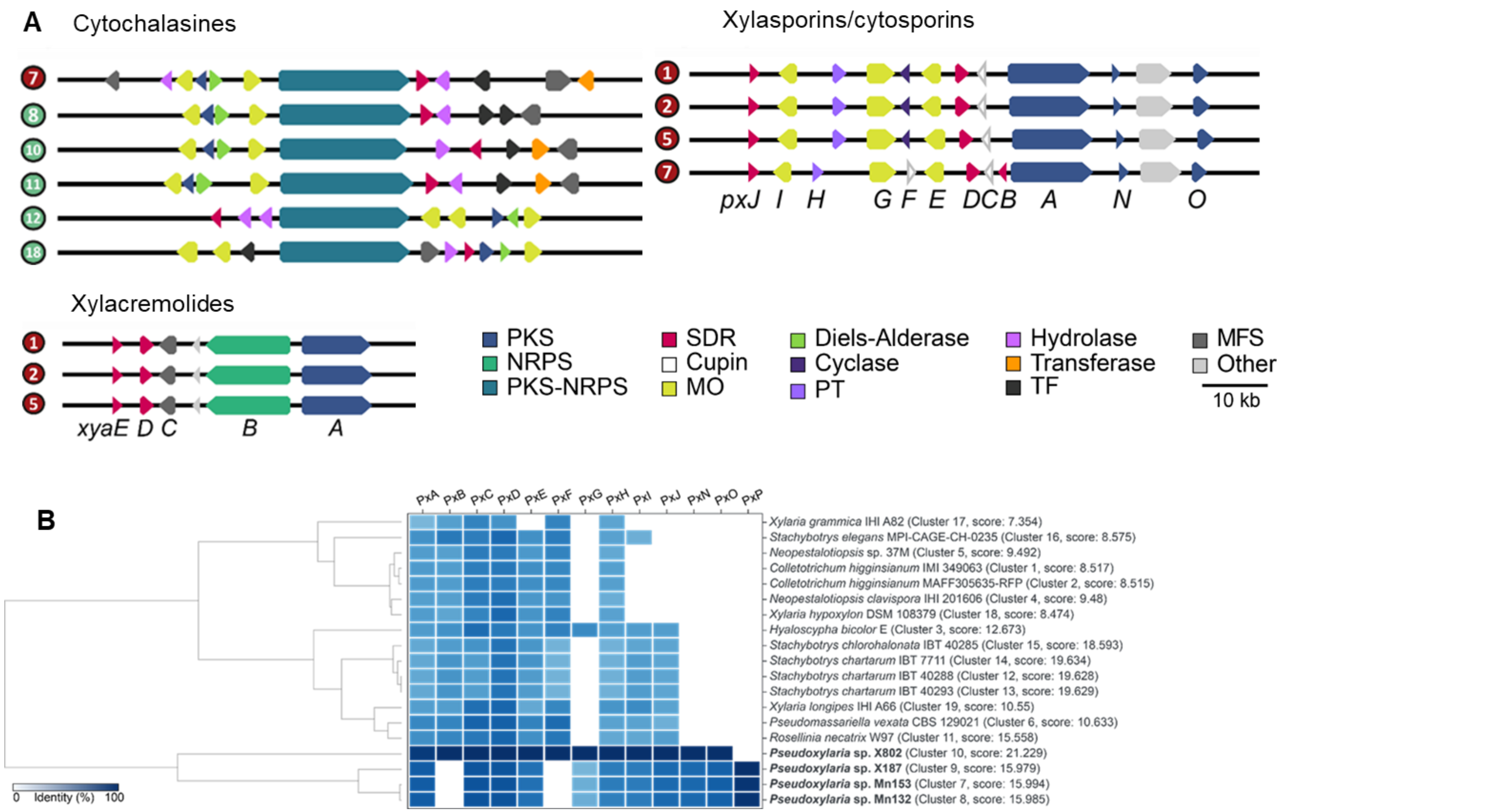

Figure S8. BGCs encoding for the productions of cytochalasan, xylasporin/cytosporin, and xylacremolide (polyketide synthases (PKS), non-ribosomal peptide synthetases (NRPS), PKS-NRPS hybrids, short-chain reductases (SDR), cupin-fold oxidoreduxtases (cupin), monooxygenases (MO), Diels-Alderases, SnoaL-like cyclases (cyclase), aromatic ABBA-type prenyltransferases (PT), α-β-hydrolases (hydrolase), acyl transferases (transferase), transcription factors (TF), and major facilitator type transporters (MFS). Red circled numbers indicate the different strains: (1) *Pseudoxylaria* spp. Mn132, (2) Mn153, (5) X187, (7) X802, (8) *Xylaria* spp. BCC 1067, (10) MSU_SB201401, (11) *X. flabeliformis* G536, (12) *X. grammica* EL000614, and (15) *X. hypoxylon* DSM 108379.Identified homologous biosynthetic genetic loci of the cytosporin/xylasporin (*px*) gene cluster. B) Heatmap showing the abundance and identity in % (white to dark blue) of co-localized homologous *px* genes in other ascomycete genomes deposited in the NCBI database was generated using cblaster v1.3.11.[16]

Table S11. Schematic representation of co-cultivation set-ups to determine inhibition of growth: Method **A)** Simultaneous co-cultivation of *Termitomyces* sp. T153 and *Pseudoxylaria* sp. (black square) after 2 or 3 weeks (W), and Method B) *Pseudoxylaria* sp. (black circle); was first incubated on a PDA plate and then challenged with *Termitomyces* sp. T153.

**Method**			**Method A**		**Method B**
**Strain**	***Pseudoxylaria* sp. X802 (2 W)**	***Termitomyces* sp. T153 (3W)**	**X802 (2W) - T153 (2W)**	**X802 (3w) - T153 (3W)**	**X802 (3W)-T153 (2W)**
**X802**	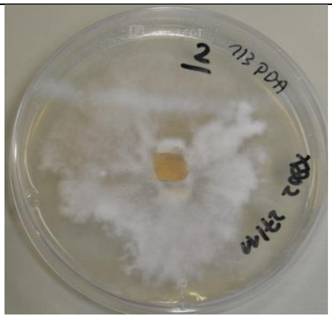	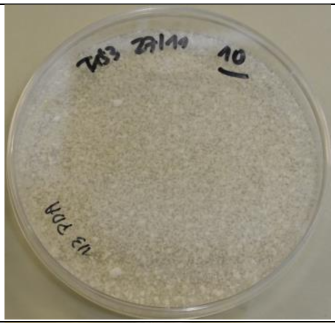	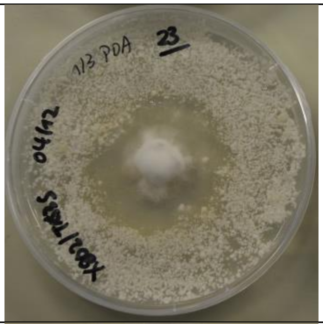		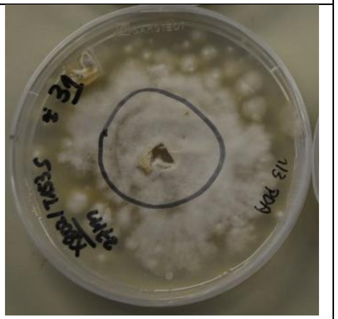
**MN154**	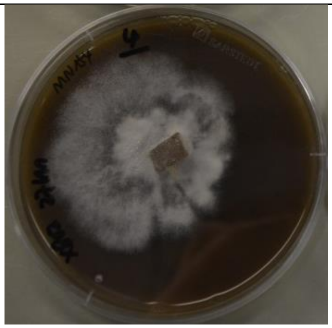	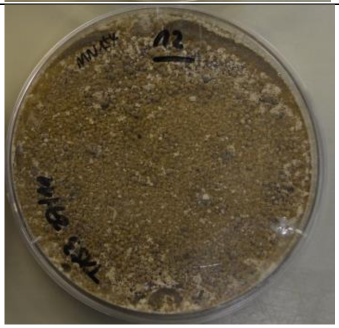	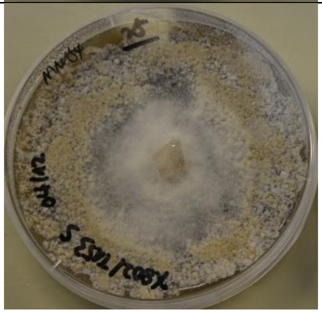	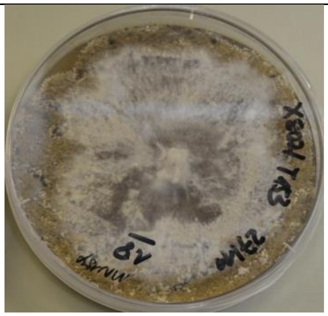	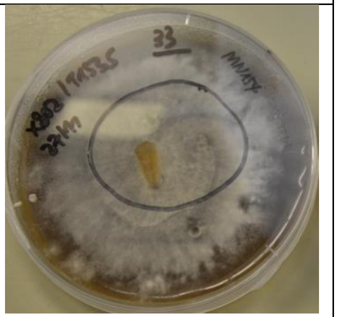
**MN164**	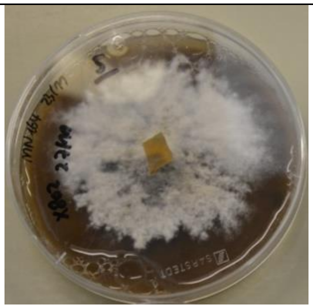	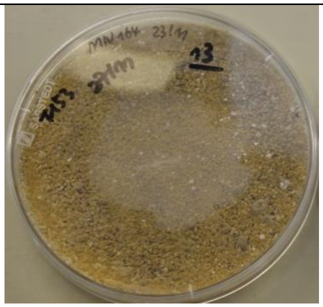	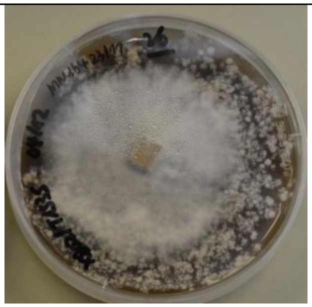	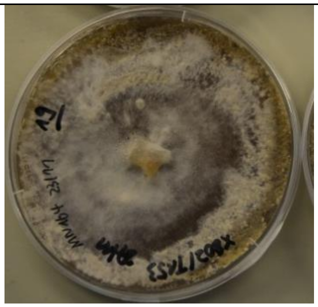	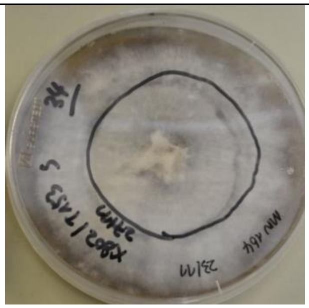

	Pseudoxylaria sp. X802	*Termitomyces* sp. T153 (3W)	X802 (2W)- T153 (2W)	X802 (3w)-T153 (3W)	X802 (3W)-T153 (2W)
**MN165**	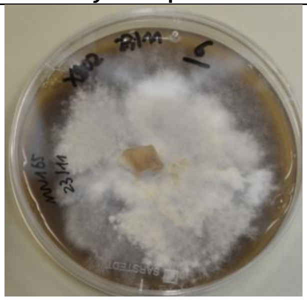	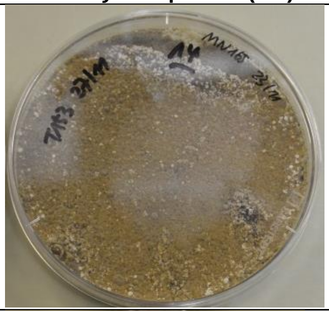	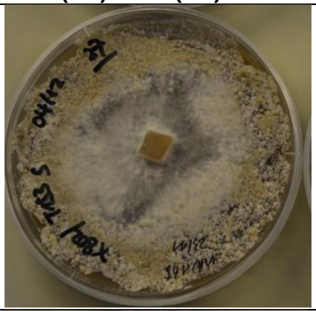		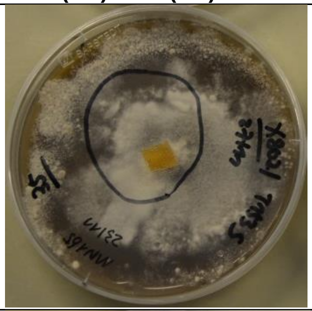
**MN166**	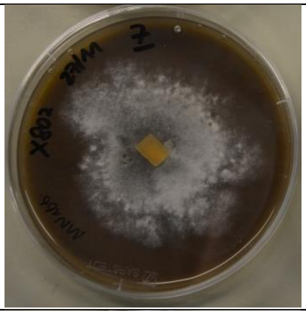	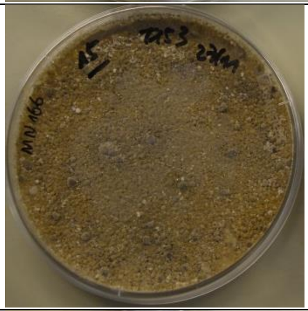	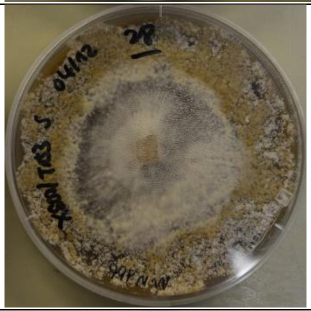	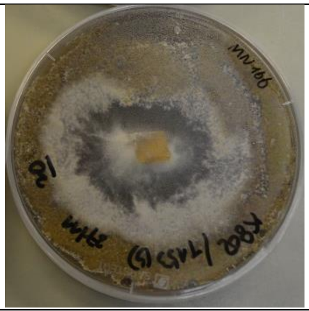	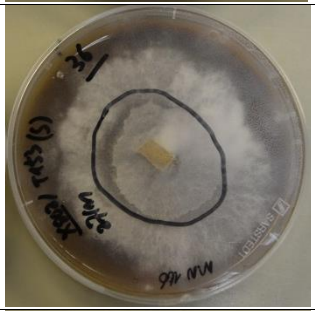
**MN171**	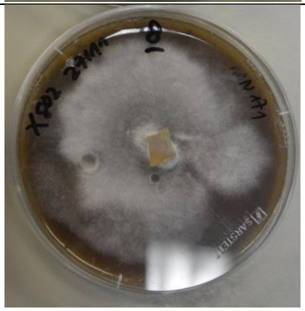	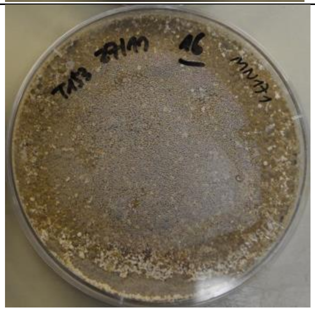	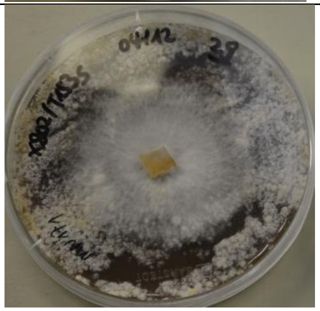		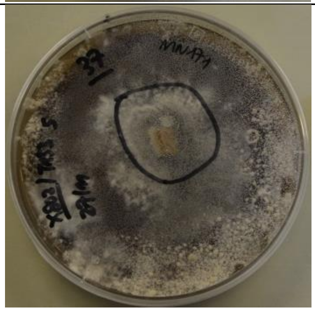

Table S12. Pictures of co-culture studies and axenic controls of *Termitomyces* sp. T153 and *Pseudoxylaria* sp. X170LB, X802 on PDA media after 6, 12 and 35 days. Inoculation of *Pseudoxylaria* (black square) was performed A) **on top of** a vegetative *Termitomyces* culture (grey color), B) **next to** a vegetative *Termitomyces* culture or C) both fungi were inoculated **simultaneously**.

	**6 days**	**12 days**	**35 days**
**Axenic control** *Pseudoxylaria* sp. X170LB	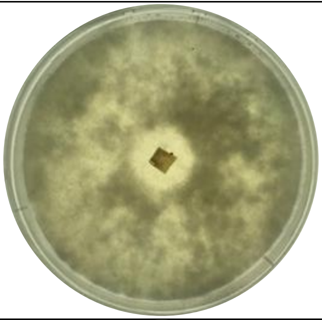	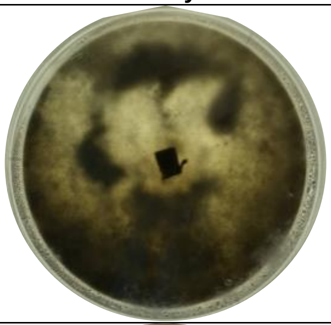	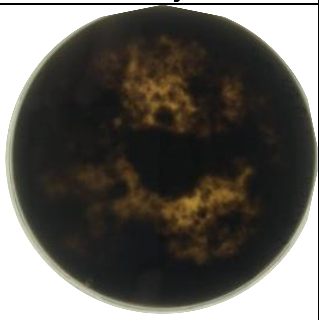
Pseudoxylaria sp. X170LB **on top of** *Termitomyces* sp. T153 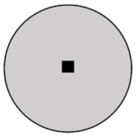	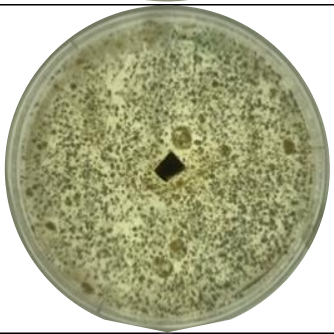	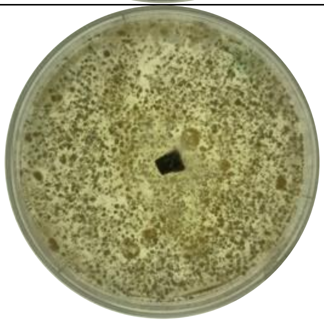	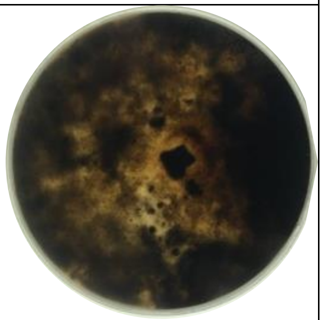
Pseudoxylaria sp. X170LB **next to** *Termitomyces* sp. T153 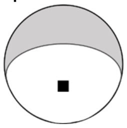	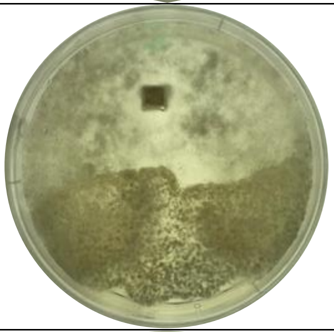	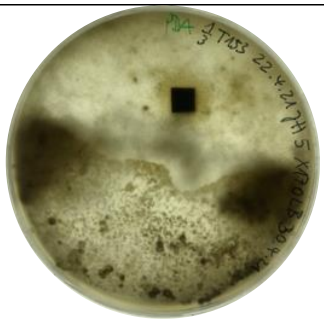	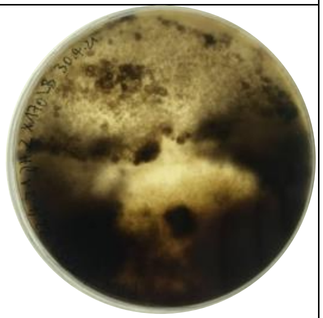
Pseudoxylaria sp. X170LB inoculated **simultaneously** with *Termitomyces* sp. T153 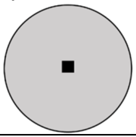	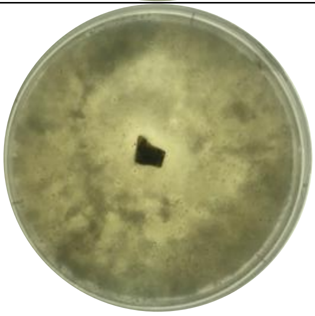	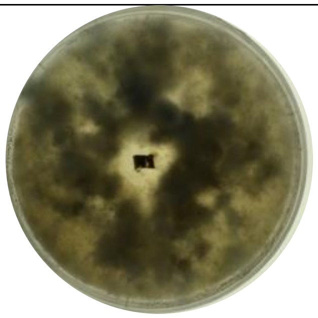	

Axenic control<br>*Pseudoxylaria*<br>sp. X802	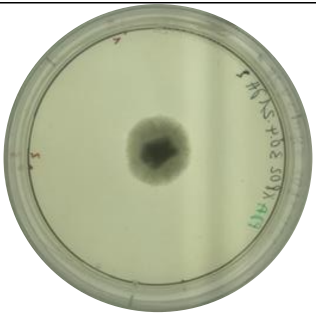	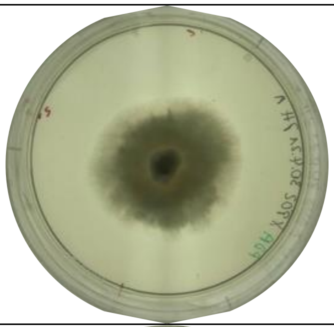	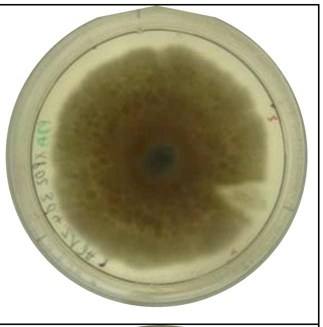
Pseudoxylaria<br>sp. X802<br>**on top of**<br>*Termitomyces*<br>sp. T153<br>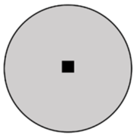	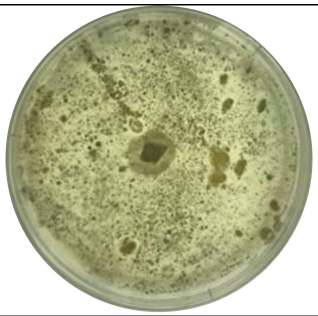	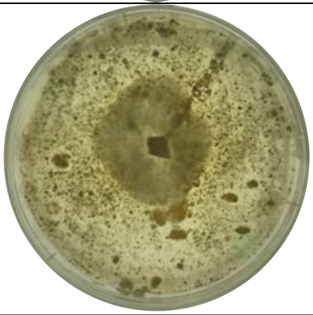	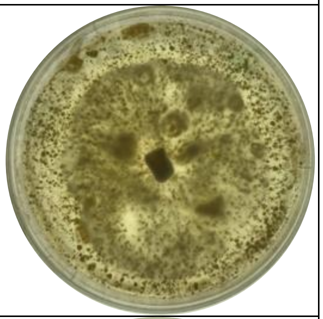
Pseudoxylaria<br>sp. X802<br>**next to**<br>*Termitomyces*<br>sp. T153<br>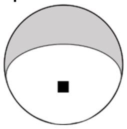	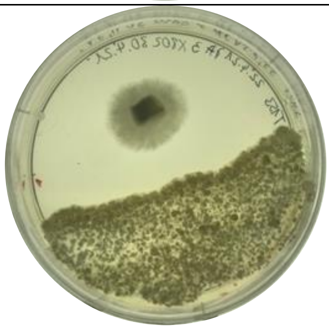	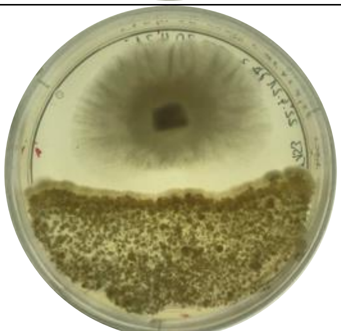	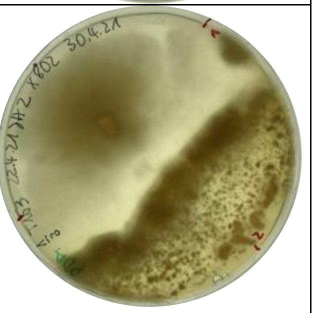
Pseudoxylaria<br>sp. X802<br>**co-inoculated**<br>**simultaneously**<br>on top of<br>*Termitomyces*<br>sp. T153<br>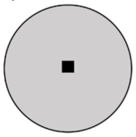	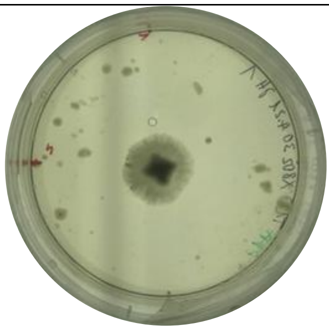	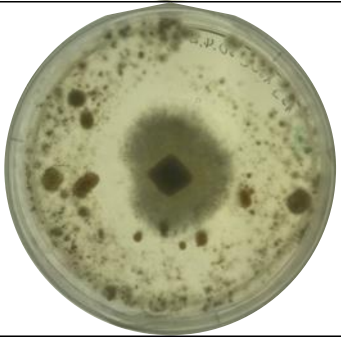	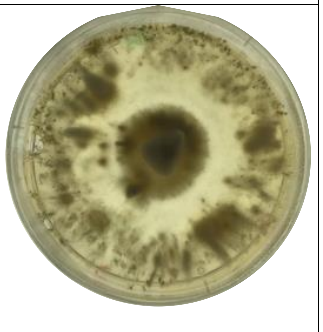

Table S13. Pictures of *Pseudoxylaria* sp. X170LB and X802 cultures on water-agarose medium and water-agarose medium supplemented with lyophilized *Termitomyces* sp. T112 biomass or 1/3 PDA

**Days**	**4**	**8**	**14**
Axenic control *Pseudoxylaria* sp. X170LB **on water-agarose medium**	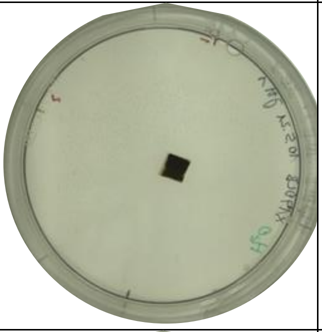	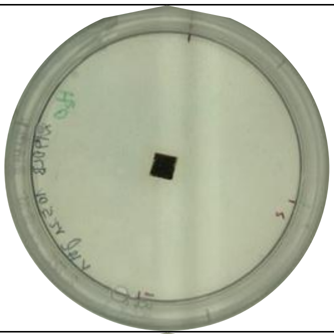	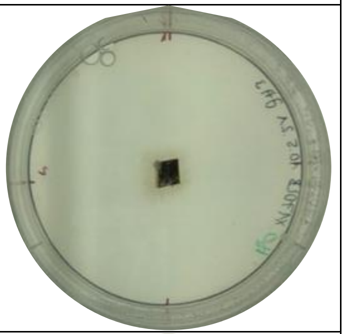
Pseudoxylaria sp. X170LB **on water/agarose containing *Termitomyces* sp.** T112 biomass	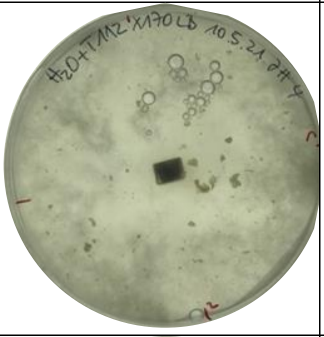	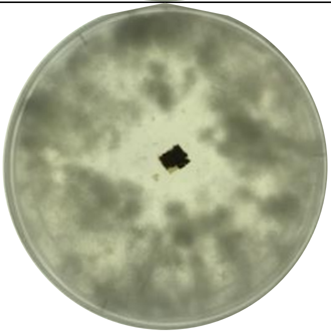	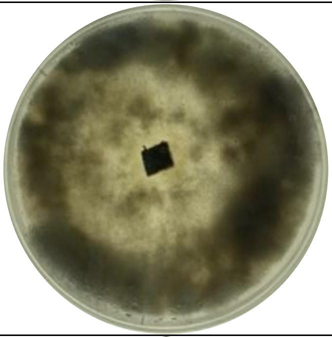
Pseudoxylaria sp. X802 **on water-agarose medium containing 1/3PDA**	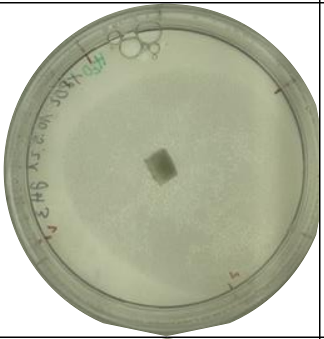	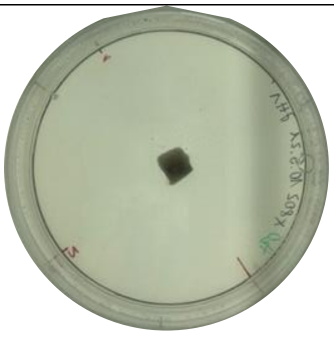	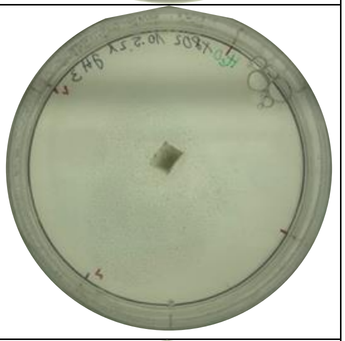
Pseudoxylaria sp. X802 **on water/agarose containing *Termitomyces* sp. T112 biomass and 1/3 PDA**	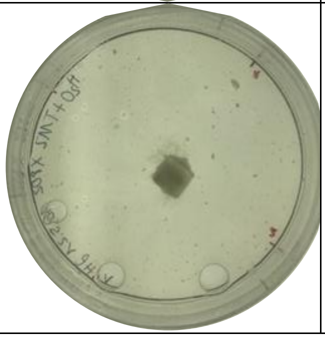	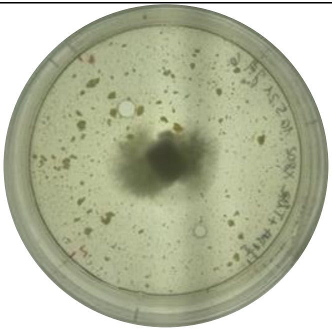	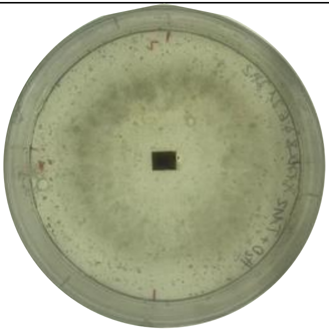

Table S14. Pictures of fungal co-cultures of different *Termitomyces* (T112, T153, P5HKI) and *Pseudoxylaria* strains (X802, X170LB) on wood-rice medium (WRM).

Day	7	17	31
Axenic control *Termitomyces* sp. T112 on wood-rice medium	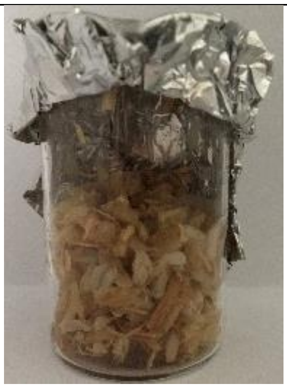	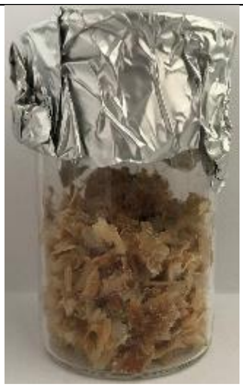	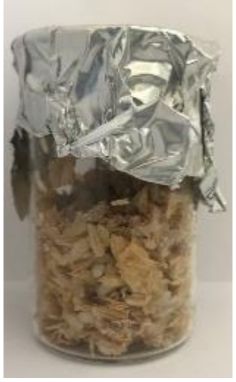
Axenic control *Termitomyces* sp. T153 on wood-rice medium	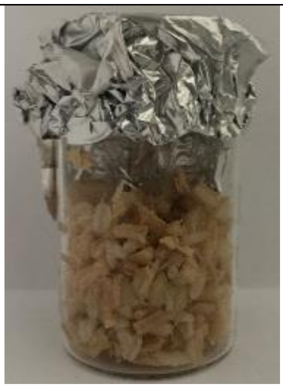	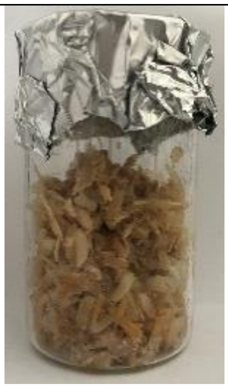	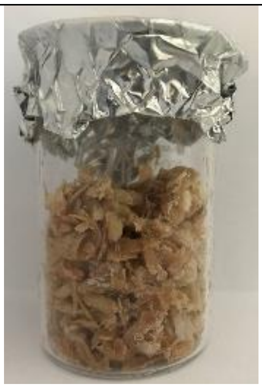
Axenic control *Termitomyces* sp. P5HKI on wood-rice medium	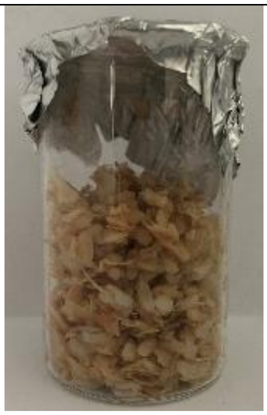	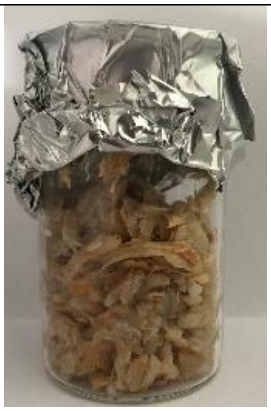	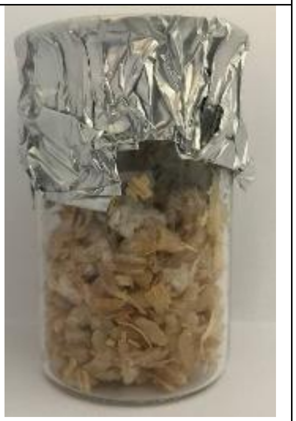

**Days after co-inoculation**	**3**	**10**	**21**
Axenic control *Pseudoxylaria* sp. X170LB on wood-rice medium	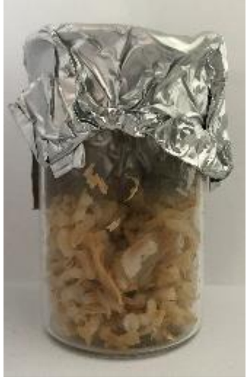	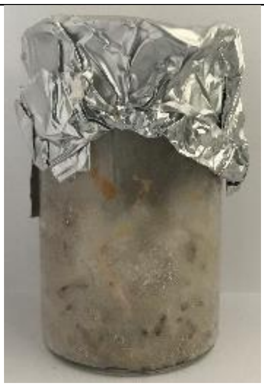	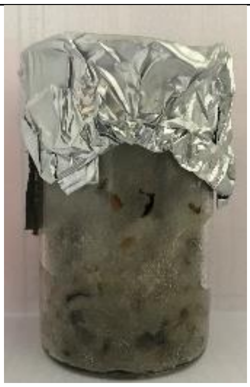
Axenic control *Pseudoxylaria* sp. X802 on wood-rice medium	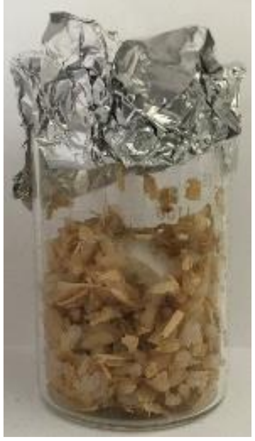	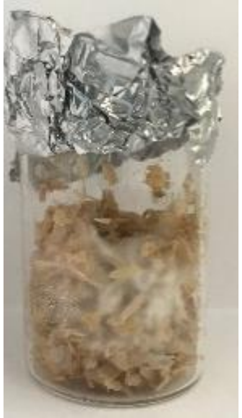	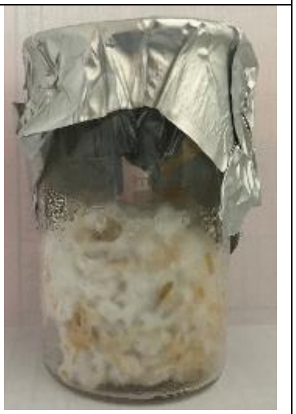
**Co-culture** *Termitomyces* sp. P5HKI and *Pseudoxylria* sp. X170LB on wood-rice medium	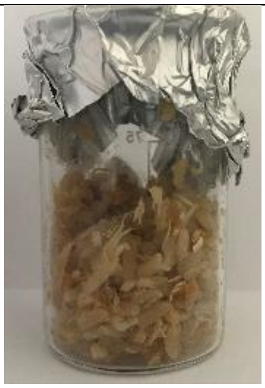	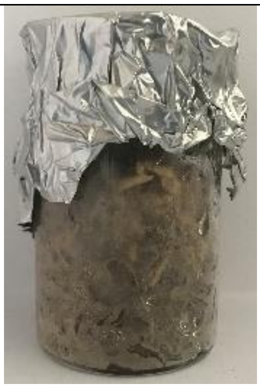	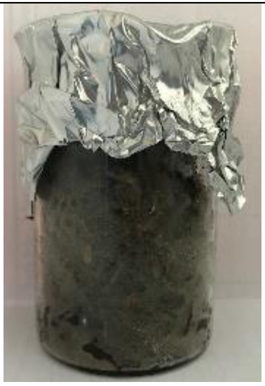

**Co-culture** *Termitomyces* sp. P5HKI and *Pseudoxylria* sp. X802 on wood-rice medium	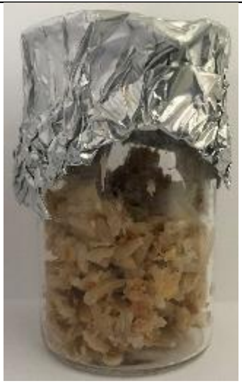	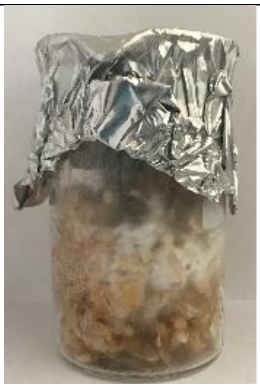	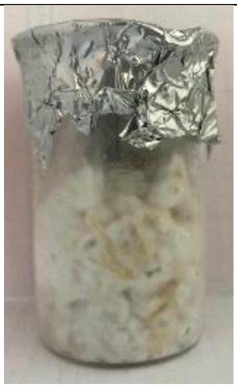
**Days after co-inoculation**	**3**	**10**	**21**
**Co-culture** *Termitomyces* sp. T112 and *Pseudoxylria* sp. X170LB on wood-rice medium	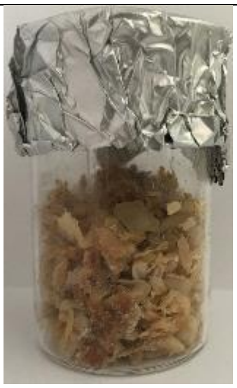	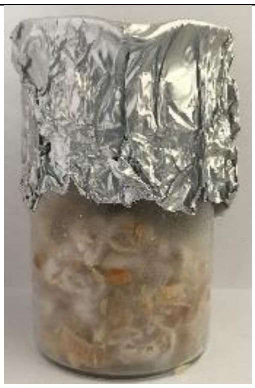	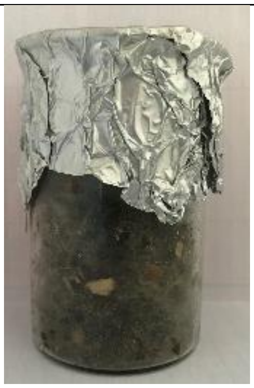
**Co-culture** *Termitomyces* sp. T112 and *Pseudoxylria* sp. X802 on wood-rice medium	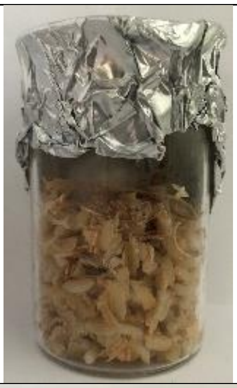	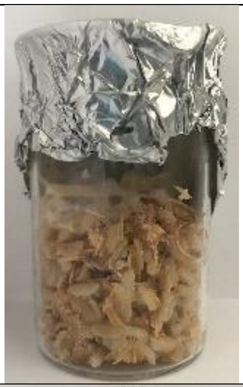	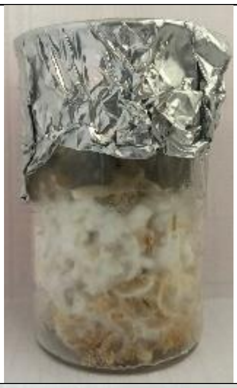
**Co-culture** *Termitomyces* sp. 153 and *Pseudoxylria* sp. X170LB on wood-rice medium	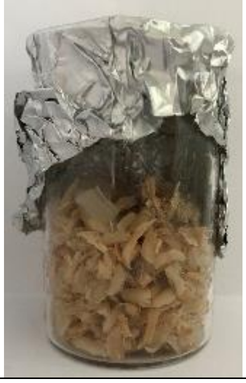	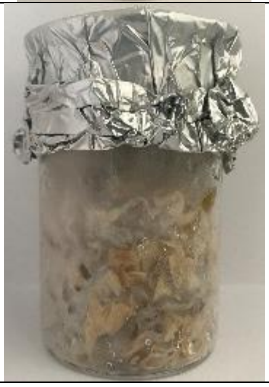	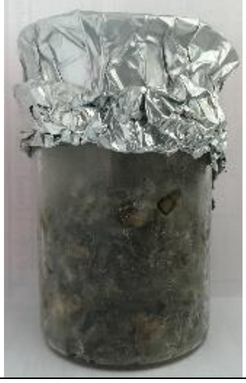

**Co-culture**<br>*Termitomyces* sp. 153 and *Pseudoxylria* sp. X802 on wood-rice medium	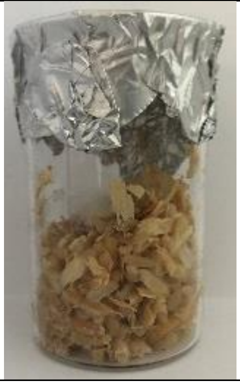	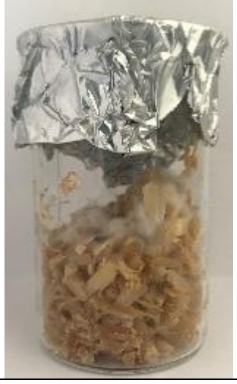	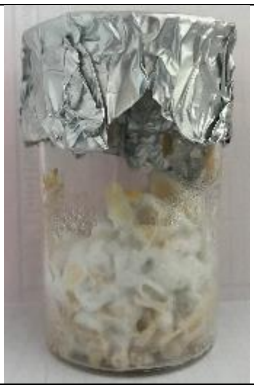

Table S15. Results for stable carbon isotopic fractionation experiments for sugars and lipids performed with *Termitomyces* sp. T112, *Pseudoxylaria* sp. X170LB in axenic cultures and cocultures.

**Media**	**Sample**	**Replicates (*n*)**	**$\Delta^{13}C/^{12}C$ Lipids**	**$\Delta^{13}C/^{12}C$ Sugars**	**Relative change lipids**	**Relative change sugars**
PDA	T112	3	-29.99 ± 0.12	-24.94 ± 0.07	-7.15 ± 0.49	-2.10 ± 0.48
PDA	X170LB	3	-26.76 ± 0.87	-24.11 ± 0.17	-3.92 ± 0.78	-1.27 ± 0.56
PDA	X170LB on vegetative T112 biomass	3	-26.95 ± 0.63	-23.87 ± 0.30	-4.11 ± 0.16	-1.03 ± 0.28
PDA	X170Lb on lyophilized T112 biomass	3	-25.05 ± 1.33	-23.57 ± 0.25	-2.21 ± 0.28	-0.73 ± 0.31
^{13}C-PDA	T112	3	-27.86 ± 0.09	-22.33 ± 0.25	-6.74 ± 0.99	-1.22 ± 0.50
^{13}C-PDA	X170LB	3	-24.23 ± 0.90	-22.43 ± 0.31	-3.11 ± 1.41	-1.31 ± 0.53
^{13}C-PDA	X170LB on vegetative T112 biomass	3	-24.69 ± 0.24	-20.50± 0.28	-3.58 ± 0.91	0.62 ± 0.32
^{13}C-PDA	X170Lb on lyophilized T112 biomass	3	-23.07 ± 0.46	-22.97 ± 0.27	-1.95 ± 0.48	-1.86 ± 0.31
PDA	Media average: -22.84 ± 0.47					
^{13}C PDA	Media average: -21.11 ± 0.13					

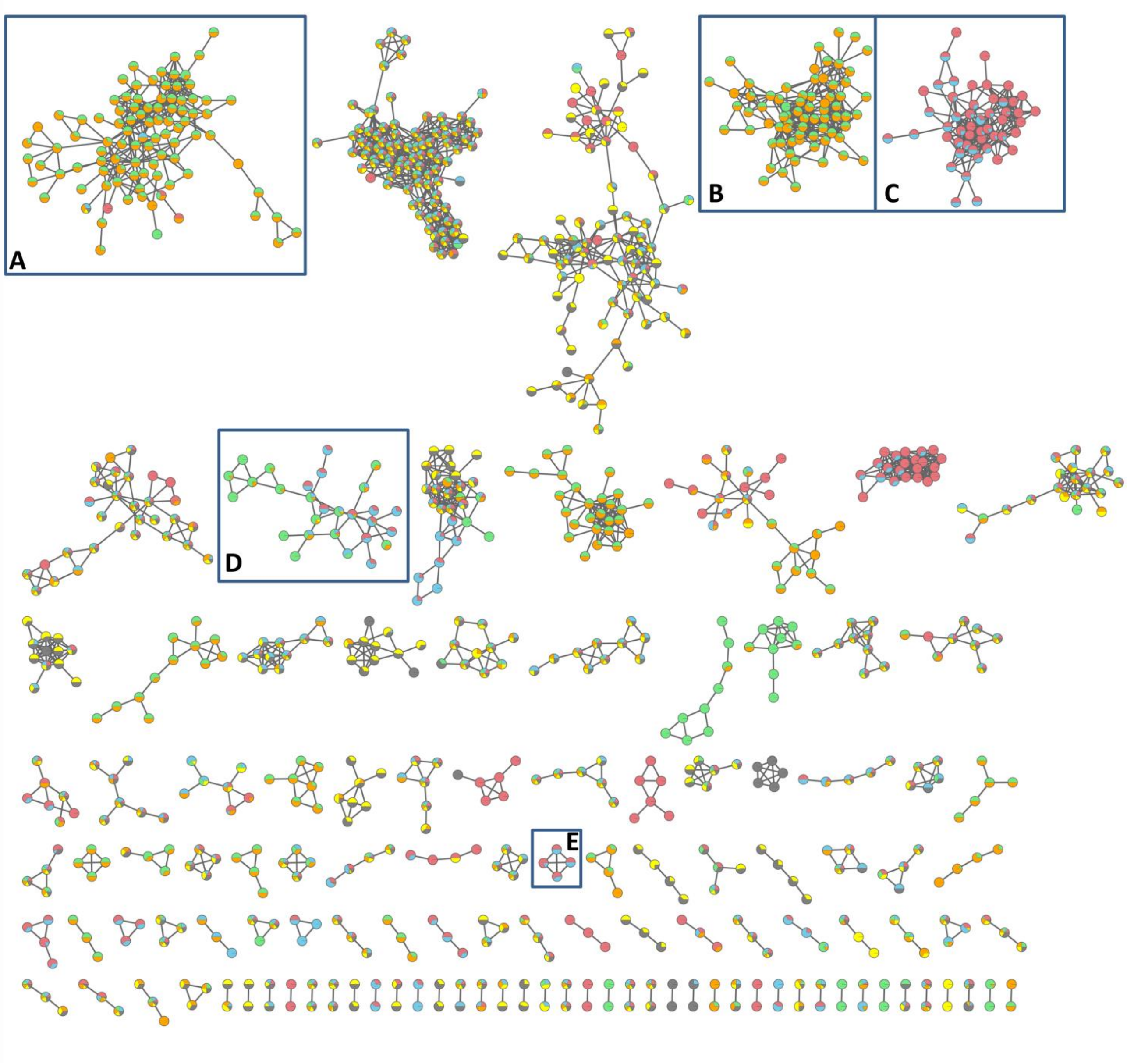

Figure S9. GNPS network of EtOAc extracts from six *Pseudoxylaria* spp. grown on PDA for two weeks. Colored nodes represent molecular ions from extracts of: Pseudoxylaria sp. OD126 (red), Pseudoxylaria sp. X802 (blue), *Pseudoxylaria* sp. X187 (green), *Pseudoxylaria* sp. Mn132 (orange), *Pseudoxylaria* sp. X3.2 (yellow) and *Pseudoxylaria* sp. X170LB (black). Identified metabolite clusters are represent as **A**) xylacremolides (X187/Mn132), **B**) pseudoxylariamides (X187/Mn132), **C**) pseudoxylallemcycins (X802/OD126), **D**) cytosporin/xylasporins (X802/OD126 and X187/Mn132**)** and **E**) cytochalasins (X802/OD126).

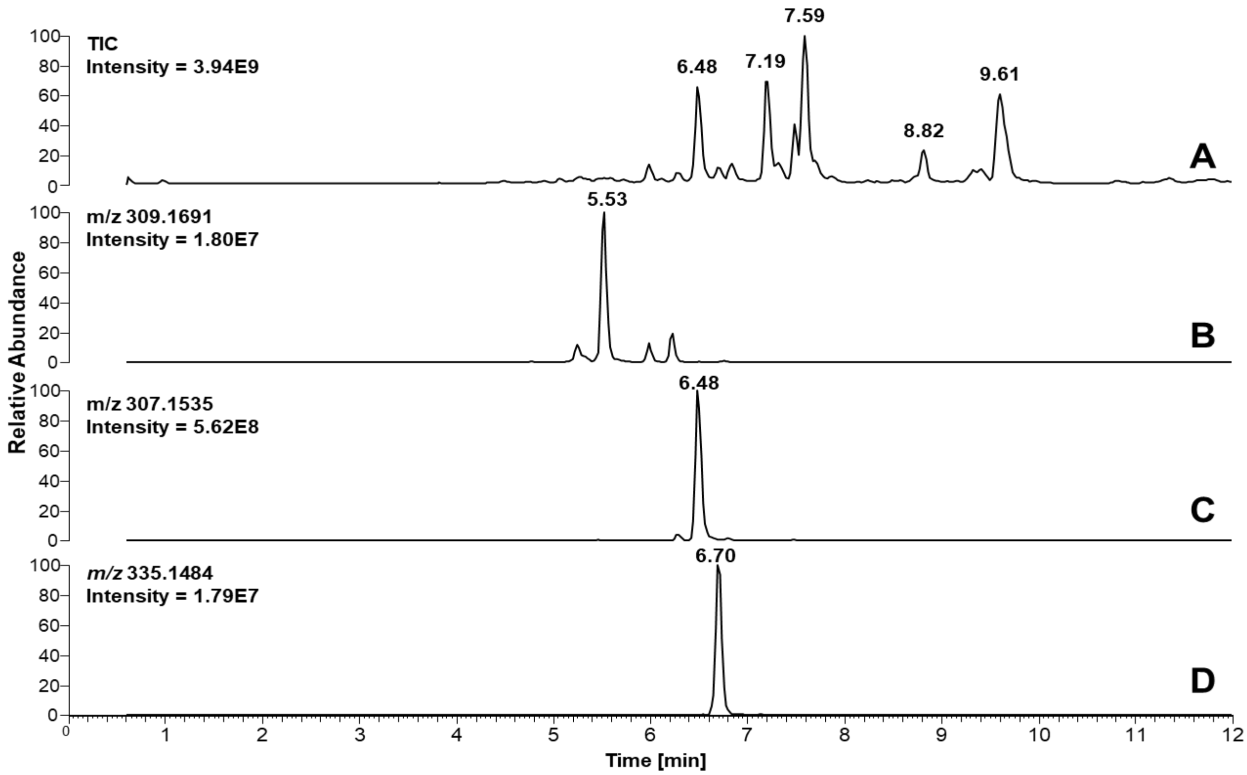

Figure S10. LC-HRMS chromatogram of *Pseudoxylaria* sp. X187 raw extract (EtOAc) with chromatogram traces **A**) TIC and XICs corresponding to novel compounds: **B**) xylasporin G, *m/z* 309.1691; **C**) xylasporin I, *m/z* 307.1535 and **D**) xylasporin H, *m/***z** 335.1484.

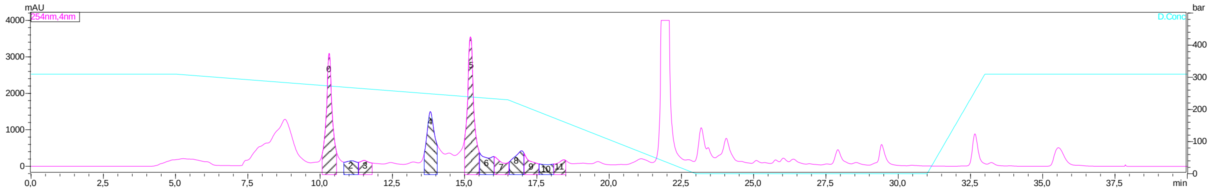

Time (min)	% Acetonitrile (A)	% H_2O + 0.1% FA(B)
0	38	62
5	38	62
16.5	54	46
23	100	0
31	100	0
33	38	62
40	38	62

Figure S11. Semipreparative HPLC chromatogram of culture extracts showing fractions of xylasporin I (Fraction 0) and xylasporin G (Fraction 4). Gradient of ACN and H_2O+ 0.1% FA is displayed in light blue.

Table S16. NMR assignment of xylasporin G and I based on HMBC and key ^{1}H-^{1}H COSY correlations ($CDCl_3$), 500 MHz.

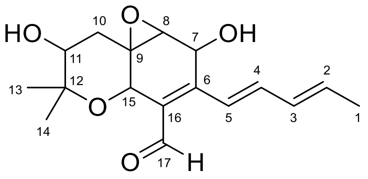

xylasporin G

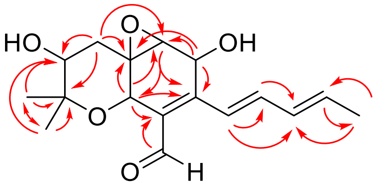

HMBC

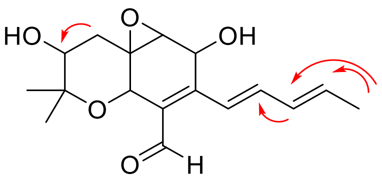

COSY

xylasporin G						xylasporin I	
**C#**	**δ ^{13}C**	**CH_x**	**δ ^{1}H, multiplicity**	**HMBC**	**^{1}H-^{1}H COSY**	**CH_x**	**δ ^{1}H, multiplicity**
1	18.8	CH_3	1.87, dd	137.6; 131.6	6.06; 6.24	CH_3	1.81, d
2	137.6	CH	6.06, m	18.8		CH	5.88, m
3	131.6	CH	6.24, m		6.83	CH	6.15, m
4	140.7	CH	6.83, m	131.6		CH	6.32, m
5	122.0	CH	6.86, m	131.6; 140.7		CH	6.62, m
6	149.3	C_q	---			C_q	---
7	69.3	CH	4.87, s	149.3; 58.9; 62.7; 127.5		CH	4.7, s
8	58.9	CH	3.50, s	149.3; 64.9		CH	3.50, s
9	62.7	C_q	---			C_q	---
10	29.7	CH_2	2.58, dd J=12.3, 9.8 Hz<br>1.95, dd J=12.4, 6.7 Hz	58.9; 62.7; 72.3; 83.2	4.03	CH_2	2.55, m; 1.97, m
11	83.2	CH	4.03, dd J=9.8, 6.7 Hz			CH	4.08, m
12	72.3	C_q	---			C_q	---
13	26.3	CH_3	1.25, s	83.2; 72.3		CH_3	1.26, s
14	23.9	CH_3	1.19, s	83.2; 72.3		CH_3	1.19, s
15	64.9	CH	4.82, s	149.3; 58.9; 62.7; 127.5		CH	4.6, s
16	127.5	C_q	---			C_q	---
17	191.3	CH	10.19, s	127.5		CH_2	4.28, 4.50
11-OH	---	OH	nd			OH	nd
7-OH	---	OH	nd			OH	nd
--	---	---	---			17-OH	nd

Structure elucidation of xylasporin G: The sum formula of xylasporin G was determined as $C_{17}H_{22}O_5$ by ESI-(+)-HRMS (calcd. for $[M+H]^+$ $C_{17}H_{23}O_5^+$ = 307.1540, found 307.15347, -1.726 ppm) with 7 degrees of unsaturation. An additional 18th signal [δ_C 162.7] in the ^{13}CNMR spectrum (likely formic acid) could be removed by re-measuring of xylasporin G in MeOH-*d3*. The ^{1}H-NMR spectrum of xylasporin G revealed the presence of an aldehyde group [H-17, δ_H 10.19, δ_C 191.3] and three methyl groups based on chemical shift and integration [H-1 H-13 H-14, δ_H 1.87 1.25 1.19, δ_C 18.8 26.3 23.9] as well as four protons located on double bonds [H-2 H-3 H-4 H-5, δ_H 6.06 6.24 6.83 6.86, δ_C 137.6 131.6 140.7 122.0]. The DEPT135 spectra revealed the presence of only a single CH_2 unit in the molecule [H-10, δ_H 1.96 2.56, δ_C 29.7]. Combination of ^{13}C-NMR and DEPT135 revealed the presence of another double bond built from two quaternary carbons [C-6 C-16, δ_C 149.3 127.5]. The structure of the sidechain typical for xylasporins was deduced by ^{1}H-^{1}H COSY correlations between H-1/H-2 [δ_H 1.87 and 6.06], H-1/H-3 [δ_H 1.87 and 6.24] and H-3/H-4 [δ_H 6.83 and 6.86]. This connection is also supported by respective HMBC correlations between H-1/C-2 [δ_H 1.87, δ_C 137.6], H-1/C-3 [δ_H 1.87, δ_C 131.6], H-5/C-4 [δ_H 6.86, δ_C 140.7], H-5/C-3 [δ_H 6.86, δ_C 131.6] and H-4/C-3 [δ_H 6.83, δ_C 131.6]. The aldehyde group was located by HMBC correlations next to quaternary carbon C-16 [δ_H 10.19, δ_C 127.5]. The remaining two methyl groups C-13 and C-14 are connected to the same quaternary carbon C-12 by HMBC correlations [δ_H 1.25 δ_H 1.19, δ_C 72.3] which also carries an oxygen, based on its chemical shift. C-12 was located next to C-11 by additional weaker HMBC correlations of H-13/C-11 and H-14/C-11 [δ_H 1.25 δ_H 1.19, δ_C 83.2]. C-11 carries oxygen based on chemical shift and was placed next to the only CH_2 group (C-10). Both protons H-10_1 H-10_2 give HMBC signals back to C-11 and C-12 [δ_H 2.58 δ_H 1.95, δ_C 83.2 δ_C 72.3] as well as towards quaternary carbon C-9 [δ_H 2.58 δ_H 1.95, δ_C 62.7]. Complex mirrored HMBC 2-3 bond correlations between the four oxygenated carbons C-7, C-8, C-9 and C-15 and the related protons support the assumption of a ring structure which fits well into the predicted core structure of xylasporin (GNPS, BigScape). (H-15/C-9 [δ_H 4.82, δ_C 62.7], H-15/C-8 [δ_H 4.82, δ_C 58.9], H-15/C-16 [δ_H 4.82, δ_C 127.5], H-15/C-6 [δ_H 4.82, δ_C 149.3] and H-7/C-6 [δ_H 4.87, δ_C 149.3], H-7/C-8 [δ_H 4.87, δ_C 58.9], H-7/C-9 [δ_H 4.87, δ_C 62.7] as well as H-8/C-6 [δ_H 3.50, δ_C 149.3], H-8/C-15 [δ_H 3.50, δ_C 64.9]).

Structure elucidation of xylasporin I: The sum formula of xylasporin I was determined as $C_{17}H_{24}O_5$ by ESI-(+)-HRMS (calcd. for $[M+H]^+$ $C_{17}H_{25}O_5^+$ = 309.1697, found 309.1691, -1.68 ppm) with six degrees of unsaturation. Due to the chemical instability of xylasporin I structural analysis by NMR was limited. Based on the recorded HRMS2-pattern and characteristic ^{1}H signals the planar structure could be deduced. In addition to very similar 1H pattern, the absence of an aldehyde proton signal [307: δ_H 10.19] was characteristic, which was supported by the absence of deep field shifted aldehyde carbon [307: δ_C 191.3]. Instead the ^{1}H NMR showed additional proton signals caused by the new H-17 located at the $-CH_2OH$ group within the area between 4 and 5 ppm.

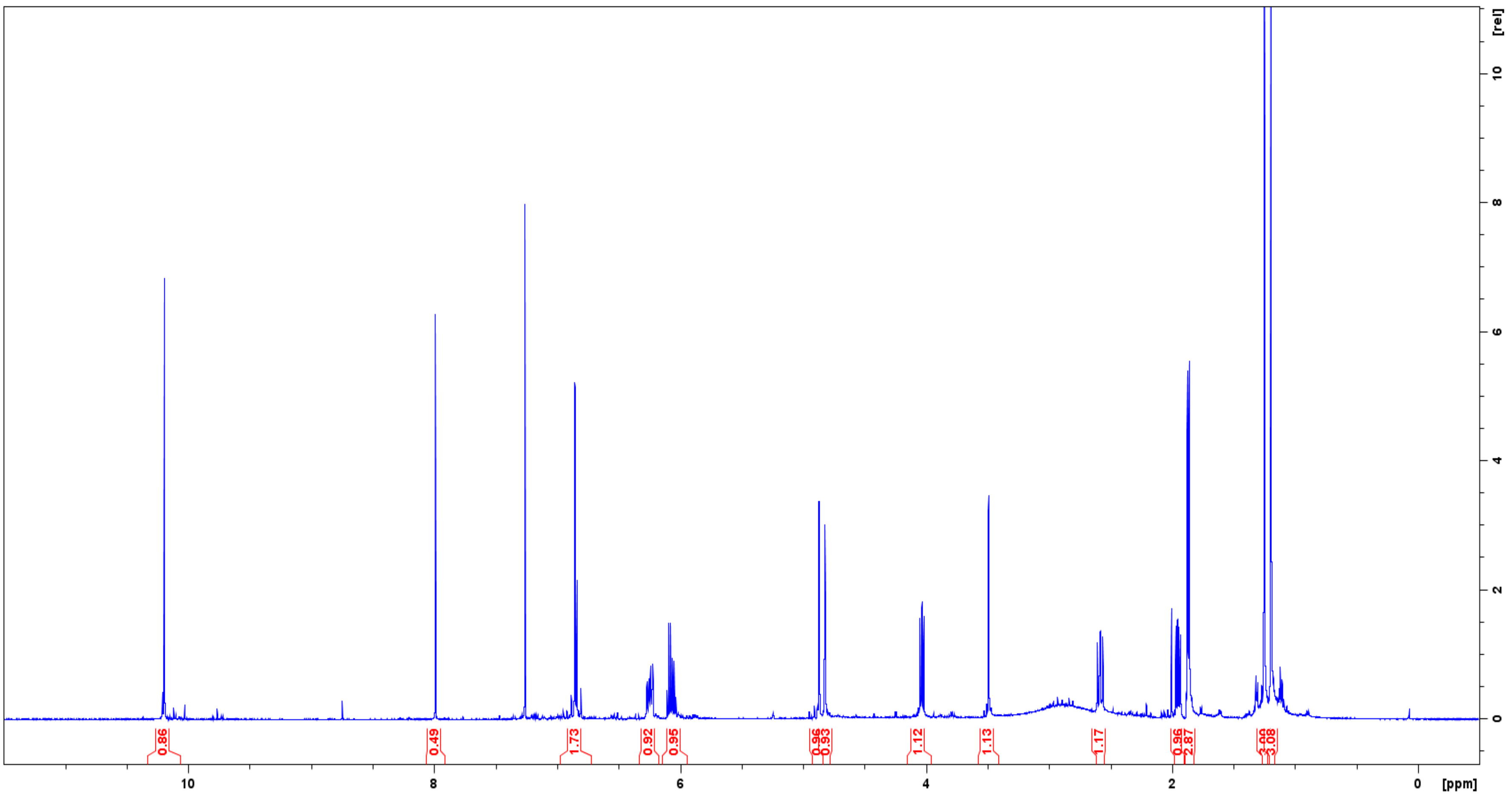

Figure S12. Integrated ^{1}H-NMR spectrum of xylasporin G in $CDCl_3$, 500 Mhz.

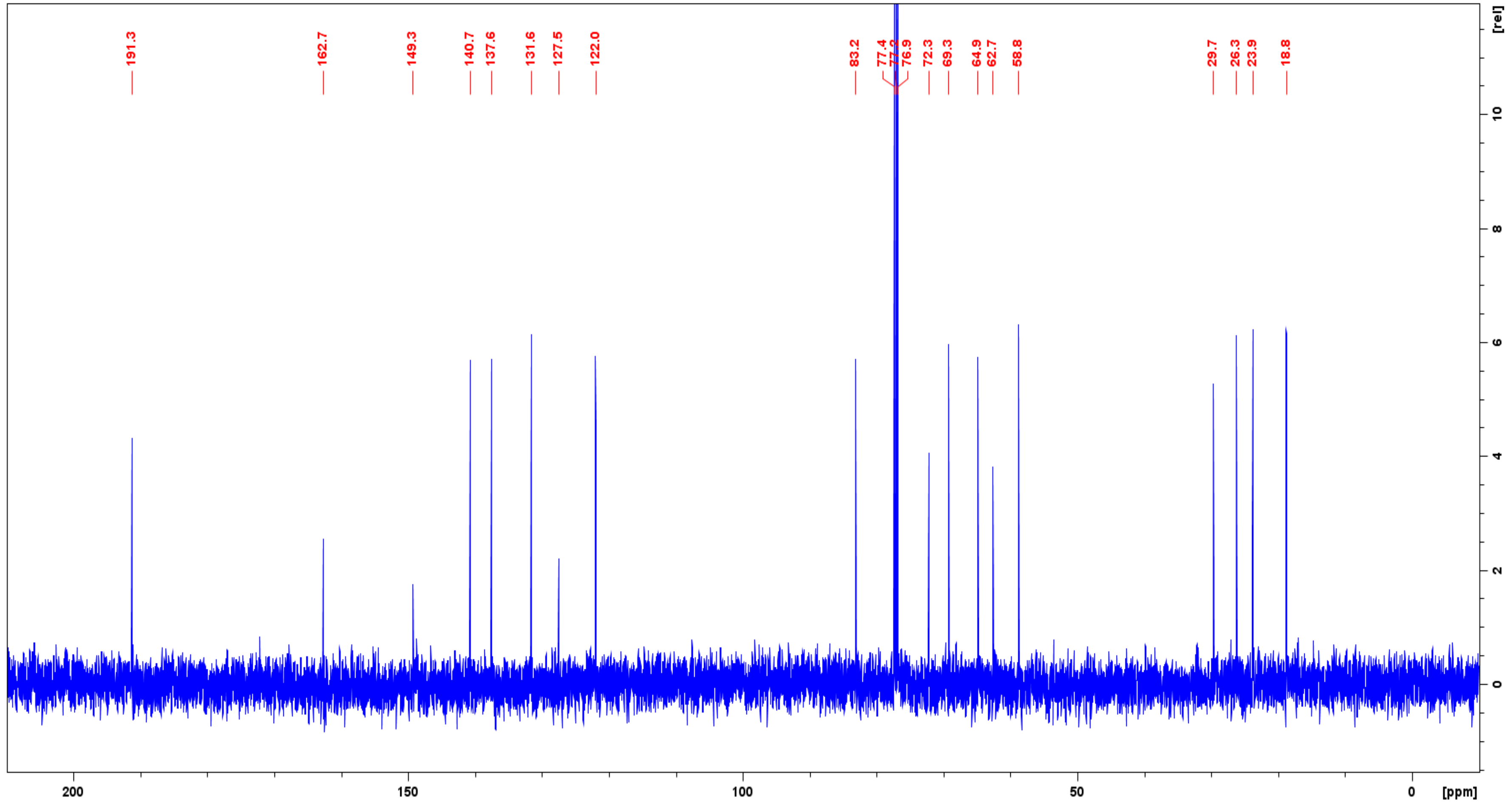

Figure S13. Peak-picked ^{13}C-NMR spectrum of xylasporin G in $CDCl_3$, 500 Mhz.

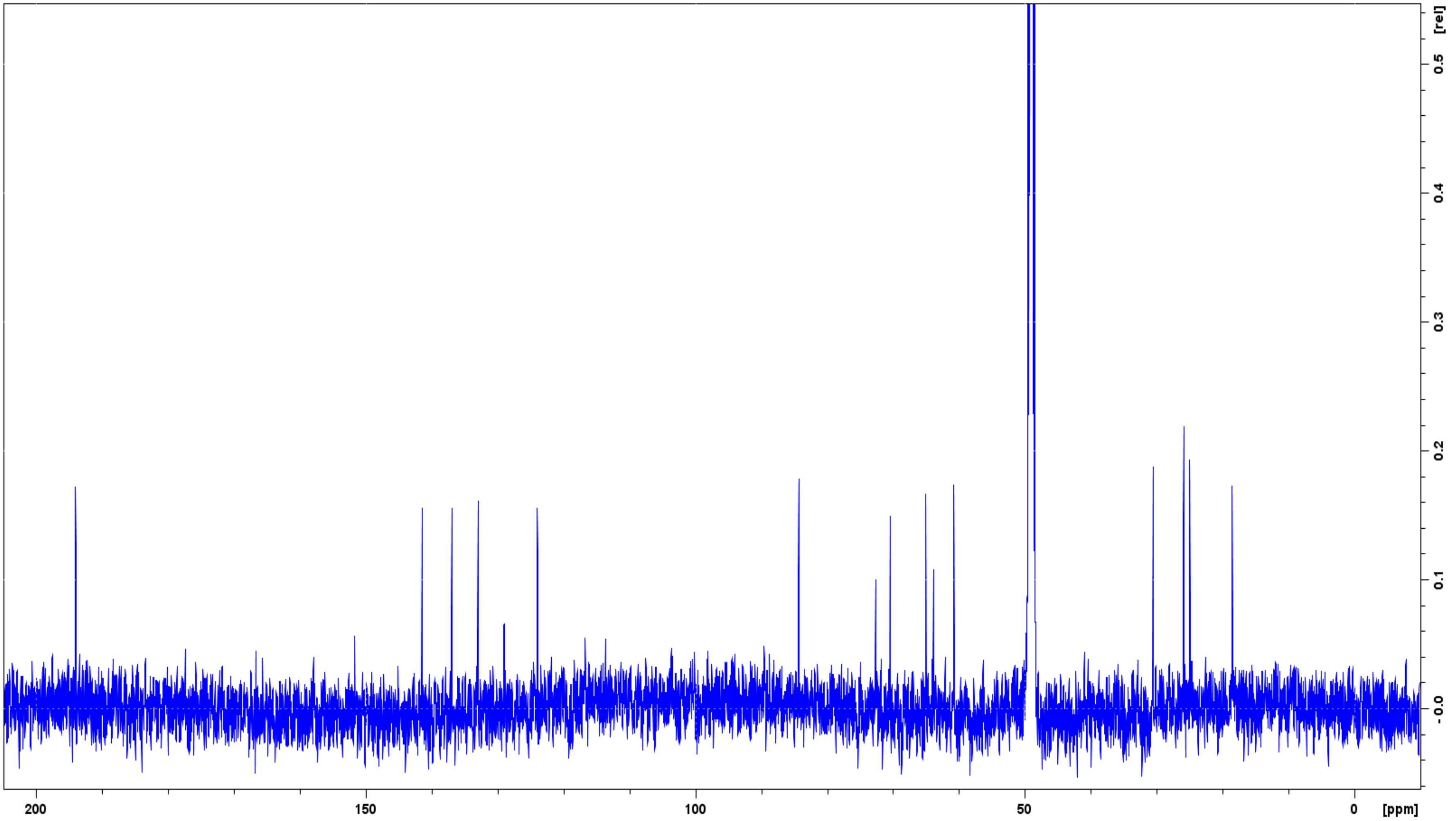

Figure S14. ^{13}C-NMR spectrum of xylasporin G in MeOH-*d3*, 500 Mhz.

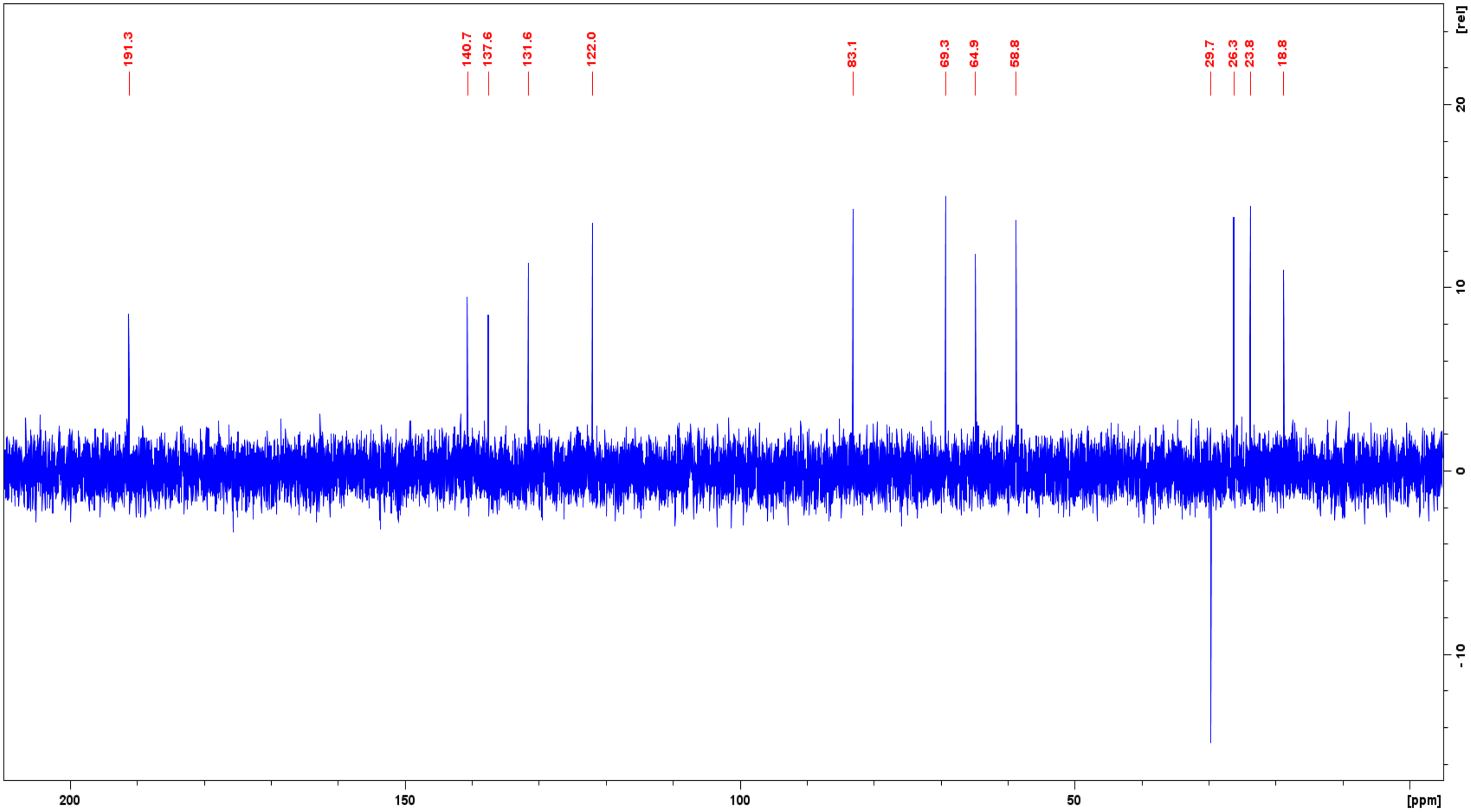

Figure S15. Peak-picked DEPT135 spectrum of xylasporin G in $CDCl_3$, 500 Mhz.

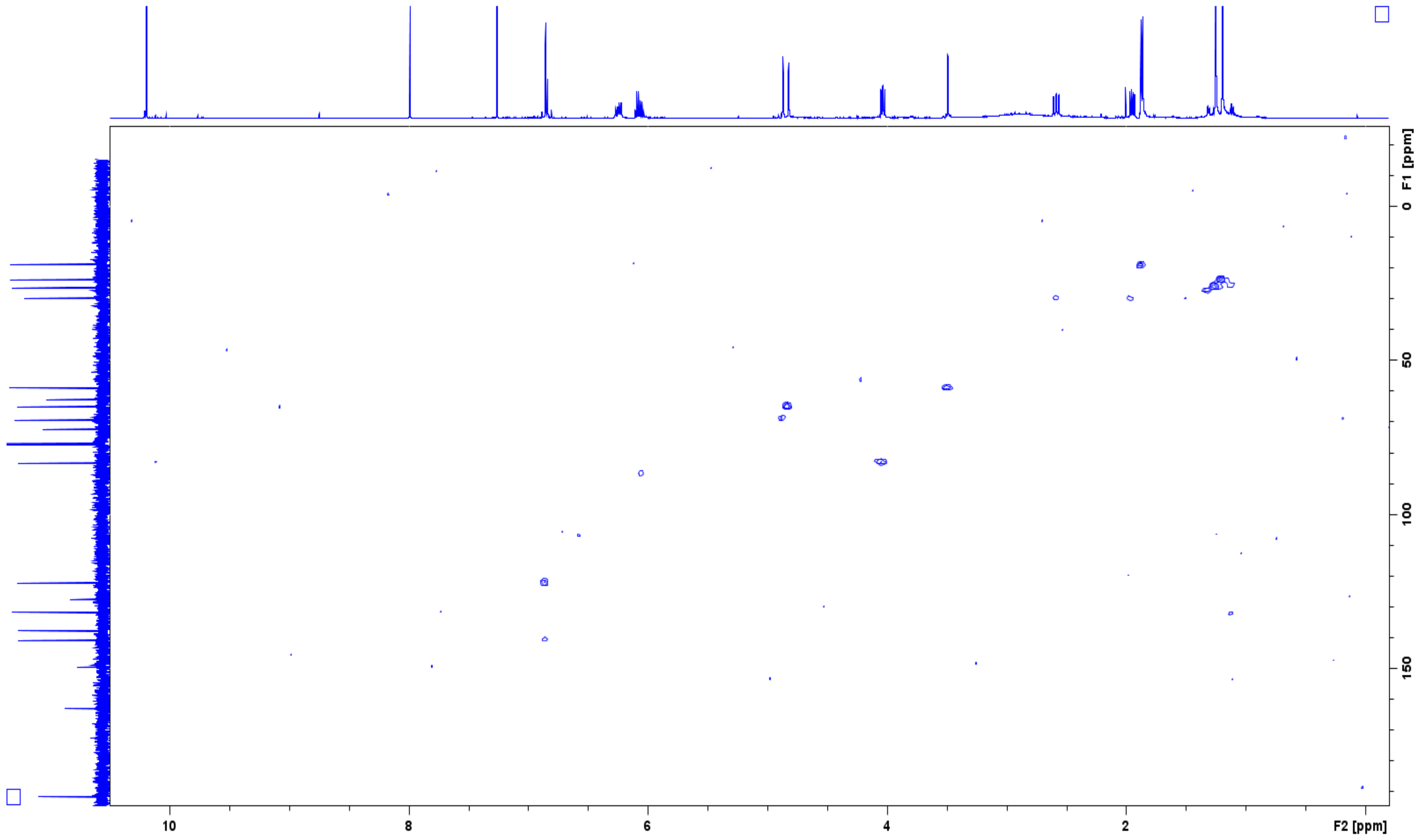

Figure S16. HSQC spectrum of xylasporin G in $CDCl_3$, 500 Mhz.

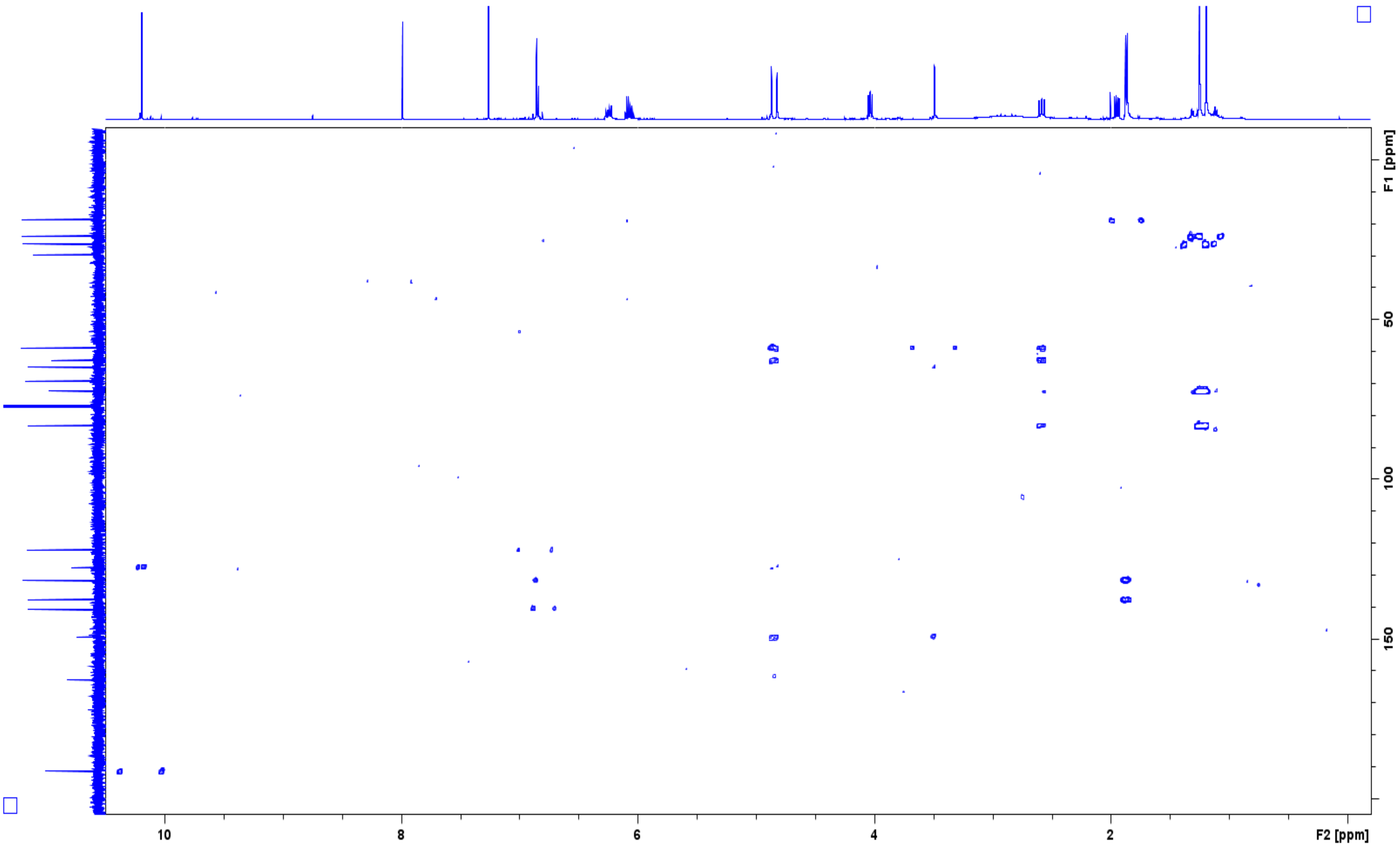

Figure S17. HMBC spectrum of xylasporin G in $CDCl_3$, 500 Mhz.

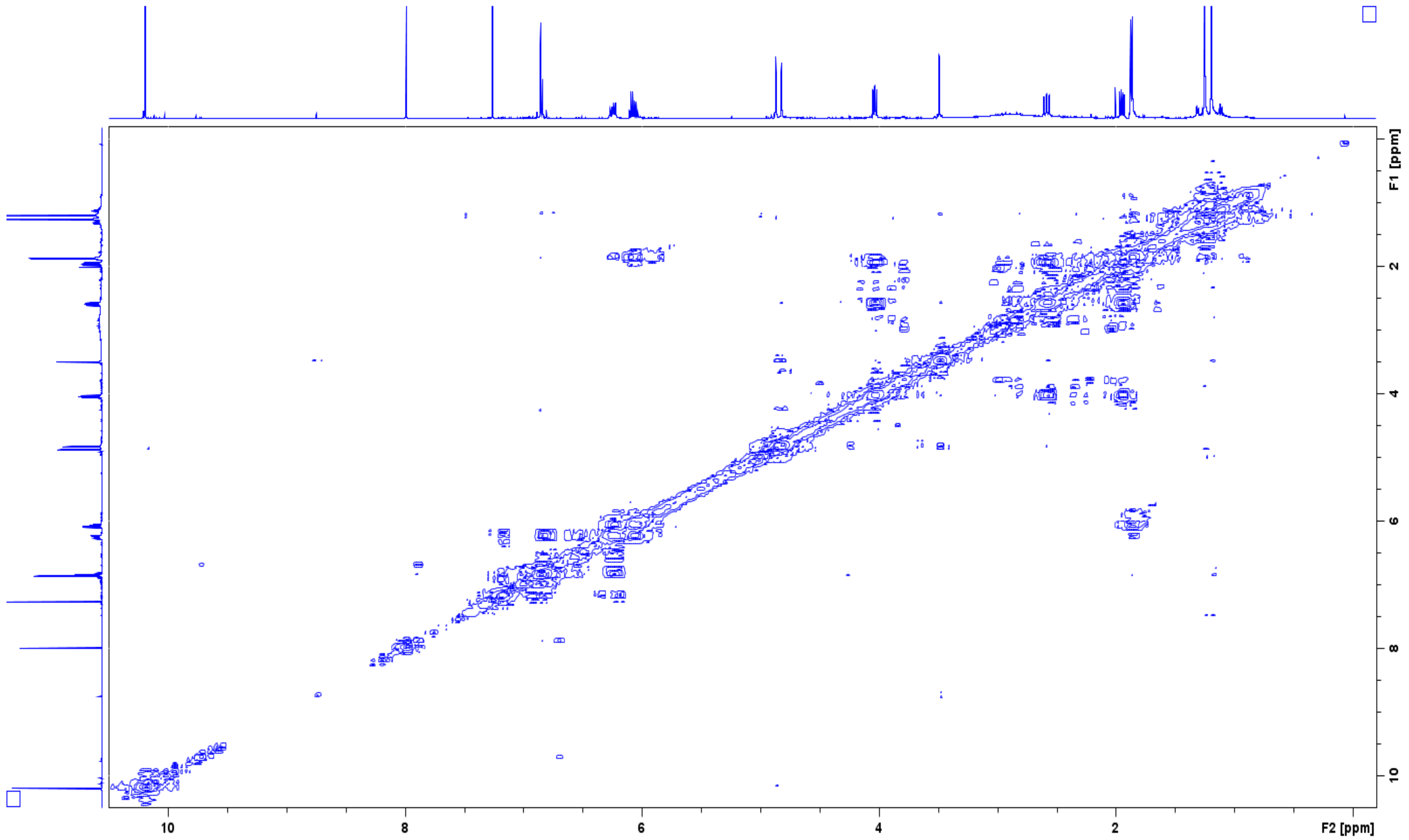

Figure S18. COSY spectrum of xylasporin G in $CDCl_3$, 500 Mhz.

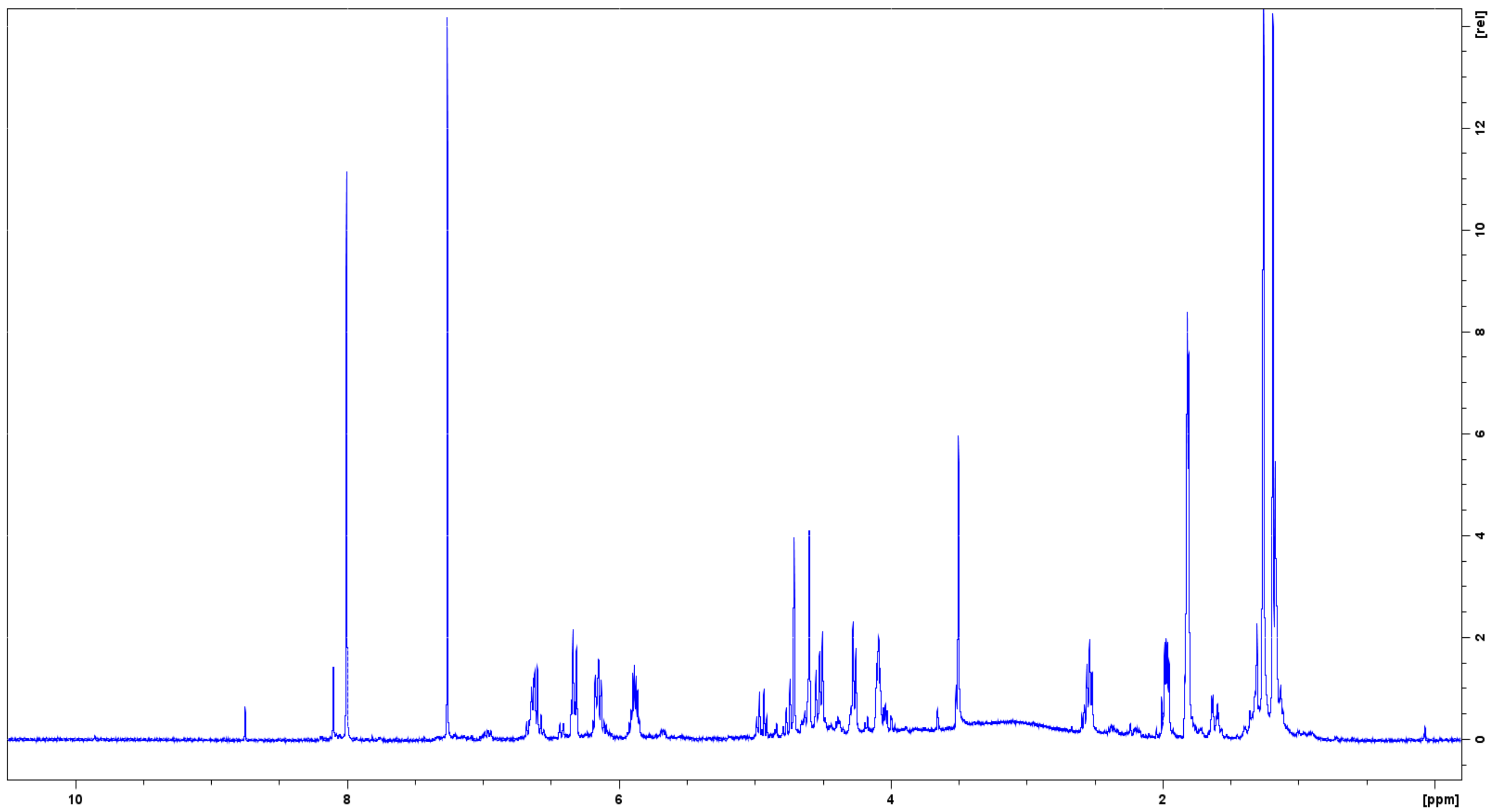

Figure S19. ^{1}H-NMR of xylasporin I in $CDCl_3$, 500 Mhz.

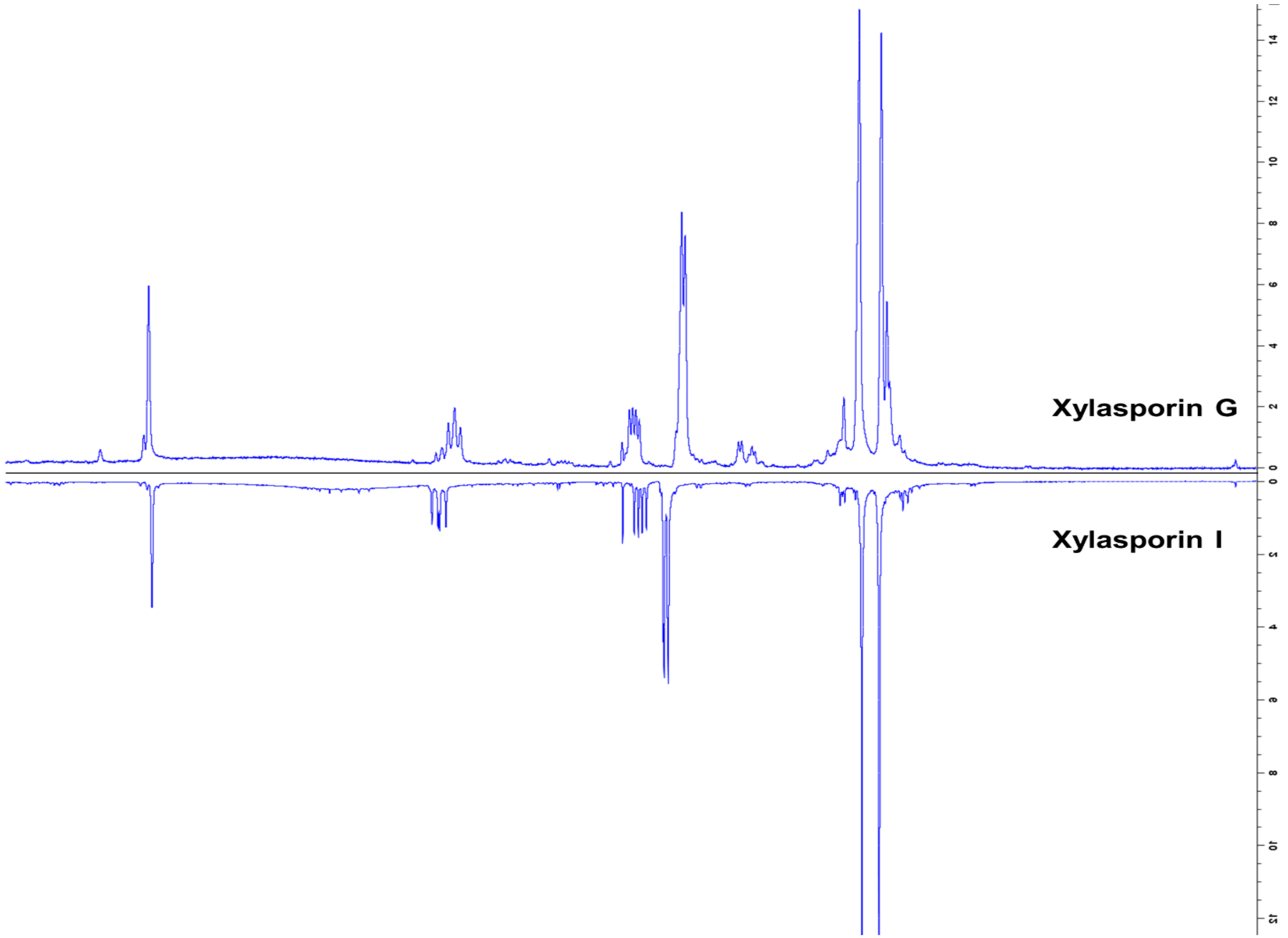

Figure S20. Comparison of ^{1}H NMR spectra ($CDCl_3$, 500 Mhz) for xylasporin G (top) and xylasporin I (inverted, bottom). Spectra are inverted based on the ppm axis and are displayed from 0 to 4 ppm.

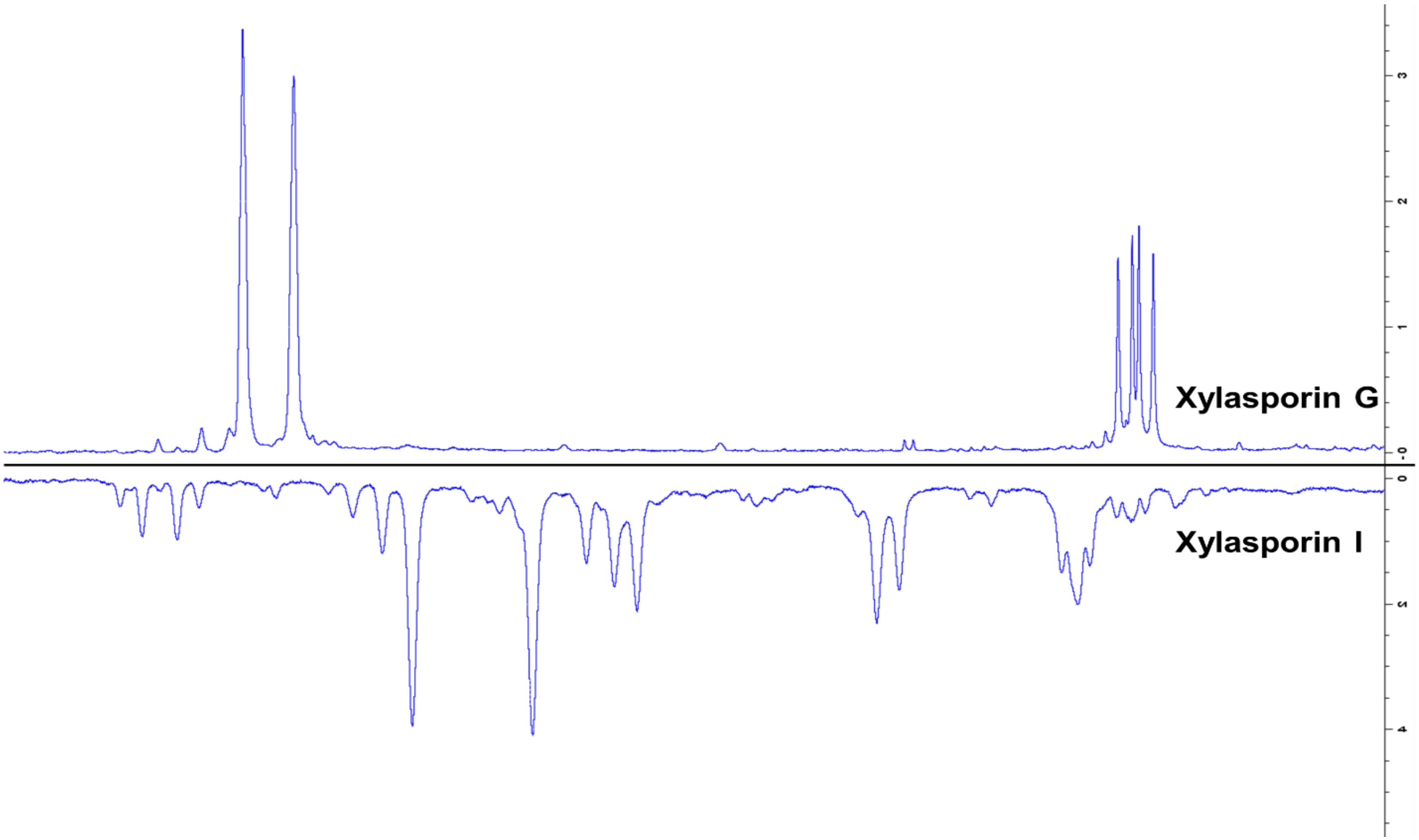

Figure S21. Comparison of ^{1}H NMR spectra ($CDCl_3$, 500 Mhz) for xylasporin G (top) and xylasporin I (inverted, bottom). Spectra are inverted based on the ppm axis and are displayed from 3.8 to 5.1 ppm.

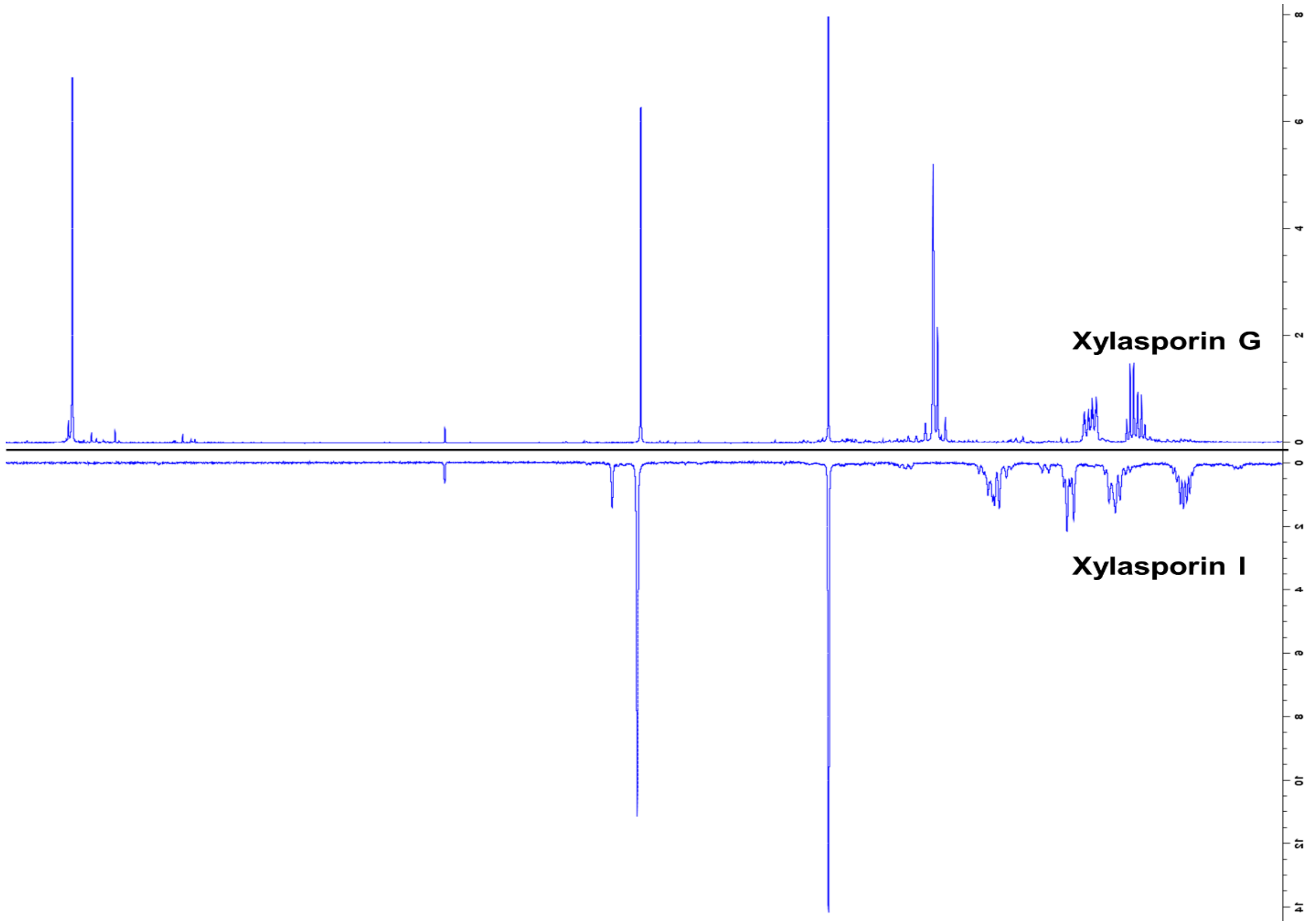

Figure S22. Comparison of ^{1}H NMR spectra for xylasporin G (top side) and xylasporin I (inverted, bottom). Spectra are inverted based on the ppm axis and are displayed from 5.5 to 10.5 ppm. $CDCl_3$, 500 Mhz.

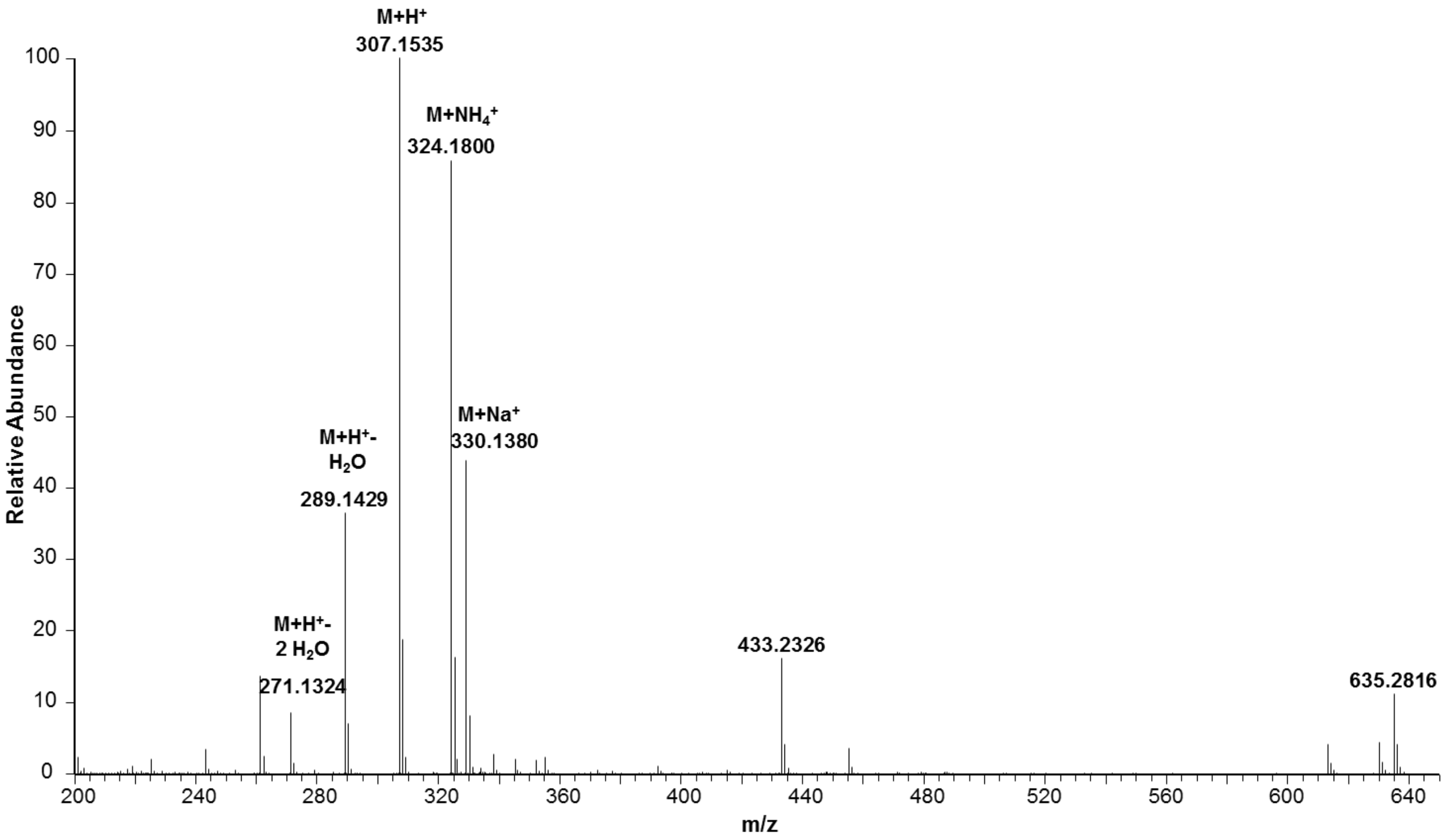

Figure S23. ESI(+)-HRMS spectrum of xylasporin G. A strong double water loss and adduct formation, *m/z* $[M+H]^+$ = 307.15347, $C_{17}H_{23}O_5^+$ calcd. -1.726 ppm. RT = 6.48 min was observed.

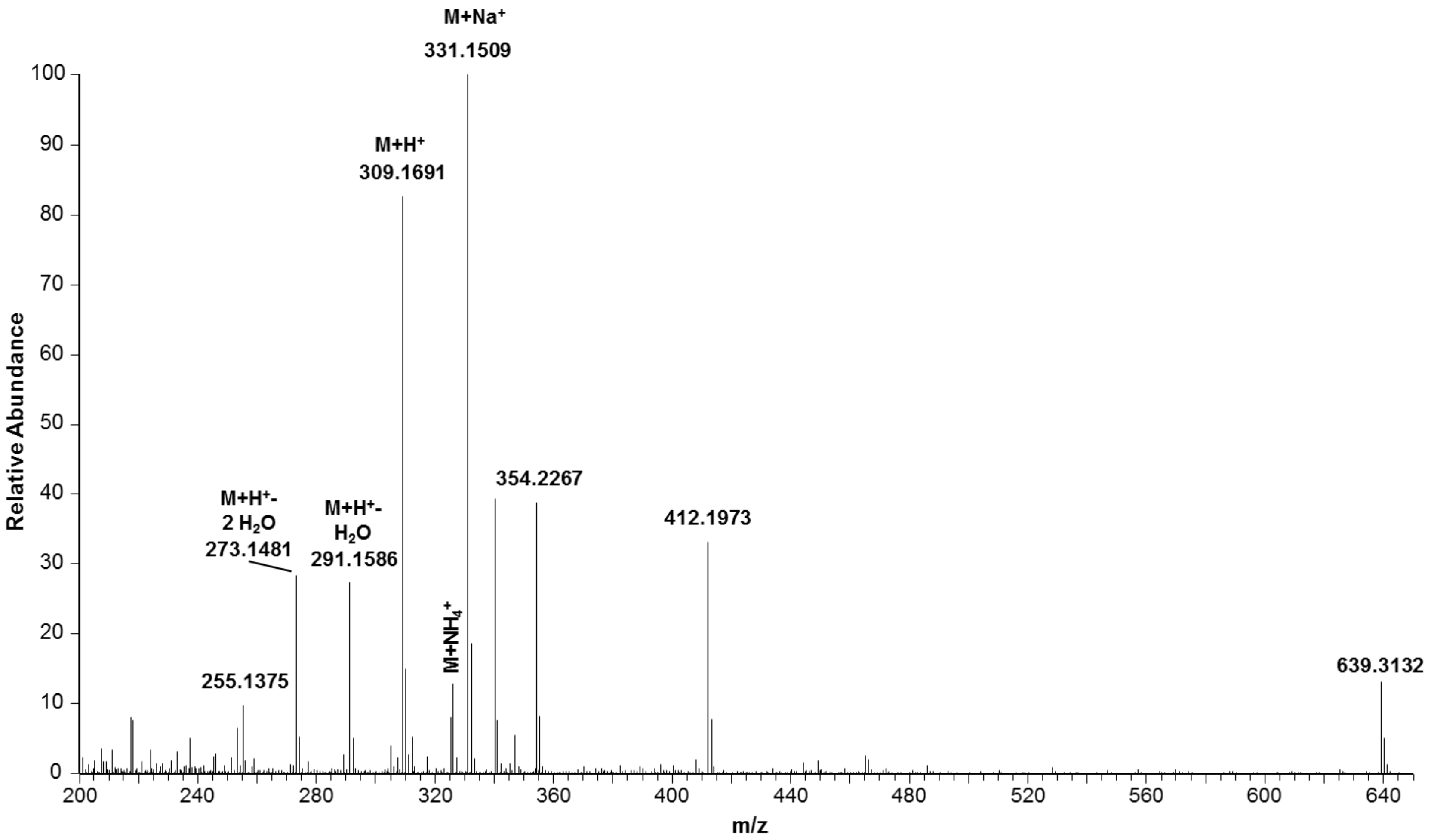

Figure S24. ESI(+)-HRMS spectrum of xylasporin I. A strong double water loss and adduct formation, *m/z* $[M+H]^+$ = 309.16910, $C_{17}H_{25}O_5^+$ calcd. -1.683 ppm. RT = 5.53 min was observed.

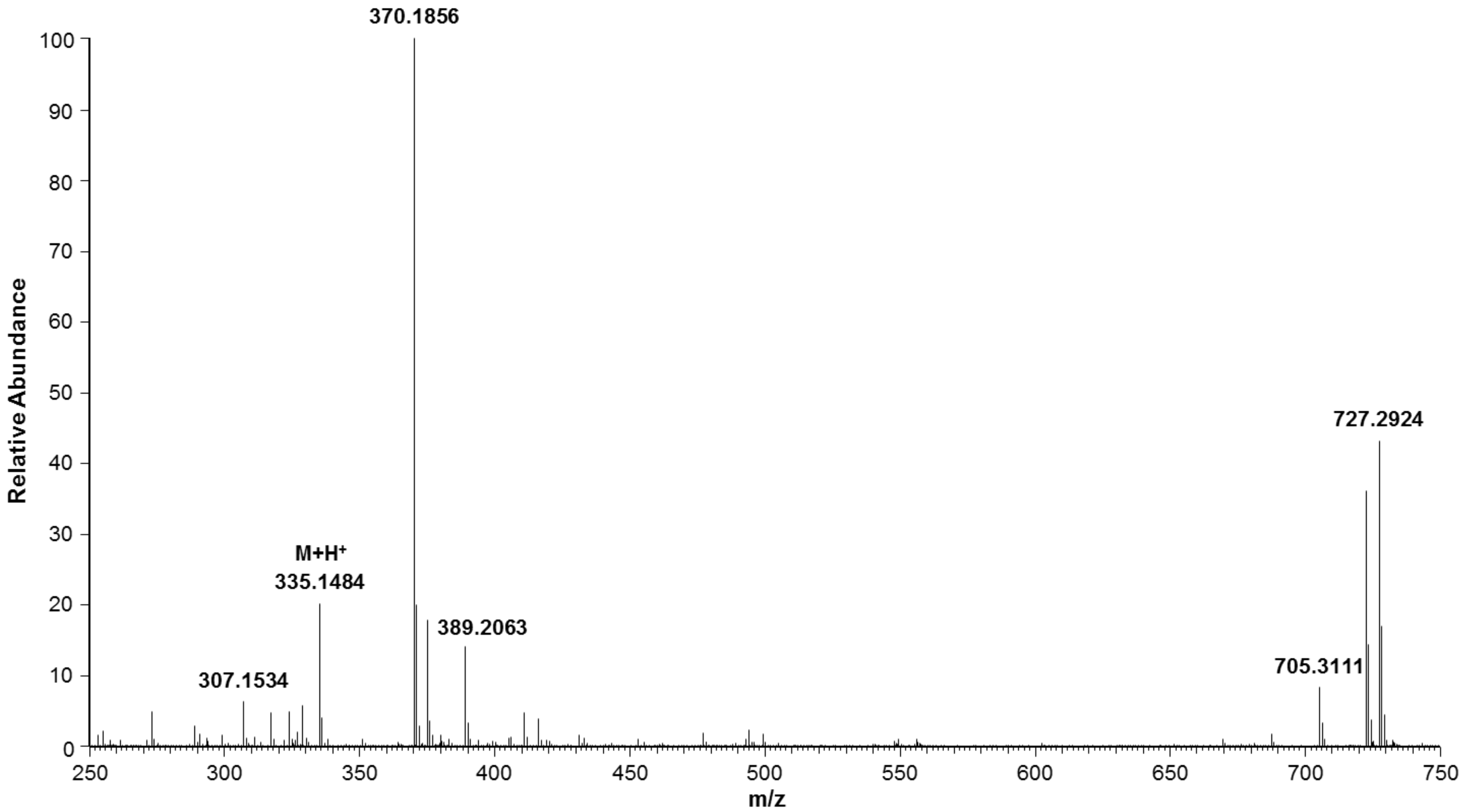

Figure S25. ESI(+)-HRMS spectrum of hypothesized xylasporin H carbonate (*m/z* $[M+H]^+$ = 335.1484, $C_{18}H_{23}O_6^+$ calcd. 1.417 ppm. RT = 6.70 min).

Table S17. Antimicrobial activity of *Pseudoxylaria* sp. extracts was determined by measuring the inhibition zone (ZOI) in mm ((p) colonies inside inhibition zone, (P) many colonies inside inhibition zone, (A) visible hint indicating potential inhibition)

Isolate	*B. subtilis*	*S. aureus*	*E. coli*	*P. aeruginosa*	*M. vaccae*	*S. salmonica* 549	*C. albicancs*	*P. notatum*
X802 crude	10	0	0	0/A	0	0	0	0
X802 (100%)	12	11	0	0	24p	0	0	0
X802 (50%)	10	0	0	0	0	0	0	0
OD126 crude	10	0	0	0/A	0	0	0	0
OD126 (100%)	11	10	0	0	19p	0	0	0
OD126 (50%)	0	0	0	0	0	0	0	0
X187 crude	0	0	0	0	0	0	0	0
X187 (100%)	12	12	0	0	27p	0	0	0
X187-2 (50%)	0	0	0	0	0	0	0	0
X3-2 crude	10	0	0	0/A	21p	0	0	0
X3-2 (50%)	0	0	0	0	0	0	0	0
X170 LB	0	0	0	0/A	12p	0	0	0
X170 LB (50%)	0	0	0	0	0	0	0	0
X167 crude	10	0	0	0/A	24p	0	0	0
X167 (50%)	0	0	0	0	0	0	0	0
MN153 crude	25.5p	18.5	0	20p	36	0	0	0
Cip	29	18	28.5	33.5	21p	-	-	-
MeOH	0	0	0	10	0	10	0	11
Amphotericin B	-	-	-	-	-	19p	21	18p

Table S18. Disc diffusion assay against *Termitoymces* sp. 153 using cytochalasines and *Pseudoxylaria* sp. extracts.

Test substance	N = 1	N = 2
cytochalasines	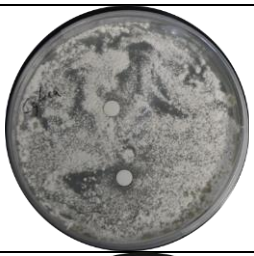	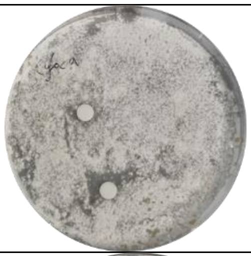
Extracts of X802	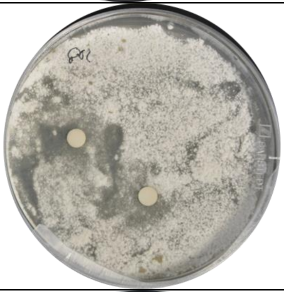	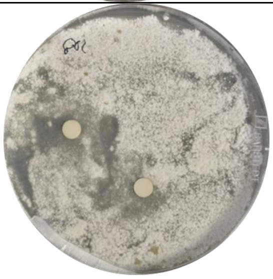
Extracts of X187	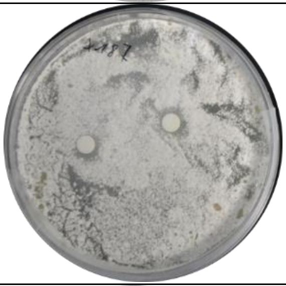	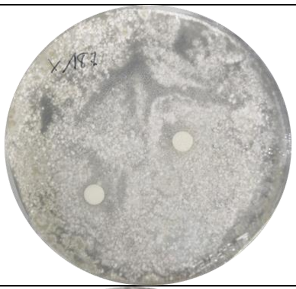
Extracts of MN132	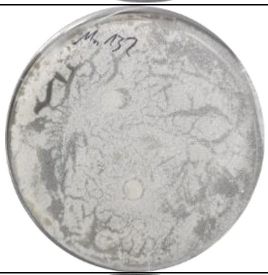	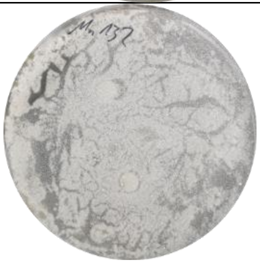
Extracts of X170LB	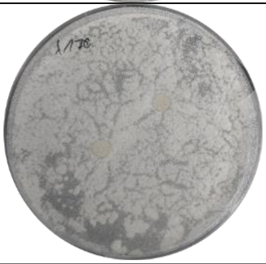	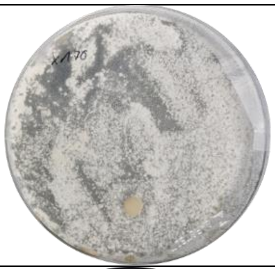
Extracts of X3.2	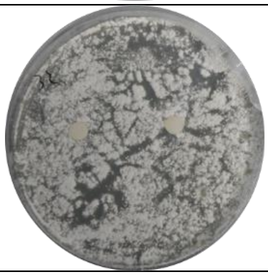	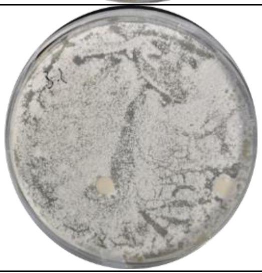

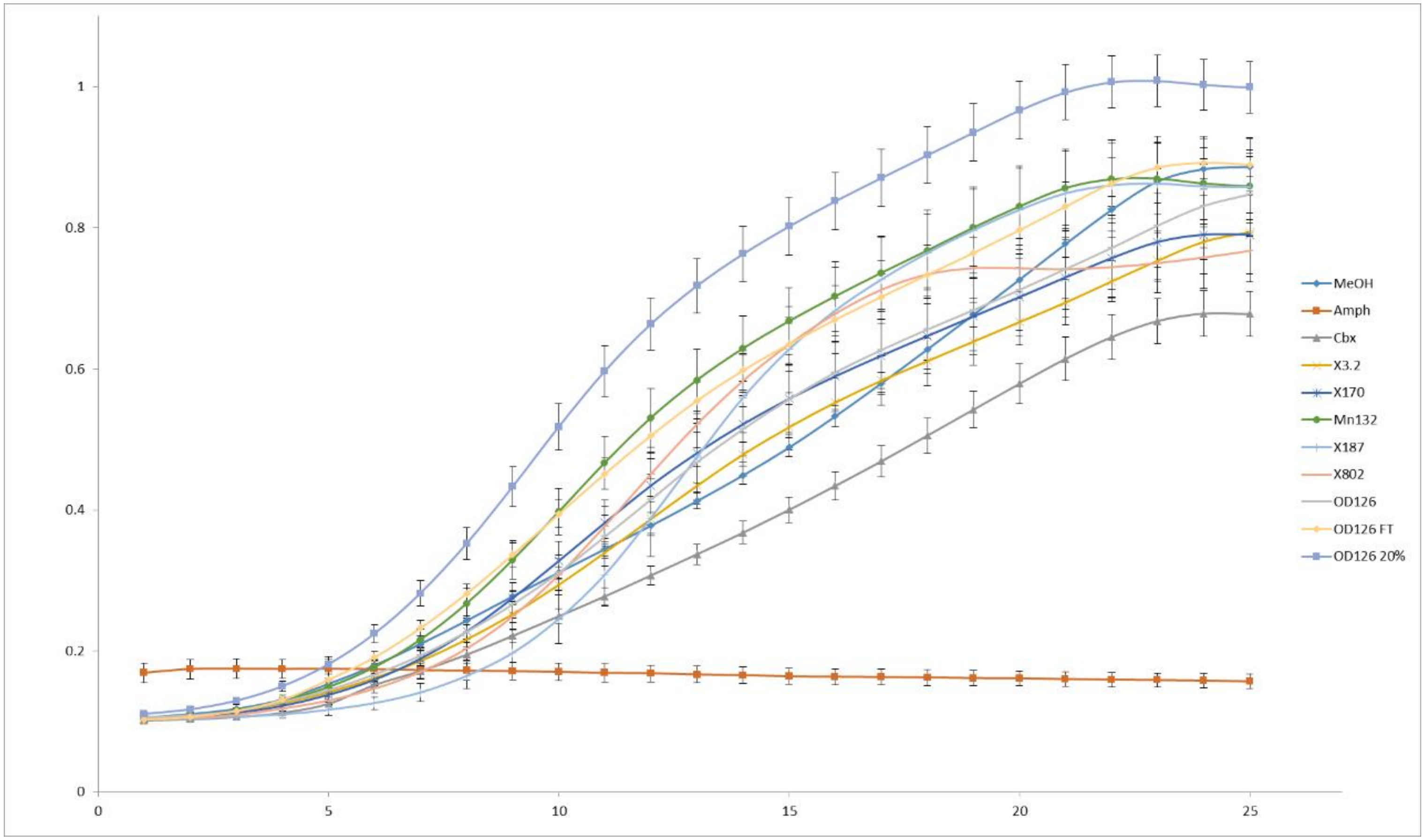

Figure S26. Broth dilution assay of *Pseudoxylaria* sp. extracts against *Saccharomyces cerevisiae* BY4741.

Table S19. Feeding studies investigating the effect of fungus consumption on the relative growth rate (RGR) of *S. littoralis* caterpillars. Insects were fed with PDA (A), *Termitomyces* sp. T153 (B) or *Pseudoxylaria* sp. X802 (D) growing on PDA and PDA after removing fungal mycelium of the latter fungi, respectively (C, E). All experiments were performed with 25 replicates per treatment, a duration of 10 days, and larval weights and survival rates were recorded every day.

Treatment	Sample size (N) at the end of the	RGR	p-value (ANOVA)
A	25	0.084 ± 0.002	
B	25	0.185 ± 0.0004	
C	20	0.116 ± 0.004	< 0.001
D	6	0.065 ± 0.009	
E	0	N/A	
Pairwise Multiple Comparison Procedure (Dunn’s Method):			
B vs. D	p < 0.05		
B vs. C	p < 0.05		
C vs. D	p < 0.05		
A vs. B	p < 0.05		
A vs. C	p < 0.05		
A vs. D	p > 0.05		

Table S20. Results of additional insect feeding studies. All experiments were performed with 25 replicates per treatment, a duration of 10 days, and larval weights and survival rates were recorded every day.

Technical replicates (Date	Treatment	Final sample size at the end	RGR	p-value
T153_R1 (181107)	A	25	0.093 ± 0.003	< 0.001
	B	25	0.173 ± 0.001	
	C	22	0.073 ± 0.003	
T153_R2 (181205)	A	22	0.094 ± 0.004	< 0.001
	B	22	0.169 ± 0.001	
	C	15	0.065 ± 0.005	
X802_R1 (180904)	A	24	0.095 ± 0.002	0.010
	B	3	0.131 ± 0.013	
	C	0	N/A	
X802_R2 (181005)	A	22	0.109 ± 0.001	N/A
	B	0	N/A	
	C	0	N/A	

References

[1] Sutheeworapong S, Suteerapongpan N, Paenkaew P, et al (2019) Draft Genome Sequence of the Wood-Decaying Fungus *Xylaria* sp. BCC 1067. Microbiol Resour Announc 8:1–2. https://doi.org/10.1128/MRA.00512-19

[2] Sharma S, Zaccaron AZ, Ridenour JB, et al (2018) Draft genome sequence of *Xylaria* sp., the causal agent of taproot decline of soybean in the southern United States. Data Br 17:129–133. https://doi.org/10.1016/j.dib.2017.12.060

[3] Mead ME, Raja HA, Steenwyk JL, et al (2019) Draft Genome Sequence of the Griseofulvin-Producing Fungus *Xylaria flabelliformis* Strain G536. Microbiol Resour Announc 8:14–16. https://doi.org/10.1128/MRA.00890-19

[4] Park SY, Jeon J, Kim JA, et al (2021) Draft Genome Sequence of Xylaria grammica EL000614, a Strain Producing Grammicin, a Potent Nematicidal Compound. Mycobiology 49:294–296. https://doi.org/10.1080/12298093.2021.1914360

[5] Wibberg D, Stadler M, Lambert C, et al (2020) High quality genome sequences of thirteen Hypoxylaceae (Ascomycota) strengthen the phylogenetic family backbone and enable the discovery of new taxa. Fungal Divers. https://doi.org/10.1007/s13225-020-00447-5

[6] Büttner E, Liers C, Hofrichter M, et al (2019b) Draft Genome Sequence of Xylaria hypoxylon DSM 108379, a Ubiquitous Fungus on Hardwood. Microbiol Resour Announc 8:9–11. https://doi.org/10.1128/mra.00845-19

[7] Büttner E, Gebauer AM, Hofrichter M, et al (2019a) Draft Genome Sequence of Xylaria longipes DSM 107183, a Saprotrophic Ascomycete Colonizing Hardwood. Microbiol Resour Announc 8:11–12. https://doi.org/10.1128/MRA.00157-19

[8] Büttner E, Liers C, Richter A, et al (2020) Draft Genome Sequence of the Ascomycete Xylaria multiplex DSM 110363. Microbiol Resour Announc 9:9–11. https://doi.org/10.1128/mra.00262-20

[9] Zharkikh, A., Estimation of evolutionary distances between nucleotide sequences. Journal of Molecular Evolution 1994, 39 (3), 315-329.

[10] Yang, Z., Maximum likelihood phylogenetic estimation from DNA sequences with variable rates over sites: Approximate methods. J. Mol. Evol. 1994, 39 (3), 306-314.

[11] Darling, A. C. E., Mau, B., Blattner, F. R., & Perna, N. T. Mauve: Multiple alignment of conserved genomic sequence with rearrangements. Genome Research 2004, 14:1394–1403. https://doi.org/10.1101/gr.2289704

[12] Deng Y, Hsiang T, Li S, Lin L, Wang Q, Chen Q, Xie B, Ming R. Comparison of the Mitochondrial Genome Sequences of Six *Annulohypoxylon stygium* Isolates Suggests Short Fragment Insertions as a Potential Factor Leading to Larger Genomic Size. Front Microbiol. 2018; 9:2079. doi: 10.3389/fmicb.2018.02079.

[13] Tang D, Zhang G, Wang Y, Zhang M, Wang Y, Yu H. Characterization of complete mitochondrial genome of *Nemania diffusa* (Xylariaceae, Xylariales) and its phylogenetic analysis. Mitochondrial DNA B Resour. 2020; 5:459-460. doi: 10.1080/23802359.2019.1704665.

[14] Zhang S, Wang XN, Zhang XL, Liu XZ, Zhang YJ. Complete mitochondrial genome of the endophytic fungus Pestalotiopsis fici: features and evolution. Appl Microbiol Biotechnol. 2017; 101:1593-1604. doi: 10.1007/s00253-017-8112-0.

[15] Zhou H, Abuduaini A, Xie H, Kang R, Suo F, Huang L. The complete mitochondrial genome of wood-rotting fungus Xylaria hypoxylon. Mitochondrial DNA B Resour. 2019, 4. 3848-3849. doi: 10.1080/23802359.2019.1687025

[16] Gilchrist CLM, Booth TJ, van Wersch B, et al. Cblaster: a Remote Search Tool for Rapid Identification and Visualization of Homologous Gene Clusters. Bioinforma Adv 2021; 1:1–19. https://doi.org/10.1093/bioadv/vbab016
